# Supplementary material for: Perspectives about social support among unmarried pregnant university students in South Africa
Source: PLoS One. 2023 Apr 24;18(4):e0284906. doi: 10.1371/journal.pone.0284906 (PMC10124874; doi:10.1371/journal.pone.0284906)
Supplement: S1 File — (PDF) [file pone.0284906.s001.pdf]

Interview 1

Interviewer: Can you talk about your pregnancy experience.

Interviewee: It is fine generally, but when it comes to issues like being at school it kind of not easy because you have to attend to the doctor or clinic, a lot of things you need to do to take care of you and the baby, so it's not easy at all, you know like being away from your family, people who support you, so yeah, it's not easy.

Interviewer: Is this your first pregnancy? If not how is it different from the previous one(s)

Interviewee: Yes, it was my first pregnancy

Interviewer: Apart from your own experience, what do you think pregnancy should be like? -Did you think you would experience any challenges?

Interviewee: I didn't think so. I didn't even plan to get pregnant, it just happened. So, if something hasn't happened to you, it's difficult that you can think about it. You see pregnancy is something else, I don't know how to put it, like, when you are not in the situation, the way you look at that situation is different from the way you see it when you are in that situation. Also, time for study. You don't do things as usually. You don't study as you are supposed to, you don't feel like it. You are always feeling sleepy. And you don't feel like going to lectures.

Interviewer: Describe some of the challenges you face in your pregnancy? As a student?

Interviewee: Being at school and being pregnant and being away from your family. Ok, for us as Africans it's hard to tell your parents that you are pregnant but when you tell them, they will just be angry at you for like two days and afterwards they will become so supportive. Like everyone was so supportive. Because of that, it was - not easy – but better than not being supported at all. So, you know there is support from your family, from your friends, and all your loved ones and just everyone around you. My partner was so happy when I told him I was pregnant and he was so supportive.

Interviewer: What do you think the people around you can do to help you cope with pregnancy? Why?

Interviewee: Just to feel loved means a lot to me.

Interviewer: What is the age of your pregnancy? At what stage of pregnancy do you feel you need the most support from family or friends? Why?

Interviewee: It was at the end. When you feel like the baby is about to come, you have to prepare for everything, like financially. For school work it's very hard. Because at the end, I have to attend clinics than before so the time, like almost every week you attend clinic so the time for attending clinics and everything, you are using up the time for studying. My friends did take notes for and tell me submission dates.

Interviewer: Now that you have the baby, do you feel you need more support now than was needed when you were pregnant? When did you need more support?

Interviewee: It is now. Because you want to give the best to your baby. Before you deliver, you need support, not financially, but you need support. But after, you need more support financially. You have to provide for your baby.

Interviewer: How/ where are you going to have your baby from? Why?

Interviewee: I was staying in res and went home to deliver. I moved home two days before I gave birth because my doctor set an appointment for C-section on the 18th but I got labor pains on the 16th so I delivered on the 17th.

Interviewer: And how are you doing yourself?

Interviewee: I am fine. I had a C-section and couldn't walk in the first week but am ok now.

Interviewer: How was it staying in res, away from your family, while pregnant?

Interviewee: Am used to it, I have been away from my family since high school. So am used it. Nothing changed just because I was pregnant. They still call me every day to check up on me and how I was.

Interviewer: What kind of support do you feel you will need the most after (now that) you have the baby?

Interviewee: It is moral support. Because sometimes you just feel like lonely and you need to talk to someone. But when I was pregnant, I was fine. Although I didn't plan my pregnancy, being supported by the people around you make you feel good. Yeah. My mom also brought someone to take care of the baby and she pays that person.

Interviewer: Who is going to help you during that period?

interviewee: The baby is two weeks old and is currently at home with my parents while I have moved back in res so that I can study and write exams. I do miss him a lot but I have to study for my exams. My mother asked someone from around home to look after the baby while she is at work and she pays that person.

Interviewer: What do you think about male partner support during pregnancy? Clinics?

Interviewee: It is very important. It is the support that you feel you need the most. Although your family is around but if your boyfriend is not around, it's not good**.**

Interviewer: To what extent is your partner involved in your pregnancy?

Interviewee: He encouraged me to do things like study. He made sure I ate. I was at res so I had no chores and no one to cook for. He came with me to the clinic on my second appointment, when I was about four months. It means a lot to have someone with you. But they didn't ask him any questions or asked him to do any tests.

Interviewer: From the support needs you have already mentioned, which ones do you feel are the most important to you? Why?

Interviewee: It feels good to have people around and knowing that you are loved. Although you made mistakes - not that a baby is a mistake – but being pregnant while you are studying, being something that you have not planned obviously, so knowing that although you have made that mistake, but people haven't abandoned you. People around you like your loved ones and your family- that they still love you.

Interviewer: Which ones has your partner taken up or has been doing the most?

Interviewee: He still encourages me to study and just knowing that you are loved it's something that you need the most.

Interviewer: Which ones would you like your partner to give you?

Interviewee: At the moment everything is ok. You know every relationship has its challenges. But at the moment, am satisfied, although there are those challenges, but every relationship has challenges.

Interviewer: What does it mean to you to have your partner support you in those ways/ how do you feel if they don't show that support?

Interviewee: As I have mentioned before, feeling loved (laughs). It's something that I believe is more important than anything. Because feeling lonely, you get stressed, you get depressed.

Interviewer -How does that make you feel? Loneliness?

Interviewee: I don't know how to define it, but it's not a good feeling

Interviewer: How does/did the involvement of your partner influence the decisions you make concerning your pregnancy.

Interviewee: When I feel like it's something serious. I haven't thought of aborting because am a Christian. I think my religion is the one that was guiding me what to do more than my boyfriend.

Interviewer: Do you think there is a positive relationship between partner support and mother and child health?

Interviewee: No. People are very different. I am a person who accepts everything that life throws at me. So, had he not been supportive, it would be the same. Not the same, but I would have accepted that the situation is like this, so for the sake of my baby especially, I have to be okay,

Interview 2

Interviewer: Your experience being pregnant as a student and your support system, the people around you and the role they have played during your pregnancy. Talk about your experience as a pregnant student?

Interviewee: At first it was kind of a scary thing just that I never imagined my life me being pregnant in school, but then as time went by I was quite excited about being a Mom something like in future you have something to hold on, to give you a reason of waking up in the morning every day, I have got a mouth to feed so! Really can't` be sleeping. I have learnt to live with it and am quite happy.

Interviewer: Is this your first pregnancy?

Interviewee: Yes, it is

Interviewer: And how is it going so far?

Interviewee: So far so good, it has been challenging because me and the Baby`s Father broke up.

Interviewer: At what stage in your pregnancy did that happen?

Interviewee: At 5 months, and right now am about 5 months and two weeks and I was like maybe 4 months and 3 weeks, so we broke up. At first, I was heartbroken and thinking how I will do this but then as time went by you won`t be the first person to become a mom and having my Mother`s support gave me lot of strength. She was always the one like how is the Baby, go to the clinic and I was like I can do it for the sake of my Baby I have to be strong, he chose to leave us so it's up to me now and what I do.

Interviewer: So, knowing that you have accepted your new role as a single Mum, what are the challenges you have faced the last 5 months, from the time you discovered you were pregnant up to now, what are some of the things that have been difficult?

Interviewee: First of all, the biggest thing I was telling my Mom that am pregnant that was the biggest thought, since am the only girl at home, thinking she`s gone scream at me, disappointed and all that stuff and what happened was that when I was telling my boyfriend am pregnant, he

asked me to do an abortion and I was like what the hell it's his second child. So, losing my child I guess he already has one, so I told him no I was not gonna do it, so we argued, I guess somehow, he adjusted and he was like okay she`s not going to do it so we continued dating and at the end of the day he still dumped me. So, telling my mom was like a scary thought and her reaction kind of like surprised me, she was so calm and she was like there is nothing surprising it happens, all you had to do was mention it a lot sooner. So that I know the Father of the Baby because you know they run away

Interviewer: At what stage in your pregnancy did you tell your Mother?

Interviewee: I was 3-4 months.

Interviewer: And all that time she did not notice?

Interviewee: She did notice, am more like a mummy's` girl, I do not sleep in my own room, I sleep in hers, so when she goes shopping I would usually as her to buy me pads, so this month she noticed I did not ask and I was like I had money last Month. Then she could be like Lerato you skinny but stomach, no, and I could be like no it's something I ate, I kept on making excuses then I couldn`t hide it anymore so.

Interviewer: You told her and her reaction was calm?

Interviewee: She was pretty calm, the only thing I think that upset her was like I kept quiet but the rest she was calm, I didn't expect this, if I knew I would have told her a lot sooner.

Interviewer: And at school? before we talk about school you were staying home the time that you told her?

Interviewee: No, I was actually here, I was already here.

Interviewer: So, you went home to tell her?

Interviewee: Actually, my Aunt told her, then she called me, then we talked about it.

Interviewer: And being away from home and staying alone?

Interviewee: It`s quite a challenge, I miss home a lot and sometimes people say may be its I don't know but in Zulu they say it's (Yathandisa) I don't know how to say it in English and most people say it us (Zulu saying) (Uthandiswa) it's like loving something too much, you know sometimes we have cravings and I always wanna be with her, it's a difficult thing being away from home but I had to be here to study.

Interviewer: And your school work?

Interviewee: Actually, am doing pretty good now, I have been having problems especially with my biology but then now it's just me and my exams then am gone.

Interviewer: So, what do you think when you look at already you have told me that you Mom has been very supportive but is there anything else that you feel you long for any type of support you feel you would love to receive from you loved ones that you are not received?

Interviewee: Actually, I would love to receive support from my boyfriend but since he`s not here there is nothing I can do about it.

Interviewer: What role do you imagine he was going to play in your pregnancy? what do you feel you miss now that he`s not in this picture?

Interviewee: Besides my Mom I just needed to ask him, to hold me, feel my Baby kick, besides myself, call me, to find out how is the Baby, someone to share if am going to do a scan to be there with me, if it's possible to show pictures around, to talk about things.

Interviewer: So you told me you are 5 months and 3 weeks, do you feel you needed support at the time of your pregnancy or you`ll need more support as the time the pregnancy progressor may be after the Baby is born?

Interviewee: I think I needed more support at the beginning

Interviewer: why?

Interviewee: It was something new to me, I kinda needed someone to hold my hand and say it's gonna be time, am gonna be there for you, don't worry, we`ll get through this together but now that she`s gone I feel it's up to me to whether continue with life or continue living in the past

and decide on what you`ll do, how you`ll make it and if the Mommy is supporting you then its fine, its perfect. Live with it accept it.

Interviewer: So where are you going to have the Baby from?

Interviewee: Around Durban

Interviewer: And you`ll be home with your Mother after the Baby is born?

Interviewee: Am not sure cause I think I will still be in Durban because I give birth in September and my Mom will eventually travel to here.

Interviewer: Where is your Mom?

Interviewee: Dundee

Interviewer: Dundee is outside Durban?

Interviewee: Yes

Interviewer: So, she will travel to come and be with you and take care of the Baby and are you going to continue with school since September is next semester?

Interviewee: Yes, my Mom said “give me the child then you go back to school “that’s how it’s gonna be

Interviewer: So, you`ll come back to school and she`s gonna take care of the Baby?

Interviewee: Yes

Interviewer: What kind of support will you need after the Baby is born? How do you think people around you can help you after the baby is born?

Interviewee: When the baby`s born I don’t think I will need much help because my brother`s son he`s now 4 and he pretty grew up in my arms so I quite know a lot about babies, how to feed them, how to change nappies and how to wash them.

Interviewer: So, you don’t think you`ll need that much help?

Interviewee: Well of course, my mom will be there I do think so.

Interviewer: And in terms of finances?

Interviewee: I'll still be in Durban when I give birth cause am giving birth in September. So, my mom will travel here. My mom said "give me the child then you go back to school" and she's gonna take care of the baby...In terms of finances my mom will support me. I don't know about my child's father whether he'll be there or not.

Interviewer: What difference was going to be there if the father of the child was going to provide the money?

Interviewee: I think my child would have more of what he`s gona have if it`s a he or she, and the father`s love.

Interviewer: So, you have already mentioned that your mom is going to help you take care of the baby, going back to the partner, do you think it is important to receive support from your partner?

Interviewee: I think it is

Interviewer: Why?

Interviewee: It kinda gets lonely sometimes

Interviewer: And how does it feel when it`s lonely?

Interviewee: It's sad feeling actually sometimes you get mad at stuff and you really can`t talk to your mom so you just hold it to yourself internally.

Interviewer: From the support needs you have already mentioned for example emotional support from your mom, financial support too, what other types of support do you think, I mean from those you have mentioned, which one do you think is the most important? When you look at your environment here at school, your friends, your school work, which one do you think is the most important one to you?

Interviewee: My mom`s support because I already lost both parents so my mom.

Interviewer: Would it be important to you to go back to antenatal, for scans with your partner?

Interviewee: Not necessarily important but actually seeing his photo on the scan would mean something to him.

Interviewer: And what would it mean to you?

Interviewee: Seeing the baby move in my stomach means a lot, knowing that my child is alive beside her or him knowing that my child is alive beside her or him moving in my stomach, actually feeling the heartbeat, the aims etc.

Interviewer: And what would it have meant if he was around?

Interviewee: I think it would have meant a lot.

Interviewer: Do you think there is a positive relationship between your well-being and the baby`s being and the involvement of the father? Do you think the involvement of the father determines the mother`s and the baby`s well- being during and after pregnancy?

Interviewee: I think so, it was quite an emotional break down when we broke up so at some point I felt like I could do anything, anything to get rid of the stress, drink alcohol, smoke etc and that too would harm my baby so I had to take it slow, and if I felt like crying I would always do but then I had to try and stay strong cause drinking and smoking would harm my baby so I had to adjust. It’s a painful process but now am like his gone, there is nothing I can do about it but just to accept it.

Interviewer: Thank you so much for your participation.

Interview 3

Interviewer: Please share your pregnancy experience?

Interviewee: So far being pregnant at a young age is not an easy thing cause we as young stars we like partying, drinking, wearing short things so when you are pregnant when you are young life tends to be not like it was before right now I have to wear something long, comfortable for the baby and also for me, the things I eat, I don't have to go to party anymore, Some of my friends ignore me due to the pregnancy because they don't wanna walk with me to school and I also see myself as totally different from them. At first, I felt so neglected now I have accepted that am pregnant and things won't be the same. I usually walk alone. Some friends will support me, some will not.

Interviewer: So how does that make you feel?

Interviewee: At first, I felt to reflected, now I have accepted, my boyfriend is a totally supportive man told him everything but now things are cool, it's not the same, I usually walk alone, the supports me tell me no its fine, things won`t be the same now, he`s supporting me every way, I have also accepted that am pregnant and things won`t be the same. Some friend`s will support me, some will not, some will see me as a remainder since they aborted their babies, am a remainder of the bad things they have done before, so the easiest way to forget the bad things they have done is to neglect me.

Interviewer: And how does it make you feel when you feel as though they have neglected you?

Interviewee: At first, I was hurtful, but now am okay I have accepted real friends will stick with me, the fake one will just go so at first it wasn`t an easy thing but now am okay everything is fine.

Interviewer: So, this is your first pregnancy?

Interviewee: Yes, it is my first.

Interviewer: If you don't mind, may I know your age?

Interviewee: Am 20 this year

Interviewer: So a apart from your own experience you`ve told me that pregnancy has changed your life you can`t wear a certain thing, you can`t go to parties, certain friends of ours have decided to ignore you, but when you think about it before you even got pregnant what did you think pregnancy should be like? What do you think should be normal for a normal pregnancy?

Interviewee: Should be normal like what?

Interviewer: What do you think pregnancy is supposed to be like? How do you think it's supposed to be?

Interviewee: I thought it was something not big, just the thought of having a baby, nothing much, that you have to stop certain things for that particular period of time when you regnant but when I became pregnant it was different like it changes the rest of our life it's not like that period only, it changes the rest of your life, now you`ll have someone in your life , not just someone but someone in a different way, someone who belongs to you so that is the long life process, not easy to accept but it also depends on what kind of a partner you have to strong for your baby but it does matter what your partner thinks about you, it does matter the support you get from him, matter a lot. I used to judge people that aborted their babies, I used to judge them a lot but now I understand accepting a pregnancy is not easy at all, accepting that you will have a baby, you`ll now going to be different with others, when you talk about your boyfriend you also talk about the third person, changing nappies, buying milk, your body changes, a lot changes. Before I only thought about that particular period of time but now I know it's a long-life process which is not easy to accept and judging from the one who aborted the babies I also think they were being selfish but now I understand, am not saying it's good am just no longer judging them because it's not easy especially if you are not having support from the baby`s father.

Interviewer: So, you have talked about your life changing and your new role as a mother, how far along are you?

Interviewee: Am 7 months, next week I will be 8.

Interviewer: So, the last 7 months, what are some at the difficulties you have faced at home, school, and in your life?

Interviewee: I don’t have many problems, I am okay financially, I am okay, am not vomiting, the only thing that has changed is the stomach big and the boobs, I don’t like them anymore, the bra is tightening me up, doesn’t fit me as at used to before but financially am okay. Emotionally, am happy now.

Interviewer: Where you always happy at the beginning when you just found out.

Interviewee: I wasn`t happy but I knew I was keeping the baby.

Interviewer: And school, how are you doing at school?

Interviewee: Am okay, am managing everything. I am not having swollen feet everything is just normal, am happy with how things are. I am excited with a lot of energy, am not like the other ones (she then narrates a story about her almost blacking out at the mall)

Interviewer: You have already mentioned that your boyfriend has been very supportive, how long have you been in a relationship with him?

Interviewee: 2 years

Interviewer: A part from him who else has been supportive?

Interviewee: My family

Interviewer: How did your parents take it

Interviewee: Not so bad, not the way I thought, it was not what I expected I thought things will be much more difficult, I don’t have a mother, I thought my father would stop paying my fees, stop giving my monthly money, I thought he won`t support me but he`s fine, he`s just giving me the courage to study. My other friends are supportive, checking on me every day.

Interviewer: So, is there any area that you feel you may be lacking in support? Is there any other type of support that is not being given that you would have loved to receive?

Interviewee: Am happy with the support am receiving

Interviewer: So, who’s your greatest source of support?

Interviewee: My boyfriend

Interviewer: So, does your boyfriend come with you to antenatal or to doctor`s appointments?

Interviewee: Yes, we go together, I thought when I got pregnant he`ll be leaving me at home, no walks together, we won`t go to the mall together, we won`t go to the restaurant but no things are still the same, he still wants everybody to see me with him.

Interviewer: Do you feel it is important to receive support from your partner whilst you are pregnant?

Interviewee: It is important

Interviewer: Why?

Interviewee: "We are in this together" the things he used to tell me, "we are pregnant together". So, this gives me the courage, what would have I have done if I was alone? Sometimes I don't have money for travelling, clinic, am in pain from injuries so the support from him is much important

Interviewer: Is it much more important than the support you receive from your family and friends?

Interviewee: No!

Interviewer: Which one is the most important?

Interviewee: The friends and family are the most important because especially for family they will be there for the rest of your life so the support they give is more powerful than from the boyfriend.

Interviewer: But don't you think your boyfriend will also be there for the rest of your life? How would you feel if he was not supportive? How would it make you feel if he wasn`t as supportive as he`s been?

Interviewee: Things were not going to be the same but for the sake of the baby I think I was going to hang in there using the support from the other friends and family I was going to be

strong. I think I would have stood up because there is nothing much he does but the support from him is there but then people change maybe he won`t be there for the rest of my life, but the way I see myself cause am studying am doing my third year am about to finish so I will get a proper job and still manage to do things an my own. So, the family`s support is the most important.

Interviewer: So, you`ve said everyone around you has been supportive, so is there anything else that you long for, that you feel they should do? Do you feel all means are covered in terms of support during pregnancy?

Interviewee: I think everything is fine and also the baby`s father is supportive, and also my mother in-low is supportive buys me healthy food when am back home to make sure that am okay.

Interviewer: So, you have told me that you almost 8 months now, and when you look at the last 8 months from the time you got pregnant to now when do you feel you need the most support was it at the beginning, in the middle, or is it at the end? When do you think you need the most support?

Interviewee: I think it's at the end, am now scared of giving birth.

Interviewer: How are you going to deliver?

Interviewee: Natural birth, and I hear natural birth is painful so the time I will finish my exams I think the stress will be much higher because am scared cause sometimes I even cry alone there is still one day which is coming which won`t be easy but I think that is where I need the support.

Interviewer: So what kind of support do you think you need this time that you are approaching the delivery? What kind of support do you feel its emotional, or?

Interviewee: Emotional support. My boyfriend saw me that I am kind of stressed he bought me something to eat and a top to cheer me up still [yes you were saying your boyfriend bought a top] yes he bought me a top but could still see that still am not okay emotional support is much more important because it cheers me up, "you know everything will be fine" you see people are not the same we don't experience the same pain, there is one of my friends who gave birth to twins she was 7 months it was a natural birth so it tells me I know it will be fine

people are not the same, yes it is painful but people are not the same you may experience different things [okay]

Interviewer: So where are you going to have your baby from?

Interviewee: Aaah the private hospital in Richards bay [Richards bay is your home, so you`ll go home] ……[are you staying in res] no am staying with my aunt in Chesterville [okay]

Interviewer: So, who's going to take care of your baby who going to help you during time when having the baby?

Interviewee: Oh, my aunt, my sister`s, mom [ she`s going with you to Richards bay] she said during, I think during the month of delivery she`s going to take a leave [okay] because we don't know the date [okay] she`s going to take a leave then so if anything happens [she`ll travel with you to Richards bay] yes

Interviewer: And after you deliver …… what`s going to happen?

Interviewee: Oh, we don't have a, we have a helper at home [okay] she also suggests, "no, it`s fine I should leave the baby with her she would like to help the baby grow''

Interviewer: So, are you going to leave the baby in Richards bay or you'll come back with the baby to Chesterville?

Interviewee: I will leave her in Richards bay

Interviewer: Who's going to take care of her in Richards bay?

Interviewee: The helpers then during weekends when I have money go back home [okay] yes

Interviewer: Uh, so do you have anyone else apart from the helper, do you have a step mom?

Interviewee: Oh …no the step mom also said she was going to, she was fine with hiring someone but no we said no its fine, but also the granny of the baby she also said she was going to make sure that everything is fine, she going to take the baby also when she`s off from work [okay]

Interviewer: So what kind of support do you think is very important after the baby is born? Do you feel its emotional or financial, what would be the most important for you after the baby comes?

Interviewee: ………… mostly I think it is financially

Interviewer: Why?

Interviewee: The baby needs so many things so as to grow in a good state; nappies, warm clothes, milk, formula [baby food] since am not going to breastfeed so financially it will be much required from him. [She gives an example]-cause my cousin gave birth in April and the father of the baby hasn't given her a single cent since she gave birth, since she fell pregnant, she hasn't received any money from the baby's daddy and telling me things are difficult, so financially it is so important, so financially it will be much, because my cousins gave birth in April and the father of the baby hasn`t given her a cent since she gave birth, since she fell pregnant she hasn`t received any money from the baby daddy and telling me things are difficult it's not the same, now the ….. her mother has to make sure that the baby has nappies … but she`s breastfeeding but still things are not the same so financially it is also important [okay]

Interviewer: So, who's going to provide the financial support for you and the baby?

Interviewee: The father of the baby [will provide everything?] yes [okay] but I also made some savings just in case you never depend on that I have made some savings just in case things don't turn to what I thought towards what he`s saying but I do have my own savings [okay]

Interviewer: Does your partner come with you to clinics, I asked that already?

Interviewee: Clinics, no. You asked about the doctors [yeah] clinics, no I was suggesting, no he doesn't come with to the clinics

Interviewer: Whose decision is it for him to stay? It's yours?

Interviewee: It's his, not me, he said he is not interested in staying in the clinic, even when I say you don't have to sit with me, clinics is just something else.

Interviewer: Do the people at the clinic, ask the nurses about your partner?

Interviewee: No [they don’t?] they don’t.

Interviewer: Which clinic do you go to?

Interviewee: Chesterville clinic [okay] but when am at home I do go to ours [okay]

Interviewer: So …. Ah your partner has been emotionally supportive and he`s also been financially supportive you have mentioned, isn`t it … ah so what does it mean to you to receive support from your partner?

Interviewee; I highly appreciate it, it means a lot you know ah I also tell myself when I give birth the first thing am going to say and am going to thank him is that he has been the best 9 months of my life, so the support from him is incredible [okay], I do feel if am lucky because others don’t get this support am having, really

Interviewer: Do you think it would have been different had not been this supportive? Do you think your health and the health of the baby would have been affected?

Interviewee: Yah, I think yah it would have been different cause the other day for the first few days we found out that am pregnant I told him that next semester I think am not going, I won`t go back to school, I wanna stay with my baby …. Ah no he refused, no you`ll go back to school, everything will just go back to normal so for him my education is also important

Interviewer: And your health, do you think your health would have been affected if he didn`t support you emotionally, physically, how do you think you were going to be if you were going through this alone?

Interviewee: It would have been not easy because sometimes when you are walking in town people are like, “where is the father, “ you know, like people how they are where is the father of the baby, now you walking alone, where is he, “ but inside me I know that am with him, he supports me but when he would have been not there it would have hurt me you know to know that he`s not here, people are laughing at me, so that`s not good for the baby, yah, they told me that the things that you feel the baby also feels them so that’s not good for the baby [okay] yah you should also stay excited.

Interviewer: Okay so do you think there is a relationship between the health of the baby and the mother and support from the partner?

Interviewee: Yes, there is. Yah, it is important sometimes when I am not with him may be not talking you`ll find that the baby`s not moving but when I am with him the baby gets much more hyper.

Interviewer: Why do you think that happens?

Interviewee: The baby can feel his father [okay] things are just fine, he also gets excited yah. [okay]

Interviewer: Thank you so much

Interview 4

Interviewer: Thank you so much for you time, I really appreciated your time. Ah, please just briefly talk about your pregnancy experience

Interviewee: Yoh. Okay the first time, I discovered that I was pregnant it wasn`t a big surprise [yeah] ahm ……then obviously the months start kicking in your third month, your fourth month, you start having the nausea, …. Never really went through the whole morning sickness thing but I started feeling the whole nausea sickness, went to bed, am started to feel excessive anger towards my boyfriend and towards people around in general, am I only told the people directly like when I was 6 months pregnant actually I think [oh, no one noticed until then] no [okay] and then around about the 6 months, I think the pregnancy started taking its toll on my relationship with my boyfriend because we fought like cats and dogs, I would hate him so much at one point it would just be like just leave, get out of my face, I hate you, the next please comeback and he didn`t understand all that you know, he`s never had a child, okay he`s got a child but never lived with a pregnant girl or whatever [oh] so am the first experience that he`s going to experience and for him it was, a serious emotional rollercoaster for him and myself.

Interviewer: Why do you think you were fighting like cat and dog?

Interviewee: I don't know I think it was just the whole …… the circumstances surrounding the whole pregnancy, that financially he wasn`t ready, financially I wasn`t ready and there we go my friends you guys are going to have a child and you need to start making preparations for him to go home and pay and this and that and you know and for him things ….. he does tender so he`s his own business person. So, for him things financially weren`t really following too smooth and now he needs to pay for the doctor and then medication and certain foods I want I wake him up at certain hours I mean at some point, I think two- three months back to like 7 days he would leave by myself at home I had to sleep by myself because he couldn`t cope with ahmm …. With the fighting and the cursing, everything he couldn`t just cope, he wasn`t coping. And so eventually, I don't know what happened he just turned to leave and he basically now understands, he`s always there now, irrespective of whether we are fighting or not he always back, he`s on low now [okay] yoh emotionally it's not what you expect whilst with me you know you look at people, oh you pregnant, oh how nice, " but actually when you get to the real deal

and everything ah … for me it wasn`t all so glorious, it was stressful, very stressful to a point that for my last year a campus the doctor was set to meet me which is Monday, I had a very high Bp pressure,  okay it doesn`t sound right …. So, I had a very high blood pressure and he wanted to admit me in hospital. I just went through a heck up now and it’s gone down a bit.

Interviewer: And school …. School work how have you coped from the beginning of your Pregnancy?

Interviewee: Ah school works been fine

Interviewer: You have studied, passed your tests?

Interviewee: Yah it hasn`t really been an issue from last year. In fact, so I have written my exams, the exams that I have written I’ve passed ….. I have submitted my assignments, I have passed, gone through my exams, which is just [everything is fine] its fine, I have no problem at school.

Interviewer: And if I may ask how far along are you?

Interviewee: I am 38 weeks pregnant [okay so you almost there] very close (laughs)

Interviewer: How does that make you feel knowing you are nearing the end?

Interviewee: ……. I don’t know …. I feel a little bit sad that I didn’t really get to enjoy, this as much as I thought I would, but …..

Interviewer: Why do you think you didn`t enjoy as much as you thought

Interviewee: Because I have had so many challenges, it’s been a real rough rollercoaster ride for me, ithasn`t been good, am not gonna lie and say, oh it’s been very nice”, it hasn`t been very nice it’s been really rough and it’s been a very eye-opening experience you know.

Interviewer: What are some of the challenges that you`ve had that have contributed to it being rough?

Interviewee: You could say its school, you could say it’s the relationship aspects of it that it takes the emotional hormones into everything (laughs) it’s just wow too much.

Interviewer: So, this is your first pregnancy?

Interviewee: Yes

Interviewer: And before you got pregnant what did you think pregnancy was like?

Interviewee: I thought it was lovely, I thought, "oh you get to be treated like a queen compared with soldiers, this and that, this and that, "I never really thought that. Unfortunately, you need to toughen up, you need to make sure stick it through exams, you need to pass your exams you need to cook, you need to clean, you need to wash you need to be on the board all the time [okay] (chuckles)

Interviewer: So, what do you think the people around you could have done to make this easier for you?

Interviewee: They could have been more understanding.

Interviewer: When I talk about people am talking about everyone around you, your friends, your family who did you think was not as understanding?

Interviewee: I think my boss wasn`t understanding, she wanted to, she was trying to but I don't think it you have never a child you never really know until you have experienced it yourself how it actually feels like ….. uhm my boyfriend okay he`s trying to live and he`s kind of he`s absorbing everything as and how I do it to have, he`s just taking it as it is.

Interviewer: Was he always like that? At the beginning.

Interviewee: He was normal, he`s not really one, he`s not a person that likes fighting [okay] he`s very calm, very peaceful "I don't want to fight with you'' I just came home to sit with you, I don't wanna fight with you. he`s very chilled person (……) and also him the only way that he could cope with it was just to run away.

Interviewer: So, at what stage of your pregnancy do you feel you needed the most support? Was it at the beginning, was it in the middle or is it now at the end, when do you feel pregnant people need the most support or you from your experience when do you feel like you need a really strong support system in your pregnancy?

Interviewee: I think from the 6 months onwards I think that when you need, the 2nd after the end of the 2nd (trimester) I think that's when you need the most support.

Interviewer: Why would you say that?

Interviewee: Cause that when you hearing you end, one, you have got so many things to do, you have got school things to do, you have got doctor`s appointment consistently one after the other, you have to buy this, get this, get that, get this and that's when you need a more rigid support system [okay].

Interviewer: So, what is the most important type of support to you?

Interviewee: Emotional support

Interviewer: Why do you say that?

Interviewee: Because if you are emotionally you are broken-down, then you crying because I think that's when I cry, you constantly crying, if there is somebody that actually cares, now why are you crying. "then you just feel like oh my GOD what is wrong with me? You know, you just feel like the world is coming to an end.

Interviewer: So where are you going to have your baby from?

Interviewee: In Richard`s bay [so you are going to travel to Richard`s bay] yes

Interviewer: You are 38 weeks when are you planning on doing that?

Interviewee: I think next week 4 my doctor doesn`t say I must come back

Interviewer: And how are you having your baby?

Interviewee: Natural birth [natural birth, okay]

Interviewer: So, who's going to help you when the baby comes?

Interviewee: My mother and my father, my boyfriend, my brother and my sister [okay, so your whole family is in this with you].

Interviewer: What type of support do you think or let me say after the baby comes (……..). So you have told me emotional support is the most important to you during pregnancy what about when the baby comes what do you think will be the most important support? Do you still feel emotional support will be at the top of your priorities or maybe there are other types of support like financial support or other things that you feel would come first then?

Interviewee: I think for me it's still be emotional support because for me I knew that I had my mother I only told her that am 6months pregnant and from then she`s been there till this very point…… I think if you have that emotional support everything just falls into place, the finances and everything else don't really matter.

Interviewer: What do you think would have happened if you didn`t receive that kind of emotional support from your family?

Interviewee: ………aye ……. I have no idea, I don't even want to imagine that or take myself back [it's that important to you] yes. Knowing that, you have got your mom, that you`ve got your dad and I mean my parents went through to the extreme from they call me at night to find out if am okay, did your boyfriend come back, "no okay don't worry will call you to find out if you are fine, okay you know, I know I have got my parents though they are in Richard`s bay but I know I have got them if anything ought to happen though they would willing to jump in the car and drive to come see me.

Interviewer: So, what do you think about partner support during pregnancy? How important is partner support?

Interviewee: Very important

Interviewer: Why?

Interviewee: As much as you are the one carrying the child, you can't go through everything by yourself. I think if you don't have your partners support, you can end up having resentment towards him, including the child - somebody who has no contribution towards the baby is painful. I think is very, very important. When my boyfriend left me hello oh the way I hated him I would curse and send him sms and threaten him telling him if you don't come back you`ll

never see your child and such and such and then and now the whole kind of burden has been taken and we don’t have to deal with that.

Interviewer: How does it make you feel to have support when you think of others who don’t have the same support you don’t have?

Interviewee: Treasure it, though it’s not the kind of support that you are envisioning but that little support that you are getting is remarkable knowing that the person will always be there for you irrespective of what has been said and done, that is priceless [okay].

Interviewer: So, when you have the baby are you coming back next semester.

Interviewee: Yes

Interviewer: Who’s going to take care of the baby then?

Interviewee: Ah am weighing my options between a nanny and day care. Bback in Richard`s bay or here in Durban] here in Durban [okay], I don’t think I will need much help home. [okay] [so you`ll come back with the baby and look for help] yes.

Interviewer: And if I may ask who’s going to pay for that help is it you is it your boyfriend?

Interviewee: It is, I most likely be between myself and my mother.

Interviewer: So, what about the clinic aspects, you know pregnant women have to have a number of visits with the clinics, the doctors, antenatal, have you had your boyfriend come with you to clinics at any one time?

Interviewee: Yes, he has

Interviewer: What kind of appointments, is it scans, is it doctor`s appointments or is it antenatal?

Interviewee: Scans [okay] a gynecologist we went to one the one we had in Durban, he`s been to that, ahmm the other ones he`s paid for

Interviewer: And antenatal?

Interviewee: Am not attending those [you are not attending antenatal okay]

Interviewer: You have told me your partner is very involved in the pregnancy, okay, so from the support needs you have mentioned, you have also told me emotional support is the one that`s most important to you, so which one has your partner given the most from all the types of support we can talk about, emotional support, moral, financial which one has your partner been the most active.

Interviewee: I think emotional cause financially he hasn`t been there (laughs)

Interviewer: Why it may ask?

Interviewee: Because he hasn`t really had his coins I suppose in the right package as he`s still trying sort his monies out [okay but that`s fine with you?] it's not fine but I mean he`s been contributing what he can he`s been praying for those doctor expenses, he`s been praying for buying this and that, it hasn`t been to the level that I expected it to be [okay] but emotionally he`s turned, he`s done a complete 360 [okay when you say he`s done a complete 360 you mean] it wasn`t that way in the beginning] yes it was, a bit then it just turned I think the moment I just started being this monster towards him he didn`t understand it and he was like "what is this and he left".

Interviewer: Was it difficult for you when he wasn`t there?

Interviewee: Yes, it was [okay]

Interviewer: How did that make you feel?

Interviewee: It makes me feel terrible

Interviewer: Does the involvement of your partner influence the decisions that you make pertaining you and your baby?

Interviewee: Yes [you run everything by him] yes

Interviewer: Do you think there is a positive relationship between partner support and your well-being and the baby's well-being?

Interviewee: Yes definitely

Interviewer: Why do you say that?

Interviewee: Because the moment you don't have him around, you start to get yourself worked up, your BP goes up, like this weekend he was gone for a meeting and I couldn`t get hold of him and by the time I had gone to the doctor I hadn`t really got hold of him but I mean I had got hold of him like 10 minutes before the appointment and I was so worked up I think that's one of the reasons my BP was so high and it had taken some time to go down day by day the moment I don't get hold of my boyfriend I just go (ambulance sounds) (laughs) I need to talk to you know, I need you here now and if am not fine with him I don't feel okay.

Interviewer: Thank you so much for your time I really appreciate

Interview 5

Interviewer: Can we just talk about your pregnancy experience. Your whole experience how it has been being pregnant at school how has it been like?

Interviewee: Well for me it hasn’t been that bad like for most people cause like right now am almost 7 months pregnant and most can’t even tell that am pregnant but it it’s like a lot of pressure cause am in my first year so there is that thing which eish won`t the childhood be bad and everything and the effect that it had on my parents cause when my father found out that I was pregnant which was recently he cried like a baby [WOW] yeah my mom couldn`t talk like a week or something but then she finally opened up to everything yah ….. as for friends I don’t really do that whole friendship thing am not very good at it but the people that were close to me argh I think I sort of pushed them away when I found out I was pregnant cause I thought they were gonna judge, most did but yah so most judged me [and how did that make you feel] it you feel that people are judging you] it angered me it angered me cause you know what happens that not that I think am better because I kept the baby but majority of the girls in the neighborhood ah don’t keep babies, like abortion is the king to do it’s like the only option so when you keep your baby you kind look dumb as if like, ah so you wanna be held back you trying keep the baby daddy and everything and you know the stigma that follow the pregnancy so yeah the option for me was like about the child and that’s it there is like nothing else after [yah] so when I choose to kept it came as a stock, especially because a lot of people did not expect it from me like a lot still can`t believe it.

Interviewer: Okay why do you feel people didn`t expect it?

Interviewer: Well, I think I have a tomboyish background and am not gona say am a church girl but my father is a priest [okay] so yah and I have been, cause, ah, let me not say they were not expecting it …… let’s say they expected it like a long time ago and then it didn`t happen, so and then they were like oh (sulks) there is no hope for this child cause you know when people describe you carry cause most of them are like, “you don’t even have a boyfriend you probably a lesbian and everything [okay] yah so it was something like that …. So, a lot are still in shock and a lot like right now they still can`t like lock at me in they still can`t like lock at me in the eye especially my father. So you see now I think he`s [are you staying home or you are on campus] I

stay at home [okay] stay at home it's still difficult for him to stay in the same room as me like he`s trying you can see that he`s trying but he`s so hurt like it's so unbelievable cause for him, I think actually with every girl in her Father`s eyes will always be three years old you understand so if was something like that and ….another thing like for me growing up, am not gonna say I was the perfect child, but I never actually did anything that you know was hectic, I was never in any ….. you know, I stayed at home, slept at home every night, did my work, did well in school, so you know he was so much stressed cause that`s what he said this was like almost, these were the things he kept saying to me, "I trusted you so much and I still can`t I can`t believe it you know he kept on saying that and everything and I understand like you know that your parents trust I think they had like too much trust on me you understand and I was kind like am example cause like I told you in my neighborhood they are a lot of things[yah] so I was kinda like you know not into that, I stayed at home didn`t have like ah a lot of friends and everything so yah. Overall when it comes to how it affected me with my friends and everything I think am not much really into that…. Safe to say I don't care anyway [yah] so it angered me at first cause who are they to judge [yah] but then was like that`s people but I was really worried on the effects it would have on my parents you understand [okay] and it was as I expected, actually I thought my father would be angry and kick me out and everything cause he a very, he`s well respected man, he`s people actually fear him you understand. As a result my mother found out I was pregnant I think it was 3 months pregnant but nobody was actually willing to tell my father because they were all afraid, so what we had to do was ask my mother`s sister cause my father respects people from akweni as you say it [yeah] so they had to ask her she's very old [akweni is] akweni is [in-laws] in-laws yes they had to ask his in-law like the eldest yah my grandmother they had to ask her transport her from her house to my house so that she could be the one like [to break the news] cause they were all scared, all the men, everybody knew but it was just him he was the only one in the dark that's how deep it was everyone was like scared even went you know this thing when a girl is pregnant they have to take her to the house like the boys house (………….) so when they asked (…………..) what does the father want as to like ihlaulo and everything [what`s that] (laughs) it's a, I don't know for other cultures but for us Zulu because [that`s like damage fee] the damage fee you understand [okay] yes [we have that as well] okay so they were asking the damage fee and we could not answer them you understand because our the father was still in the dark and they asked like okay when are you willing to tell him, they were like "we

don’t know, we are all afraid’’ they even asked my mother you are his wife, she was like “you don’t know that man “ you understand so finally we broke it down but I think he`s reaction shocked us all we expected, rage, anger, “get out of my house” you understand instead he was so hurt that he could not even hold it cause I was not there when they first told him, they told him on a Sunday, so he just cried in front of everybody, he was angry, my mother told me he was very angry, then he demanded that I came back from wherever I was hiding so I did come back and he talked to me he was more crushed than angry you understand I would have preferred anger [yeah] you know but seeing your parent cry because of you [most be hard]

Interviewer: So how did that make you feel seeing your father in that state?

Interviewee: I cried for life every day after that I cried, we were like so close like I said I had tomboyish background so am like his son you understand so we were like that close we watched wrestling together and everything so for him to not be able to be in the same room as me because he was so angry he can`t stand to like even look at me not because he hates me or anything but you can see the pain in his eyes that really, it was really tough I could not even study I had exams coming up in the next four days and I could not study. I would take my book, it was politics, I would take my book and just start crying you know just the thoughts cause I always would know that he comes back at home about half past 5, so every time its half past five I have to disappear and not be in sight just for him to be free, for him to be able to ask, cause my father loves television you would always find him in front of the tv but after that it was not like that came back straight from work did not even eat, when he found out I was pregnant he did not eat for a week, he did not eat, it was that bad so and of cause I had to blame myself cause I was to blame you understand.

Interviewer: What effect did it have on your feelings your emotions towards your pregnancy looking at your father`s reaction? How did it make you feel about your pregnancy?

Interviewee: Regret, I started regretting end everything as much as I had bonded with the baby and I had accepted it myself but seeing the effect it had on the most important man in my life made me like made me like think back for a second, I was a bit dreaming. I started even blaming the guy like he raped me or anything we had sex but (laughs) you know I started blaming him, “ life if I never met this idiot this wouldn`t have happened “ you understand started having those

regrets most of all and I thought like uhm for a dumb moment I was like, “maybe I should`ve listened to those dumb girls and actually aborted “ but then I actually said no that’s not me I can`t, I can`t do that.

Interviewer: I just want to know what was behind your decision to keep your pregnancy is it religious valves or is it personal? What made you not think about abortion at all?

Interviewee: I think all of those because for me, I always sound judgmental when I say things like this [yah]. For me, well murder … I think its murder I don’t even call it abortion, murder is murder whether we can now see the child or we can`t see the child I could never live with that knowing I murdered my own child and in like my family am not the first girl to fall pregnant when  they still starting am not the first one so I have seen the difficulties and everything I had a lot of things that could have made me like no I can`t do this but I think it was also I am a church girl and I believe that everything even though its sin is regarded as a mistake but I don`t believe any child is a mistake rather a blessing, you have to look at many things, the reason you go to church people come with their problems and get prayed for  a lot of women can`t have children and I look at if God did not just say that(sulks) just because you are having sex like a billion girls are having sex out there and just because you are having sex let just teach you a lesson “I don’t think it happens like that, I think there is a purpose, am not in depth (laughs) when it comes to you know but I think there was a purpose and could never see myself killing not just any child but my own you understand [yah] and it’s like there is actually no reason for me to cause I think ….. at some point but now I don’t the only reason or ……. Understandable reason for abortion for me it’s not understandable but you could learn to forgive a person like that is if they were raped by gangsters and everything and they don`t know the father, you could understand the emotional background but for me I was in love, we had sex without a condom, it’s not like even had the condom did not break, I had unprotected sex it was all on me you understand so the child had nothing to do with it so it was something like that and of course the father of the child was very supportive about the whole thing.

Interviewer: What was it like when you first told him when he found out you were pregnant?

Interviewee: Well I was going to deregister at UKZN you know so I just took the pregnancy test and I just did it at the toilet like it’s cool and when I saw those two lines everything paused, life

stopped like right there. He kept calling because he knew I was gonna do the pregnancy test in the morning, I was supposed to do it at home. He kept calling and I kept ignoring his calls and then I answered it and then I said we are pregnant and then I switched it off at the same time and then he kept trying to call and then he resorted to going to WhatsApp, he was so excited you would have sworn it was I don't know ………. I think also its because of his background that he was so he comes from a very hectic background so for him it was now he has a reason to go on so he was very happy about the baby it was like you gave him Christmas at the beginning of the year (laughs) he was more excited I was still shocked and in fear and angry at myself but yeah he was like "thank for bringing my life, for binging me a blessing he even said it was a boy but we still don't know the gender he was so excited [wow]

Interviewer: And his been supportive?

Interviewee: His very, very supportive.

Interviewer: So, what are some of the challenges you have faced in your pregnancy from the onset? Ah! From school to home to society ….to your health what are some of the things that have been difficult for you in the last 7 months?

Interviewee: Ahm, firstly was having to hide it from my father at first so having to hide it whenever he`s around, I have to suck it in which is bad for my baby you understand [yeah] and at first I started clinic very late I was going for 5 months and the Nurses were very angry I think it was also because I was 20 now you understand and so yah … and regarding society they were really like no challenges because most of them didn`t know, I don't not gonna say get along but am not very [and at school] at school I was not affected at all [lets just pause, okay so you were talking about the clinic] yeah the clinic, I went to my first scan when, I was about two months pregnant, that's 8 weeks so when I went to my second scan I was 4 months pregnant and we found out that the baby was too small, under developed so I couldn`t understand anything

Interviewer: What could have been the reason for that, what did the doctors say?

Interviewee: After I fell pregnant, I did not eat

Interviewer: What where the reasons for that?

Interviewee: I don’t know, maybe it was the pregnancy but am not gonna say [was it the pregnancy or was if the shock what was causing you, I know when people are pregnant they lose their appetite, they throw up but what was the reason for you not eating]

Interviewee: After I fell pregnant, I did not eat. I don’t usually eat. I didn’t know that when you fall pregnant you have to change your lifestyle and do things in a certain way. It was a shock when I went for a scan when I was five months pregnant and they said my baby looks like he’s two or three months, he was underdeveloped. So, the doctor said “it’s either your child is gonna be disabled or when you give birth, you’re gonna have to stay in hospital for a long time cause he’s really small, its either your child was gonna be disabled or after you give birth you gonna have to stay in hospital for a long time so I don’t think that’s good because you are a student, cause am gonna give birth in September so we write our exams by November so I need to try and end and also my blood was weak when I fell pregnant so I had weak blood so it meant, oh it affected me in school since I had a weak blood it means I was always tired so I always wanted to sleep so I did not find much time to study. If I walked in the house I had to find the bed like now you understand [yah] and I slept long hours not just which was not good for me, or the baby, resting in good [but not] that long so those were the challenges with the pregnancy and I started losing weight ah [instead of gaining weight] it’s like even now am bigger than this [really] but ever since I fell pregnant I have been which worries my mom a lot.

Interviewer: And your emotions, how`s your mental health?

Interviewee: My menta health [do you feel you think too much]. I do think too much even when it comes to the father of the child, I over exaggerate everything that he does, I always think there is a reason that he`s doing this now you know there is never like a good enough explanation from him now that am pregnant am always thinking but okay how could you do this to me knowing very well that am in this state you know I think I know am like that and I know I do that but am also in denial cause I always say I don’t use my pregnancy for anything I don’t we my pregnancy but I feel like ….. by falling pregnant I lost the boyfriend and actually gained the baby daddy cause now our relationship is now based on [the baby] are you okay? Is the child fine and everything you know there is like literally no time for me and him, there is no time, we go to the same school he`s doing he`s third year here but we would go like two weeks without seeing each other just chatting on WhatsApp and for me it’s like not normal that`s like not us, but as long as

he asks about the baby to him it's like all good [and to you?] its horrible cause like I feel neglected in a way, I feel like he`s neglecting me, not the child but me and of cause I have to look at it as if it's not happening cause am pregnant and everything and I know that I should understand cause he`s going through a lot and he`s seeing a psychologist because he has problems at home, at school its affecting school and everything and now me with my stress and the pregnancy problems that our child is underdeveloped and everything it all adds to that so at times I feel like I don't have the rights to be as angry or as heartbroken as I am but I need to understand for him but I also feel like it's always about him and never about me you understand me.

Interviewer: Am just trying to clarify, so you feel his being there for the baby but he hasn`t been there for you as much? does that have an effect on how you feel about your pregnancy or about your relationship?

Interviewee: Our relationship yes

Interviewer: Does it make you feel my less supported?

Interviewer: Yes, it does. It really really does cause he`s willing to do anything for the baby but when it comes to me and him because I also told him, we talk about this, I told him that I would rather we call what we do friends that are about to have a child than keep claiming, a relationship, I can`t be lonely and be in a relationship. I can`t feel this way.

Interviewer: So, do you feel lonely?

Interviewee: Yah I do I feel very lonely and I think my loneliness I feel it helps me in a way because when I feel lonely it actually pushes me to study [okay] cause when it comes to his every academic achievements, he`s really smart, he`s studying civil engineering smart and everything so it pushes me you know I can`t be really mad at someone who has ……….. academically has it on point, it's all going well for him so I also need to push myself.

Interviewer: Okay so your loneliness makes you strive for greater things, what about your health do you feel your loneliness, you know when someone is pregnant they should be emotionally okay because everything that you feel neglected or when you feel lonely it somehow takes you to this place where you are feeling sad and depressed, do you ever feel like that?

Interviewee: I do a lot [and when you feel like that] I cry because people don’t understand how deep pregnancy is cause when you get to that state they call it hormones, they just call it hormones but I think us as pregnant women things like that happen we get pushed to this spot where you feel so hopeless and everything is just so now everything that we do or can`t wait for is just give birth and try to go with life and move on as it is.

Interviewer: Okay, so you have told me your pregnancy is 7 months

Interviewee: Am 6 going to 7 [okay]

Interviewer: At what stage, we talking about support, so your dad has been disappointed how supportive has he been?

Interviewee: He just out [we can`t talk about support when it comes with your dad] no he doesn`t even ask [it hasn`t sunk in for him yet] no it hasn`t [we`ll probably talk about that after the baby comes] (laughs)

Interviewer: And your mom?

Interviewee: she’s very very supportive, she`s my heroin [and your boyfriend has been supportive] yah

Interviewer: So, when you think about your pregnancy, you are in your 2nd trimester, but when you think about it from the beginning as you go on to the third trimester at what point do you think a pregnant woman needs more support or you own when you think about your pregnancy from the time you found out you were pregnant going to you know the delivery room when do you think, or after the baby comes, when do you think support is cardinal or at what stage?

Interviewee: I think support is very important when you first find out you pregnant. I think that`s the most cause I think if you don’t get [why do you say that] I think not being supported or being lonely at that time you actually find out its okay cause pregnancy is life changing so when you find out at that moment a lot of things are going through your mind like something we talked about earlier abortion you know you are like so confused, when I found out I was pregnant although he was supporting me and everything and I knew that he would be a great Father, he would be there for the baby and everything so with him my confidence with him was at 100%

but because I knew how badly it would affect my father, it made me think of a lot of things like. I could never abort but I can`t lie to you and say I didn`t think about it you understand when I thought about my father I can`t say I didn't think about things like. Ah cause when it got really deep when I was really thinking about it I thought of things like abortion, suicide a lot of things for like or long time especially you know when you have a…..it was so new to me when you guilty of something and the person you gonna hurt with that guilt doesn`t know, he`s in the dark every time you talk to them with smile or do something for me I would feel like I don't deserve it if only you knew what I had done or how I would hurt you so it was that. If it was like an accepted pregnancy it would have been different because everyone would have known so support I think like from the point on a girl finds out they pregnant, [that is] yeah that is, cause I have seen some (…………) happen cause they did not get enough support like I said many kill themselves many stay in denial after finding out they pregnant so when decide to make an action and like a friend of mine drunk paraffine so hoping trying kill her and the baby lucky they both survived but it was … you know cause I don't think she got, cause when she found out she was pregnant ….. the father was scared because he feared his father, she was scared because she had a lot of positions in church and everything, she was you know like me many looked up to me in a way especially when it comes to church and everything it was something that wasn`t expected of her …… they were both in a bad space, her and the [boyfriend] boyfriend so they both decided to take their lives [wow] and they both survived [wow] so I think [so okay support it's at the beginning] (laughs) and am not gonna speak much about after I give birth am sure I will also need support there [yeah] and also during pregnancy I don't think there is a time when am good I don't need any though I did, I pushed lot of people, lost many friends ……. Let me not say many because I don't have a lot, I lost a few friend`s (laughs) cause of my pregnancy

Interviewer: But what was causing you to push people away? Do you feel you had disappointed them or did you just want to be left alone or?

Interviewee: I wanted to be left alone to deal with this. I wanted to deal with everything on my own [okay] thought I could do it ah [would you do it on your own] (laughs) no I think women who go through their pregnancy alone are very strong, I think from now on after experiencing this I salute (laughs) and give much respect to people cause I don't know where I could be if I didn`t get may be even an ounce of support I don't know where I would be right now

Interviewer: So, does your boyfriend come with you to the scans, the clinics?

Interviewee: That's another problem ahm at first, he did but now he prefers if I go there and I tell him how everything is.

Interviewer: Why does he prefer like that these days?

Interviewee: Am not gonna say he prefers, it was a wrong word that's how it is these days [okay] cause we fight so much and for me I don't feel he gives, I know he loves his child but I don't think he gives it enough effort or shows me, like the problem is now with me, or shows me enough support you understand cause need him, it's like the last time we were supposed to go to the scan together. Ah I was gonna finish earlier he was gonna finish latter so we decided, fine I will just go early you don't have to come with me, but then I told him, okay am not gonna go as early, when it was the day he told me "ukuti" oh am done, are you guys done there, what did the doctor`s say am like I haven't been to the doctor am still at the gate in school am about to go there, I did not say it in many words okay I want you to come with me now but I expected him to say, oh okay you still here let me just come with you guys cause am also done "cause he was also done, oh but all he said was that "oh okay baby go well and I hope you and my baby are good and everything " it really hurt me but he doesn`t understand I don't think he understands how much he is needed like for now cause I always tell him one thing ukuti you don't become a father when the baby in my belly that's when you became a father cause for him at time we argue about things that are just dumb. He feels as if ahm I excuse him a lot call the child my child my baby and everything as if he`s not part of it you understand. I think why are you even mad I can notnot call it my child when I go to the scans, I go to the clinic. This whole thing first started when he had to write he`s paperahm it was the first visit we were supposed to go together and then he had to write that day all, and I took it well cause he had to write and because I was okay with if he thought it was something he had to do regularly so yah [so that was the beginning, so you do feel left out when he doesn`t come with you to those things] yah I do [okay]

Interviewer: Ah so where are you going to have your baby from? You`ll be home, you`ll be at home

Interviewee: Yah I will be at home [your parents, okay]

Interviewer: And after the baby is born in September you`ll have to come to school isn`t it?

Interviewee: Yes

Interviewer: So, who’s going to help you take care of the baby?

Interviewee: My mother [oh your mom, she doesn`t go to work] she doesn`t work [so she`ll stay at home and take care] she`s a stay at home mom [okay] like I said she`s very supportive she even said “ukuti” no it`s my first grandchild so it’s okay I will take care of the baby” she even wants me to start going to res now and I was like no when school opens next [semester] year [next year?] yeah when school opens my baby will be five months so I was like mom I can’t leave you with a five month old, I still have to breast feed cause you have to breastfeed for six months so she was like” no its okay, we can use, you can like pump the milk for me and everything as long as you go to school or you can come back on Monday I think for her… she has so much love and so many wishes for me, she`s just giving me the love and support she`s okay with taking care of the baby

Interviewer: So, who would you say has been your greatest source of support is it your mom your boyfriend

Interviewee: My mom [your mom, okay]

Interviewer: And what does that mean to you?

Interviewee: (laughs) everything I wanna buy earth for that woman

Interviewer: Okay so when the baby comes, before I come to the baby coming, when you look at all the types of support that a person can receive, a woman can receive when they are pregnant what do you think is the most important support to you? When you look at emotional support, moral support, financial support, supports coming to clinics and taking care of the baby after the baby is born, what do you think is the most important support to you? What would mean the most to you in terms of support at this point? What is the most important type of support?

Interviewee: A person who is pregnant just needs support, love - no matter how bad the situation is or how it has happened. Having people to talk to and that understand, are really there for you, lending their ear more than their mouth, people to talk to [okay]

Interviewer: So, after the baby comes what will be the most important type of support to you/ do you still feel you`ll need understanding people to talk to and that won't judge you

Interviewee: No (laughs) I think it's finding a way to judge both school and my baby,

Interviewer: So what kind of support do you think you`ll need?

Interviewee: Will need emotional supporting (laughs) [what do you need] the other thing I thing will be my baby`s everything, I think if my baby`s fine and school is going well I think if my baby`s fine and school is going well I think for me that will be it, I can deal with the rest, I can handle the rest, I don`t even see me and my boyfriend after the baby (laughs)

Interviewer: Okay so when you think about the question has run away, you said something then I wanted to follow up uh support after your baby, your mom will be home to take care of the baby so you won`t need a nanny or anything.

Interviewee: No [okay]

Interviewer: So what role is your boyfriend going to play then is he going to financially the child or its going to be your parent's responsibility? Who`s going to finance the child, the clothing, diapers, the milk?

Interviewee: He`ll play a major part but also from my family they have a mentality of teaching us not to depend on the men or the fathers of our children, they already helping out my grandmother is buying clothes for the  baby  is not even there (laughs) but also from him even now he`s supposed to be financing cause he`s saving up money I think about like a month ago, I think it was  April he gave me R 5000 to save [wow] he said it was far, I asked him what it was for he okay I then actually your body`s gonna change, and you gonna gain a lot of weight and there is a lot of cravings that you were going to have so that should just, I think it should cover up for you know all those things. Please do save if you can cause we don't know when the baby`s gonna pop out, so I said okay I think he`s gonna continue to do that.

Interviewer: And how did it make you feel to see that he was financing the needs?
Interviewee: It was a shock for me [you didn`t expect that] I did not expect if you know cause when he gave me like a 100 rands so what are you crying for, you buy it you know and

everything gave me a 100 rand so when he said I wanna give you some money for everything I wasn`t expecting it to be like 5000 or something I was thinking maybe four hundred rand or five hundred rand cause for me I don't like a lot of things I don't crave actually, I don't crave for anything , I don't I am like that so it was too much.

Interviewer: That was financial support was it, would you consider that to be as important as emotional support?

Interviewee: No, [it doesn`t match up] no [which one would you rather have] emotional support

Interviewer: So, you have told me your partner has been very involved in spite of him not coming to clinics with you and scans but he`s been very concerned about your well-being and the baby`s well-being, so what do you think generally when we talk about partner support during pregnancy? Do you think it is something important?

Interviewee: Support, yes, it is very important cause I think if you don't receive support and feel like neglected as if the baby daddy does not want the child …. How its gonna make you feel will obviously affect the baby so I think partner support is like really important it's like one of the core supports whatever that you need it is very important. [okay]

Interviewer: What does it mean to you to have your partner`s support, what does it mean to you your partner`s support in all the ways that he does?

Interviewee: It means everything, it means am not alone in this, it shows that this is our child, it takes away a lot of negativity that I would feel, and I think in a way am a bit lucky although sometimes I think he is a jerk (laughs) but I think am very lucky to have a partner I know who`ll make a great father or will be there for us, am not worried when it comes to my child house we`ve even talked about me giving birth and the child giving at his house and everything so when it really comes to the pregnancy and my child am …. Its everything.

Interviewer: Does the involvement of the father influence all the decisions in the pregnancy, do you run everything by him do you thing independently? Does having him around influence things that you do?

Interviewee: Yes, it does. I remember when he first found out I was pregnant and I think I was still in denial I did something very stupid I drank alcohol and he was so angry that he couldn`t hold it anymore he cried, like he really cried, like ‘do you really wanna kill my child” like that you know and I understood that. Me being selfish this is not my child it’s our child everything should involve him when it comes to my pregnancy and everything and we talk about everything even when am about to start doing something even if he wants me to do something regarding my pregnancy he talks to me first be it name of the child if there is a pill that I have to take he needs to see if it’s gonna be good so everything you know has to go through him at the end of the day life has no guarantees, we love our child but we need to face reality that a lot of women fall pregnant but don’t have the opportunity to hold their babies cause some don’t make it, so me being selfish right now and if anything happened I think I would have to take the blame all by myself but if I know he was part of everything he knew everything I think it would help a lot.

Interviewer: We haven`t talked much about your school how has it been being pregnant, coming to school, how are your grades do you find time to study how do you cope?

Interviewee: Well I do find time to study and I find time to study and the grades are good am gonna lie[so the pregnancy has not affected school work it’s just walking around school is the problem now all the stars, you sway they see I don’t know what (laughs) [how does it make you feel] it angers me you know they stars to a point were am like should I stop to let you take a picture then they just walk away, I think it’s that those stores and everything and it’s like so hypocritical in a way because if I am walking alone or with other girls I get those stares like(sulks), “that dumb bitch” you understand but if am walking with a, cause my baby daddy goes to the same school, it’s all sweet now you understand, there the stare types kinda like change they not like (sulks) “ you get pregnant and you still studying all the way from that to “oh it’s so sweet they love each other and they going to have a baby” and everything I think that’s what pisses me off (laughs) but when it comes to school work and everything I make sure, I try, I push myself when it comes to school [okay]

Interviewer: So, do you think, you, you have talked about male partner support being very important in pregnancy okay, do you actually think there is a relationship between the health of the mother and her baby and the support they receive from the partner.

Interviewee: Yes, cause like you said anything I feel affects my baby so if am stressed it`s gonna hurt or affect my baby negatively, so if so if I, if because of my partner I feel certain feelings which are not good for my child you understand then that's where it all goes wrong cause stress does a lot of things to people you know in most cases people lose weight, the eating Baptists change so if stress can do things like that I think we see the importance of the child regarding the health of the mother and child.

Interviewer: Thank you so much it`s been such as a pleasure talking to you.

Interview 6

Interviewer: Thank you for taking part in this study, can we just talk about your pregnancy experience, what has been your experience being pregnant? The last 25 weeks you know going to school and just dealing with everything.

Interviewee: Okay I think I would say for the first few months like it was a problem because you tend to get sleepy, tired a lot and want to sleep and yah but that`s what you normally have okay the other thing you need like you know when you don't have a good support structure things tend to get hectic or difficult for you but if you have a good support structure literally things tend to get easier because you know you have someone that will help you with this and what not, not family alone even with friends if they support you. They able to guide you on what to do even when you are not feeling well because If you not feeling well they'll tell you use this use that because I remember I used there was this day I had been, I didn't know what it is, I called my friend`s I got pregnant and then explained that oh my boyfriend told me its heart burn but he didn`t know what to do the only thing he told me about was that Gaviscon, didn't have Gaviscon in the house then I called my friend he told me to mix milk sugar all those things that could help and then yah I think I remember that was if there was this other day, where I remember …… okay I first went to the hospital in April, first time going to the clinic ….. the experience wasn`t okay I didn't like it, seeing that whenever I see something is wrong I tell you the person is not right and what not what not …….so besides the experience and what not on Tuesday I went to the hospital and got a bad experience and the following …. I think 3 days I had abdomen pains I didn't know what was happening I was so scared. I went to the hospital because I didn't have money mainly I go to see a doctor …. Ah yah I didn't have money and then I got there, I had to wait for like 4 hours. I had pains I had to wait from 8 until 12 for the doctor to come, so when the doctor came they didn't know what was wrong with me but they suspected that she recommended me to thingy, what's this one. A surgeon for an appendicitis they need to check for like for my appendix went there it was a mad experience took me to an ultra-sound, didn't see my appendix and what not went to the doctor and admitted me to the hospital like when I was walking to and from to and from I had pains like so I couldn't feel the baby in my womb so I thought may be my baby had died and what not I was crying the whole day like leaving from my room to the hospital I was crying complaining

Interviewer: And who was there for you during that period?

Interviewee: Ahm [where you just alone at the hospital], yah I was alone at the hospital I was irritated at the fact that my boyfriend did not wanna go with me like he had to go and his teaching practices so I thought that may be on that day because we like I had to go to the clinic he`ll go with me because am not feeling well but he didn't so, from here in this room I was complaining to the clinic and from there and I didn't have anyone except for …. Friends yah except from my friends and then yah latter on …. Latter left at the thingy because okay the reason why I suspected the appendicitis was that …… but it was kinda like ticklish because you cannot say if you ask me that I have how many times to I go to the 100 to per And if I say that I have been doing that you can`t say its appendicitis or something because there is nothing there but to with them they thought it was appendicitis the doctor said that it could be that the appendix because they cannot see it the ultra-sound it could be that its compressed underneath my eggs or its behind the womb or something so that could be the reason and them that`s why I had to be admitted in hospital she said but if I do respond to the anti-biotics I am not gonna go for an operation but if I don't I will go for an operation and lose my baby so that was a bad experience so but I told myself that am not going to do it, am not gonna have an operation even if it means like during my pregnancy I am gonna have the experience its fine and yeah that way that and then yah.

Interviewer: How did it make you feel having to go through that whole experience at the hospital by yourself?

Interviewee: Yoh …. I think am used to going through experiences alone and like being there for myself so it was not that much but I expected the baby`s father to be there with me because like the first time we found out that I was pregnant he said "we are in this together" so I thought like we in this together when he likes but normally I feel like am in this alone.

Interviewer: So, did you feel like he neglected you?

Interviewee: Yah I felt like that, I felt like he neglected me

Interviewer: And how did that make you feel?

Interviewee: (laughs) was so irritated and yah I was crying like a child I felt so sad and I felt you know I wish I was not pregnant and at the same time alike I love you baby but yah (laughs) at this point in time I wish because at first in the morning I felt I as having cramps and what not so yah

Interviewer: So apart from your experience ahm what do you think, or before you got pregnant what would come to your mind when you thought about pregnancy? What did you think pregnancy was like and comparing it your situation what is different?

Interviewee: (laughs) okay with me I didn't want to get pregnant while am still at school because I thought that when you pregnant …. You need to be tendered when you pregnant, have seen with other people with my friends normally when they are pregnant, like pregnant person there are always tired, they always sleepy, they always wanna sleep they always complaining you know about so many things they hungry they this they that, gain weight, gain thingy what not, so for me I thought it's something that should b;;e like you should get pregnant when you are working and what not. [okay] and then it happened that I was not working so you know when like having to study for a test and am pregnant in my mind I think I had that thing because whatever you thinking comes to being, so in my mind I was like you know what am not supposed to be studying am not supposed to be in school like this thing is supposed to be happening while am working so that I will have so much time to relax and spend with my baby you know so I felt yah its wrong but it's something that one has to come around with and just get used to it because it has happened and just look for a way forward

Interviewer: Tell me about how you found out, what you felt, how was it?

Interviewee: When I first, okay, I suspect [first of all was it intended]. Okay the baby`s father says he intended to happen [okay] but I did not want and it bothers me had so many plans for this year like to take out myself to be independent as usual but when I was at home I think in December I don't know there was something in my mind like there is, okay I kept on dreaming that I am pregnant, yah I dreamt that I was pregnant, then I wood dream that am having a baby, like you know it was all of those things than during the day I would feel cold and then I would just say to my gran, "am feeling cold I just want to go and lay down and am tired"….. and I started telling the baby`s father that I think am pregnant and then I think after a few weeks, but I

thought I was joking to him when I said I think am pregnant, only to find out that when we went to some clinic in town that I was pregnant. I was so irritated

Interviewer: What was the first thing that came to your mind? (laughs)

Interviewee: It was like okay my parents, like all the plans that I had for this year they were just crushed like [plans like] I like going to gym I like exercising so I wanted to be in shape, go to gym have fun with my friends, go to work, [and school, how far are you in school] am doing my second year [okay] so I was like am just gonna, it's gonna be me, my books and friends you know all those things and then I was like eish (…….) things happen there is nothing you can do but I was so irritated when I did the test then I had to show it to the nurse in that clinic.

Interviewer: If you don't mind me asking your age?

Interviewee: Am 25

Interviewer: Okay so tell me about the home front, how was it when you told your parents that you pregnant?

Interviewee: (laughs loudly) I haven't told them [you haven`t told them?] (laughs) yeah [they don't know you are pregnant] I think that I was gonna tell them today (laughs)

Interviewer: when where you last home?

Interviewee: during the Easter [and they didn`t notice] they didn't see it [wow] my stomach started showing after 5 months [okay]

Interviewer: so, when you think about it telling them, breaking the news to them, what are you expectations?

Interviewee: (laughs) okay with my mom we`ve been talking about that when I was home, ahm she was busy telling me about okay she has a friend who`s pregnant right now but she`s younger than her so the friend of hers she`s engaged everything is very well she`s engaged, she`s gonna be getting married soon, the baby`s father is here, she was so excited that and she`s 27 and it's her first baby so she was like, "oh wow like I wish for my kids the same thing could happen" and then later on she would change and be like you know because my other sisters, am the only

one at home who doesn`t have a baby, so she would be like you know with me I wanted you guys, I wanted your sister, I wanted all of you to go to school finish and after finishing school and then you could have babies, you can do whatever afterwards, but now since with your other sisters it didn`t happen like that it's okay but, that's why whenever my sisters get pregnant my mother would not talk to them and would not take care of the baby when the baby`s young, she`ll may come around after the aby is a year old and then she`ll be okay with you but before that she`s not gonna be involved so you know all that and she`s been telling me about a friend who are still virgins and not and are still in their thirties, three late thirties so all those things and then on Sunday I was talking to her oh I have been invited to she told me I have been invited to be a speaker in umemulo, I don't know if whether you know umemulo, it's like the 21st but a traditional 21st , yeah I have been invited to that because she once did that for me a swell when I was 21 so this girl was invited to my umemulo ….. and then she wants me to be the speaker.

Interviewer: And what was your response?

Interviewee: (laughs) am like I will think about it, we`ll see, because I said to her am not coming home in June I will be working [so you want be working] I won`t be working, it was just a way of getting, of not going home [okay] excuse actually so then on Sunday she called have you decided because people they wanna know, and I was like no will tell you and then she started telling me about a friend`s daughter who gave birth to a baby girl and then she`s like you know, " I don't understand this kid, how can you have a child when you not working because she`s not working who does that and what not am like no you can`t judge people because things happen and mistakes do happen you know and she`s like, " so those mistakes do happen to you as well?" no am not saying they`ll happen to me or it's like I am pregnant but am just saying don't judge another person because anything can happen even with me you don't say am an exception to all thing but even me I do mistakes we learn from those mistakes you know, but I will tell her because but ummmm she was like if you pregnant how are you going to take care of the baby and whose gonna support you and what not and school because my other sister she gave birth to her baby and then she never went back to school so I wanted to tell her that because with me okay the baby`s father`s family know that am pregnant, its only my family that does not know so we plan that after giving birth am gonna take the baby to the father`s family so that, cause he`s staying here in Maritzburg so it`s gonna be easy for me to travel every now and then, I wanna

assure that irrespective of me getting pregnant and having a baby am still going to be the same person except that I will have a baby and with school am still gonna be continuing and still be the same person she knew and what not.

Interviewer: so, when you think about your mother`s reaction do you think she`ll be supportive or she`ll treat you the same way she treated your sisters?

Interviewee: Ooooooooooooh ah I know that she`s not gonna be supportive, she`s just gonna shout me, because I didn’t wanna tell her before exams because I didn’t wanna get all emotional and what not, I didn’t want to be sad [okay] so am just gonna tell her after my last paper, okay this is what happened, but am gonna tell my granny first then her later

Interviewer: So …. considering that you this far gone you are 25 weeks your boyfriend he`s been supportive?

Interviewee: You know when you are pregnant, I think you expect a lot when you know that this person is the baby’s father, it’s like you want them to do more. You want them to try anyway and compensate for doing what they did to you, because if it wasn’t for him I actually wouldn’t be pregnant so he has to be there for me. “but there are days where am like you know what I wish I was one of those girls who didn’t have their men next to me because he`s kind like he`s straight, there are things that I need to do, there is a way am supposed to dress, like he has too many rules, it’s like he`s the one whose pregnant, even when am sleeping, am not allowed to okay not now even before like now am not allowed to sleep out with my stomach even on top of him, am not allowed to lay on top of him because he`s gona be like no you hurting the baby I am not allowed to touch my baby press my baby like this he gona be like, "no you hurting the baby don’t do that so he has too many rules.

Interviewer: So, does that cause an environment that you feel he`s not supportive?

Interviewee: okay not that he`s supportive but I think he`s too much [okay] like in cases where like with me you know when am going for antenatal I wanted that my mom should be next to me, we should be in this together he said but when I had to go to the clinic he`s not gonna be there, or sometimes he`ll be here sleeping and I have to do my work when I ask him to do something like can you please get me water, Sometimes he’ll be sleeping [when he visits her in

her campus room] and I have to do my schoolwork then when I ask him to do something like can you please get me some water, sometimes he's gonna be like he's tired or when I want him to do something for me he's gonna complain.

Interviewer: So, do you feel he hasn`t been supportive enough, or he could do better?

Interviewer: partly

Interviewer:(laughs) okay on a scale of I to 10 how would you rate his support?

Interviewee: half, 5, no 6 yes six

Interviewer: (laughs) so you mentioned you would have loved or him to come to doctor`s appointments

Interviewee: no doctor`s appointments he goes cause he has to pay [okay] there was this time when I had to go to the scan and he did not come it was so rude, that was after I was admitted to hospital because we wanted to check what was wrong, we wanted to find out what was wrong and I told him that he has to come with me because even when we go to the doctor he comes with me but he just gave me the money and went to work, I had to go there alone, I had to look for a gynae myself because the gynae that I was using like I wanted to get another opinion so he was not there I felt like he`s not been supportive, I felt like, okay with him he`s there for me whenever he wants and whenever I want him to be there for me, he`s not there for me.

Interviewer: how does that make you feel?

Interviewee: (sulks) its irritating …. It's irritating but you know (laughs) learn to understand these things

Interviewer: So, he hasn`t come with you to antenatal?

Interviewee: No, but there is this other time last month, he said okay we were supposed to go together in the morning and then he didn`t go with me and then he said he`s gonna meet us there, thinking we go spend more hours sitting there but only to find out that I spent less than an hour, he got there like I had already left when he got there he started texting me asking my where abouts I ignored him then may be 30 minutes later I reported like I have already left am on

campus already and then he was so irritated and I expected him to say something but inside I was like yah it serves you right next time you`ll come with me.

Interviewer: So, you have said you are 25 weeks gone, at what stage during your pregnancy do you feel you need the most support, when you think about the last 25 weeks and when you think of the next 15 weeks or so, at what stage do you feel you`ll need the most support or at what stage do you feel you needed the most support, is it going trimester by trimester, was it at the beginning, is it now in the middle, do you think in the future you`ll need more support, at what stage is support most important to you both from your family, friends as well as from your partner?

Interviewee: I think it will be [you know people will have different needs some people will say I want support at the beginning because am trying to accept this and just assimilate everything and move on with my life, some people will say no it's at the end because am tired, I need money and the baby`s coming, [what about you personally, on your own] Throughout the pregnancy you need support because there are times where you feel like I wish I could just give up everything like you know, school cause sometimes you find it frustrating, and you don't wanna deal with it. I think you need that pillar of strength every day; someone that will be calling you and asking you 'How are you doing? How's everything? How's the baby?' Like I think every day during pregnancy even after pregnancy. How's the baby" like I think every day [everytime] during the pregnancy even after pregnancy like you know you`ll become too emotional over small little things when you pregnant so I think every day.

Interviewer: So whose been your greatest source of support?

Interviewee: ahm (laughs) hey its different people [I know you can`t say your family since they don't know yet but whose been your biggest] (laughs) there is no greater because okay I have different friends, okay the ones here on campus they have been good yeah they have been good, then I have another one like you know when she calls me it's like "oh my gosh she feels the gap that was opened, she just, talks to me, advises me you know with things and what not, things to look forward to, so yah so many people.

Interviewer: and is there anything the people around you can do to increase, is there anything you long for, any type of support you long for that is not being given or what can people around you do to make you feel more supported or to seem more supportive in your eyes?

Interviewee: okay (laughs) I wouldn`t say people I think the only person would be him, like the baby`s father, I think he`s the one whose supposed to be like doing more, like supporting me more like my friends, because my friends, it's not like okay there are my friends but it's not like it's there baby or something or they have any attachments it's just they have attachments with me not with the baby even though they do support now and then but I think the person whose supposed to be doing more it`s him

Interviewer: Uhm so where are you going to have the baby from?

Interviewee: where am I gonna have it from? [yah, where is home]. Newcastle, I am gonna have it here, I don't want my baby to travel, and I don't want to have my baby in Durban, I don't want my baby to travel from here to Maritzburg, okay right now [you are not decided] yah am not decided but am a bit skeptical of taking my baby to stay with the baby`s father.

Interviewer: So, your baby will be born during the semester, right?

Interviewee: Yeah next semester in September

Interviewee: So how are you going to juggle everything, will you spend a few days with your baby or will you immediately take the baby?

Interviewee: No, like I wanna spend time with my baby because, okay am still undecided I wanna breast feed, but now next semester I wanted to move to Maritzburg but he said no [you can move to Maritzburg, school and everything] yeah I wanted to transfer to Maritzburg campus but he said no I don't know for what reason but he had his own reasons because I want to be next to my baby, I don't wanna be travelling according to him he wants me to travel but I don't wanna travel because it will be too much for me with the pains and what not so I don't wanna be doing much then I decided that in September because they say that am due on the 21st the school is gonna be closed, because we close on the 19th but then that week of the 19th am not gonna be around by then am gonna be in Maritzburg like preparing for birth and what not.

Interviewer: So as at now you planning on delivering in Maritzburg

Interviewee: Yeah

Interviewer: And then leave the baby there?

Interviewee: Yeah [and come back to school] yeah but I think I will stay because, but am still undecided maybe I will transfer to Maritzburg and I have thingy it I want to stay with my baby, find a place and stay here in Durban could need a nanny and what not but will see.

Interviewer: So, who’s going to help you during that time taking care of the Baby?

Interviewee: Okay it’s gonna be the granny and nanny okay I think the whole family from the father`s side I think they`ll help

Interviewer: Do you feel your family would also want to help?

Interviewee: I think they would want to help, okay the thing is, I think they not gonna be happy that my baby is staying with the father`s family.

Interviewer: What would they prefer? You have told me your Mother doesn`t usually help the first instant.

Interviewee: yeah but they would prefer the baby stays with them okay but at the same time they would prefer the baby stays with me like I have to be around you know and take care of my baby but then I think

Interviewer: So, your parents would want you to play that present role where you are present with the baby but them not being directly taking care of the Baby?

Interviewee: Okay they want me to be around and then they`ll be helping me or okay if they understand that okay I have to go back to school they`ll take care of the baby but then I don’t want that I wanna be there for my baby, I wanna be there for every step, I wanna see every step

Interviewer: And financially, financial issues, who’s going to be doing things when the baby comes?

Interviewee: (Laughs) that one it's the baby`s father [okay entirely?] No! not entirely …. Okay I am also gonna be taking part because am planning that in July we need to go and buy the baby a few stuff cosmetics and what not. I wanna buy them in bulk so that they will last longer so that in the following month will be able to focus on other things.

Interviewer: We have talked about support during pregnancy, what about after the baby, when do you feel more support is needed is it before on the (……) or before I ask about now or later right now what`s the most important support to you?

Interviewee: like even someone to talk to, okay with friends I need someone to talk to and with the baby`s father having someone, you know when I need a massage I need to get a massage(laughs) because we in this together right so that massage I have to get it and in times you get to have cravings so he has to come around with that as well and take part (laughs)

Interviewer: So, we are also talking about financial support, we are also talking about emotional support but which one comes first to you? What would you rather have?

Interviewee: Emotional support [emotional support]

Interviewer: What about after the baby comes, what's going to be important?

Interviewee: Okay with me after the baby comes am not gonna be needing much support [okay] because am gonna be back to my old self …………..

Interviewer: Do you feel you`ll not need much support then?

Interviewee: Maybe like having friends to talk to, okay the most support that I would appreciate would be for my baby not with me [okay, so someone to help take care] like am not really sure I won`t be around so I don't know they`ll treat my baby so yah

Interviewer: Okay so you have talked about who'sgoing to help you then just generally we have talked about support from family friends, your partner. What do you think about male partner support during pregnancy, what is your perception

Interviewee: Okay I think a man should be there for you because during conception you are together so I think the whole process the pregnancy, they should be there that is very important

because okay my friends have been telling me that I should have more sex of which I don't think I like that but I think it helps a well with the muscles and what not …. But I think the baby`s father`s has to be there for you because at least with them you can complain do whatever even though sometimes it gets too much for them and you tend to fight a lot and sometimes (laughs) you might break up over that but you have to understand because with me I don't know what's happening but I think it's the hormones sometimes I would be shouting at him, the next minute am like no it's the hormones it's not me you just have to understand it's the baby, when I come back to my senses I just say to him it`s the baby and what not. But we fight a lot when we together but then he kinda like has to understands because you pregnant you not normal.

Interviewer: From the support needs that we can talk about emotional support, financial support, moral support which one do you think your partner has taken up the most? What is the doing? The most?

Interviewee: I don't know hey, I think may be, you to feel I know from this thing that I hate when I was growing up I think I expected too much that`s why when he`s going something I don't really see it, I see it later because of my expectations and I think it's wrong for one to have to expect them to play a role as the father I think emotional support [and financially how has it been] and financially wise [have you been financially supported so far or do you find yourself doing most] the thing is that am independent I like doing things by myself, I don't like having to ask for a mam to give me money or do something, it's only now you know I just want to feel I just wanna know how It feels for a woman to ask for money but then when I want something even if am craving I go to buy it myself or I tell him here`s the card go I need this [okay]

Interviewer: So, you have told me he`s been emotionally supportive, what does that mean to you to have your baby`s father support you emotionally?

Interviewee: (Laughs) okay like I said to me, okay to me with my expectations so sometimes I think he`s not doing much you see so oh okay I feel so lucky and I feel so blessed that I have such a man (Laughs) and there are days where I am like you know what I wish I didn't have such a man like in most cases am like happy because he`s sometimes considerate, okay I will say he`s considerate and yah he`s considerate sometimes I would expect him to talk to the baby, he wouldn't want to talk to the baby like no am not ready like why are you not ready or sometimes

let’s just play with the baby or something and then yah play as much as his being like emotionally supportive but I get irritated with his rules because it’s more like he`s the one whose pregnant.

Interviewer: why do you think he has all those rules?

Interviewee: Okay I think, the other thing is that his ben doing some research, even with my diet his doing some research and then he goes and buys those things and then he will later tell me this how you need to do things and what not. I think his being pre-cautions so I understand in a way I think it’s too much you can`t expect me likeI was 15 weeks or pregnant then you are telling me that I can`t sleep with my tummy like what’s that now I understand you can say whatever but before that because I used to tell him there will come a time when I won`t be able to sleep with my tummy so yah.

Interviewer: how does the involvement of your partner influence the decisions you make in your pregnancy?

Interviewer: I think I don’t make most decisions like he`s the one making most decisions.

Interviewer: how does that make you feel?

Interviewee: I don’t like it but I think I have come to kinda like understand but I don’t like it Because it’s my baby, it’s more like his hijacking me because he was telling me that okay traditionally the wanna is not supposed to be naming the baby it’s the baby`s grandfather from the father`s side and the father who I would say have the right or something to name the baby. I think that’s not right it’s my baby and like with most things it’s more like his the one who wants to take decisions or he is the one who wants to make decisions for the baby well it’s my baby am the one whose carrying the baby so I think in a way am being side lined because now I feel like once the baby is born his not gonna have time for me it`s just gonna be him and the baby (laugh), but he`s been saying that it’s not gonna be like that but I feel like even with his family it’s just gonna be them and the baby because I think that the mother is just gonna hijack my baby from me and I don’t want that. I want my baby to know that (laughs) I am the mother me alone no one else but yah at the same time I think am excited for my baby that he`s gonna have more support but at the same time I feel sorry for myself [because you feel like you`ll be in the

background] yeah I think so but I goes I just have to leave with it but I told him that after two years am taking my baby and I can`t have my baby being taking care by another woman.

Interviewer: would you completed, you would have completed your studies than?

Interviewee: yeah and irrespective of that I have to take my baby, you know my baby has to go to school, not have a nanny look after my baby I don't know what she does or whatever because the granny is old and the granny cannot take care of the baby he was like no there will be a nanny around and then, inter on his mother will be back from work "so it will be better, i don't like someone taking care of my baby because i don't know what they doing, i have had various experiences with nanny's with my friend so I don't want that so after my baby is two years, sorry or maybe next year he`s gonna go to school.

Interviewer: so, do you think there is a positive relationship between partner support or support structure you tend to complain a lot and tend to feel alone and that I think it has an impact on the baby in an indirect way on the baby because when you are pregnant you have to be happy all the time you not doing it for yourself you doing it for the baby because one thing that I try by all means not to do is to get angry even though am angry him I just try by all means, not to be angry for much longer like after few minutes I will be like talking to my baby and I will be back to normal because I had a friend I think she has had two babies, she had a baby first time she had to give birth to a, the baby had already died [still born] still born yah after 5 months, 5 months to 6 months end then the second time around she gave birth to a still born again, 9 months, few weeks after like she's due so because I think she had stress with the boyfriend or something so I told myself like am very precautions when it comes to my baby I don't want anything that will irritate me that much because I know whatever happens even with the baby`s father I always tell him that you know try by all means not to get me angry or get me sad because you not doing it to me done because even the baby can sense, can feel or in the end can affect the baby in an indirect way so ……. Yah I think having a partner just helps because it`s more like you guys are bonding in a relationship and it`s growing and you banding even more and what not than not having a partner because you always feel alone.

Interviewer: what do you think happens to people who don't have partner support?

Interviewee: yoh! My gosh I think it's painful but at the same time makes you strong as an individual because you grow from it am the type of person that like everything happens for a reason, I believe that you know when you growing through a hard stage or whatever, I think that thing is preparing you for something bigger further down the line you`ll see success and what not, I think for me may be I would have preferred to have something like that but family wise I wouldn`t because I know that my mother would want to see the guy that impregnated me.

Interviewer: how do you think your family is going to handle things when you tell them?

Interviewee: ok with my family they are not gonna be happy but they will be irritated at the fact that I didn`t tell them that I was pregnant all this while and what if I gave birth like at 7 months you know so I think they not gonna like that, I have to lie and say sorry I found out when I was in hospital where as I knew before long time ago because I thought that my granny is gonna suspect something is gonna see but didn't see so yah

Interviewer: do you think it will have an impact on your future relationship with them?

Interviewee: I think with my gran she`s gonna understand, with my aunt and we close I think it's gonna go badly with her but then there is another part with me that says she will understand and then with my mom eish yah she`s just gonna be pissed.

Interviewer: When you think about the fact that you haven't told your family and imagine the reactions, do you ever feel stressed about it.

Interviewee: I don't want it to stress me, it's something that has happened so I think even with with them they expect it like at the back of their mind but then they, it's one of those things that which is giving you the benefit of a doubt that you wouldn't do it. It`s something that can happen to everyone so, am also a person you can't judge me by that I know my mother is gonna be really hurt but then she will be okay may be after a few months I just need to give her surety and show my remorse I guess she might be happy cause she`s gonna be so irritated I was thinking to the point where I have to buy myself a ring or talk to the baby`s father though I don't think he`s gonna want that because last year he wanted to engage me I was I don't want an engagement, like I said to him I don't want engagement that will take long I want engagement for 6 months, and then after 6 months you get married cause I don't understand having to have a ring for more

than 2 years, so now my chance of having to have a ring for more than 2 years, so now my chance of having a ring are very slim so I was just thinking at the back of my mind that may be if I buy a ring and say to my mom “ oh now am engaged” then she might be like “oh much more better” but then at the same time I was like no what if we break up with the baby`s father you know the whole thing engagement then lying to parents about the engagement and what not you know I don’t wanna go through that whole thing, if it’s meant to be that they get angry but I know they`ll get out if so it will pass it’s one of those things.

Interviewer: Thank you so much for taking part in his study

Interview 7

Interviewer: Thank you for agreeing to take part in this study really appreciate, so we are just going to talk about your pregnancy experience and your support system during your pregnancy, can you just talk about your pregnancy experience.

Interviewee: Okay my partner has always wanted a bby but them cause his working and am still studying I wasn't ready I told him that ahm no we have to go on the contraceptives, I told him that I cannot have a baby and I kept on taking morning after pills after morning after pills I even get sick from them and then it happened I took the morning after pill and it did not work so I don't know how that happened but then surprising when I did the home pregnancy test when I got my result I just smiled I was just so happy I don't know how that happened but I didn't cry or anything I was just happy then I told him of course I told a few friends but I did not tell family obviously and it only hurt me maybe after three weeks that okay am pregnant and now is my family going to take this because I think it`s them that I thought of the most I was worried about them the most because I am the first granddaughter and they just have so much hope for me so just disappointing them just crushed me but then besides that I was happy because my boyfriend was there for me, he supported me to the doctor, paid for everything and in our culture am the guy has to bring the family and they have to come and pay damages especially if you not married so he was willing to do all of that and I was just praying that his family is a humble because they couldn't just come to my family and start causing nonsense cause they have done a damage so obviously they were very humble when they came and I thank God for that. Okay besides that I am just happy that am getting the best treatments because I am gonna deliver at a private hospital, going to a gynecologist and I don't have to wait in long queues in clinics but obviously my boyfriend has to pay for consultations and when I give birth am gonna use my Mom`s medical aid, so yes I will be getting the best treatment and I just hope that when the baby comes it`s not gonna be hard.

Interviewer: And how did your family take it, your parents, when you told them you were pregnant or when they found out?

Interviewee: They were devastated, they were very disappointed in me ah that day I just remember that day, my grandma thought I was joking because she has never thought that

something like this could ever happen to me but what I realized was that I think they focused much on what people would say because in our community we have people that talk a lot, you know there people don't have anything going on for themselves and they start judging [yeah] so I was very disappointed at the fact that they cared the most about them the most and not me how I have to go through the worst I have to come to school pregnant everyone is looking okay lucky I drive and if am not driving my boyfriend brings me to school but still sometimes I have to walk on the streets and the pressure is on me, and the pregnancy is on me and not about them

Interviewer: How does it make you feel like people are looking at you, how does all that make you feel?

Interviewee: I think what matter the most is the fact that am happy so if you happy from within then it doesn't really matter with what other people think. I think that's the most important thing to be really happy, to be genuinely happy from within.

Interviewer: so aside from your own experience, how far along are you?

Interviewee: Am 32 weeks [32weeks]

Interviewer: So aside from your own experience in the last 32 weeks everything that you have experienced with pregnancy, before you got pregnant, what did you think pregnancy was like or what do you think if school have been like? How did you picture it before all this happened?

Interviewee: I didn't think one would get so tired during pregnancy I thought that when you pregnant, okay like your life it`s just on a stand still, it`s stationary and you can`t move on especially if you don`t have support from your family and your partner cause if your family decides to take you out at school there is nothing you can do, if your partner decides that you have to go to a government hospital cause he doesn't have money to pay there is nothing you can do so I thought it was a very devasting position to be in. I didn't think that you think of how amazing it is for a human being to be growing inside you like it's so amazing.

Interviewer: how does that make you feel?

Interviewee: it's just really weird and surprising at the same time, the feeling is good that my tummy starts so little (laughs) and then this thing just keeps on growing and growing it`s amazing.

Interviewer: and how have your emotions changed from the time you found out were pregnant and now? Have you grown to love the baby or do you sometimes have any feelings of regret or anything?

Interviewee: I used to feel like that sometimes especially if am fighting with my partner I used to be like okay "he`s the one who did this and I just can take anymore but then now I have grown to love the baby whenever anything happens, I have an argument with someone am just like I love my baby and I just hope this doesn't affect him cause he'll be able to love me unconditionally so yah.

Interviewer: so describe some of the challenges that you have faced in your pregnancy to date, the last 32 weeks, what have been some of the things that have been difficult or things you used to do that you can`t do anymore or how has it been balancing pregnancy with school work, family, how has it been/ what have been the challenges?

Interviewee: okay first of all …. uumm I was lucky that when I fell pregnant it was during the December holidays so with all the morning sickness and just feeling dizzy I was at home so that's fine but it was terrible because no one at home knew so I didn't have any support. I used to google things from the internet and obviously find things that didn't make sense, I didn't have a doctor to speak to I was just alone and okay with the challenges with my family there is just so much pressure because I feel like they just waiting for me to like say I just can't do this anymore you know waiting for me to slip, I don't know there is just so much pressure.

Interviewer: how does it make you feel having so much pressure?

Interviewee: because one thing my grandma said she was like now that am pregnant I need to pass more than I used to pass. So, I feel like what if I wasn`t pregnant I wasn't gonna pass as well so now I just have this pressure that I have to do well more than I did well as if I wasn't pregnant maybe I wasn't doing as well.

Interviewer: (……………….) what year are you?

Interviewee: 2nd year [2nd year okay, and if you don't mind me asking your age] am 20 [okay]

Interviewer: so, what do you think ahm, when you look at your surrounding your family your partner, what do you think they can do to help you cope with pregnancy? Considering that you are a student, you know, you need their support. What do you think they can do to help you cope?

Interviewee: okay they have already done their part in the health department ah when the baby comes I will also need someone to help me with the baby like a nanny of course so that will need someone whose gonna pay obviously it is gonna my partner and before I feel pregnant I used to drive and take taxis at the same time but now am just so tired to take taxis so I need more money, more allowance money, so I can put petrol money obviously its more expensive

Interviewer: so just out of curiosity the car you drive is yours

Interviewee: my mom left it for me [okay, so am just thinking your mom how did she react at first, I know some parents can be so brutal with news of pregnancy you know some parents would stop talking to you, some parents would take away resources, considering that your mom has left you drive her car during your pregnancy would you say she`s been supportive, now was it like when she found out and how is it ow with her, with your mother] okay first I think I need to tell you first  that my mom is an amazing person, she`s got an amazing personality, so even though she was really angry and disappointed because she was like she had me when she was 19 so she`s been working so hard for me to get where I am now but I hard to disappoint her and I think that's the thing that crushed her the most. Ahm but after my partners family came and she saw that they were reasonable people and they paid everything she calmed down and yeah she is supportive. Now, she asks me when I go to the doctor, how it went, giving me the car and wanting to know when am delivering so she can be there so she has been supportive.

Interviewer: okay, so you said you are 32 weeks at this point so when you look at the last 32 weeks in term of support when is it most important to you to receive support is it the beginning, do you feel you need more support at the beginning of your pregnancy, do you feel now or may be when you approach your due date that`s  when you`ll need the most support. When is it the most important time for you to receive support during pregnancy?

Interviewee: I think towards the due date because that's when I will be tired and I will just need my family to know that sometimes I won't be able to wake up early in the morning and do things, I think that's where now you need most support because in my first trimester that`s when I was still trying to digest everything and sometimes with my boyfriend always being there maybe from his side he was trying to support me but I had to digest it on my own I had to deal with it on my own so that I can move. I needed support to go through this ahm financial support, moral support obviously and yah just physically helping you with things around the house

Interviewer: and you have talked about financial support and moral support when you look at all these types of support what is the most important type of support that you want to receive, what do you consider to be the most important kind of support would you rather have financial support, would you rather have emotional support what is the most important support to you?

Interviewee: I think its moral and emotional support, [okay] because if you throw money at me say okay you can go to the doctor and not come with me it`s something else. If you just throw money at me and not ask me how my appointment was, wanting feedback to look at the scan then I would feel a lil bit worried. So, I think it`s moral and emotional support.

Interviewer: So, you have said moral support is the most important type of support, where would you rather get that from, who should be the biggest cheer leader, who do you want to be the biggest support system during pregnancy?

Interviewee: it has to be my partner [okay, why] because I feel like we both did this so he needs, whenever am down, he needs to be there for me and whenever he's down I also need to be there for him cause am sure it`s also life changing for him even though his stable and working and not studying but emotionally it`s also life changing uhm. I won`t say my family because I know they not those type of people so I don't have too many expectations from them.

Interviewer: so would you say your partner has been supportive in your pregnancy or if you had to rate him? What mark would you give him?

Interviewee: It would probably be an 8 [out of 10] out of 10 [so he`s been that supportive] his been supportive but then sometimes I understand if he also needs like a break [okay] I totally understand as I said it won`t be good all the time on his side so sometimes I give him his space

so that he can digest everything especially if his paying for everything am sure it hurts that he has to work so much and then he has to pay for everything as well.

Interviewer: okay so you have said his been great, what kind of support has he given you the most?

Interviewee: it is moral [moral support] because I remember when it hit me that am pregnant when I found out that m pregnant he was down, he wasn`t that happy as I was happy[okay] he was like because the first thing thought, he knows my family so the first thing he thought about was my family and then when I was down he was happy so when I was down he was down able to like support me and tell me everything is going to be fine, try to make me watch funny videos, like download cute pictures of mummy`s and babies so he was very supportive.

Interviewer: so how does that make you feel knowing that your partner. the father or the baby is been there for you through this?

Interviewee: am just really happy that his that mature because some guys, most guys wouldn't do want he has done, am just happy that we had a baby cause with the mom its different you have no choice but to grow up and understand your situation but with a guy its different so am just happy that we had a baby when he was at that level because it`s difficult for him to adjust from being I don't know going out every night doing crazy things to having to be responsible and stuff so am just happy that he is a responsible and mature person [okay that`s great]

Interviewer: so, let`s talk about after the baby comes what kind of support do you feel you`ll need the most after the baby comes? Do you still feel moral support should be your biggest, the most important support to you after the baby comes?

Interviewee: after the baby comes I think it should be financial support because if my baby is hungry I won`t be able to be happy therefore I won`t perform well at school. If I don't have a nanny I won`t perform well at school, so it is financial support. If my baby doesn't have clothes or warm clothes to wear I won`t be happy [okay] so yah

Interviewer: and after the baby comes, just talk about your plan what are you planning how will you juggle school and motherhood. Where is the baby going to stay, where are you delivering from, whose taking care of the baby?

Interviewee: okay my plan, am delivering on the 9th of august, so my plan is to stay off school for two weeks

Interviewer: sorry before you go on how are you delivering are you having a natural birth or are you having a c section?

Interviewee: hopefully a natural birth if there are no complications so I am gonna stay off two weeks and we usually have practical in my dept. so if there is anything that I have to come to school for I will be able to come [okay] but yah am planning on breast feeding as well ah then we`ll find a nanny hopefully if we don't we have a close family member, she's my family friend and he`s family friend so we trust her a lot she`s older obviously so yah if it doesn't work out with the nanny she`ll be able to help, here and there.

Interviewer: how does it make you feel knowing that you have people who are willing to help you take care of the baby?

Interviewee: it makes me feel happy that am even getting support from people on the outside and obviously his family yah it makes me feel happy.

Interviewer: let`s just go back to your partner and his support, tell me about your doctor`s appointments, your antenatal sessions, how supportive has he been with those?

Interviewer: ahm okay my recent, before my family knew I didn't have a gynecologist before I went to a GP, I used to go to a GP until 3 months. So he used to go with me at that time but then now that I go to a GP his always working so like I go alone or with my gram but I know he wants to be there but if he can be there he will be there and I want him to be there when I give birth, I want him to hold my hand when I give birth, I want him to hold my hand when I give birth and yes.

Interviewer: so, he's been supportive or the times that he hasn`t been there is because he has other commitments he has to work, so do you feel like that is a problem to you?

Interviewee: no because he will call me after the session and he`ll ask me how it went, he`ll want to see the scan, he`ll will want to know how progressive I have been yes.

Interviewer: so, what does it mean to you to have all this support during your pregnancy especially from your partner? When you look at yourself and look at others who are also pregnant and probably haven`t been you know this lucky haven`t had people to support them. How does it make you feel to know that your partner is there for you and has been supportive? What does it mean to you?

Interviewee: it means a lot and I believe in God, I believe that it’s his doing it`s not by me being smart or me being I don’t know it`s just his doing and am really grateful for that am really grateful, it`s really a blessing from God.

Interviewer: okay and do you feel like his involvement in your pregnancy has influenced the decisions you have made in your pregnancy? Do you ever find yourself failing to decide without him or always have to run the decisions by him? Has his presence influenced the decisions you make?

Interviewee: my family`s presence has influenced the decisions that I make, because my plan was to go to a hospital because I didn’t see much of a problem going there considering the money that his paid at home, because my mom said that he has to pay for all consultations and I am gonna deliver with her medical aid, he has to pay the money back so I was like with paying for the nanny and buying food for the baby I don’t think he would be able to balance everything with paying her back the money so I was like I might as well go to a gout hospital cause it`s just a matter of like two days but my family were like “no we want you to get the best care so there were like no it`s fine if he doesn’t pay but on his side he just wants the best for me as well, I feel like I have to inform him but them his always like just do what’s best for you”.

Interviewer: and I just want to know when you look at how long have you been with him?

Interviewee: for 9 months

Interviewer: so when you look at your relationship before your pregnancy and your relationship now has the presence of the baby coming affected your relationship in any way positively or negatively? How has your relationship evolved over the last 32 weeks?

Interviewee: I feel like we should have waited because we had just started dating [okay] we should have waited to at least know each other well, but I feel like when I date I don’t just date

for fun so when I get into a relationship it’s because I want to be committed so lucky I found a person who had the same mentality as well so it wasn’t much of a problem when we found out this news cause obviously you`ll be committed to each other forever cause of the baby uhm sometimes it does take a toll on our relationship because sometimes you find that his a bit I don’t know like moody I don’t know

Interviewer: and how does he handle your moody, are you ever moody? How does he handle those?

Interviewee: I would tell him that am depressed and I need space and he`ll give me space
[all right]

Interviewer: so, do you think there is a relationship between mother and child health, your health and the health of the baby, do you think there is a relationship between that and male partner support? Do you think male partner support has on influence on your health and the health of the baby?

Interviewee: Yes, a lot because if he decides to go and sleep around that`s gonna affect me and my baby because there are so many diseases you can catch, he`ll just be putting both our lives at stake.

Interviewer: what would you imagine you would have done if he was not supportive?

Interviewee: I honesty do not know, honestly don’t know but I would be very depressed, I would be very depressed [why] because I feel like he should be there for me and the baby I feel like he should be there for me and the baby.

Interviewer: so, if he wasn’t there for you and the baby your health would be affected? How would you have been mentally if he was not involved?

Interviewee: I would have been obviously shocked and traumatized and like how could you just sleep with me and have a baby with me and then just leave me like that but I would have to adjust just like I have adjusted with being pregnant, I would have to adjust and just accept the situation and just love my baby unconditionally.

Interviewer: Thank you so much for taking part in this study again, I really appreciate.

Interview 8

Interviewer: Thank you for taking in this study. So, can you just talk about your pregnancy experience, just how has it been? From the time you found out you were pregnant?

Interviewee: When I think of my pregnancy I just I just feel so hurt because it's one of my focus at the moment but so I feel like I just disappointed my parents because like they don`t want anything to do with me …. Am just weak.

Interviewer: Do they still don't anything to do with you?

Interviewee: Yeah [upto now] upto now [how far] though their paying my fees

Interviewer: How far along are you? How many months or weeks are you?

Interviewee: It's like I still have to go to my ultra-sound [you are not sure] the due date and actual period [but where about are you] three.

Interviewer: Okay so when did your parents find out

Interviewee: They found out ahmm because I actually told them [you told them] by myself I couldn`t live with it I knew it was a bother and when I saw them I feel like I betrayed them so I just turned my attention to my mom it was when the end of March it was about to end then I told her, I told her, and then I told my father.

Interviewer: And how was her reaction? What was her reaction?

Interviewee: But my mother is special she tried to understand the situation because she knows that these things happen but the problem is with my father [okay] but she was just disappointed.

Interviewer: What was your father`s reaction? How did he behave towards you after that point?

Interviewee: He couldn't speak to me [up today] no. He speaks to me when he talks about how much I need, those things, those stuff his just concerned with my education only but there is nothing more.

Interviewer: And how does that make you feel? When you think about your parent's situation how does it make you feel?

Interviewee: I feel abandoned alone although there are others who support me I feel like [you needed their support]

Interviewer: What do you think would make them support you? What do you think you could do or what do you think should happen in order for them to start supporting you?

Interviewee: When the baby is born [when the baby is born] yeah [okay]

Interviewer: So, tell me about your relationship with your parents before you got pregnant how was it?

Interviewee: It a very close relationship especially with my father he loved me so much but when this situation came up he was just disappointed before there was nothing wrong, there was nothing that could come between us but now it has changed

Interviewer: And tell me about your friends here at school, at home, how has it been?

Interviewee: I do have supportive friends who do have babies so they know my situation they have experiences yah [yah at home] at home Iam just staying in my room but back at the residence they do understand they also understand there is nothing new because many girls get pregnant.

Interviewer: If you don't mind asking how old are you?

Interviewee: 19 [19]

Interviewer: And what year are you doing here? Here in school. How far have you gone with your studies?

Interviewee: oh! I am doing my first year.

Interviewer: Okay, can you tell me about, you have talked how things have been since you got pregnant. Before you got pregnant what, did you think a pregnancy should be like? How did you

imagine it? What are some of the things that you thought pregnant women should do or should receive that you have not received?

Interviewee: A Person whose pregnant just needs support, loved, even though how bad the situation is or how it happened but when I look at on the pregnancy of mine I just feel like it's a mess up. Support from all perspective, family or boyfriend or husband or however, friends so that she can feel happy it is important.

Interviewer: What do you think happens when women are not supported when pregnant?

Interviewee: they struggle to keep the pregnancy and some will get rid of the baby, but if you keep it at the end they can even get unhealthy babies

Interviewer: What do you think causes women to have unhealthy babies?

Interviewee: Stress, is one of the factors that contribute, even not having healthy food

Interviewer: So, when you think about your pregnancy, what are some or the challenges you have faced? What difficulties have you had during pregnancy?

Interviewee: Things that have happened at home, many bad things because the relationship is no longer the same

Interviewer: Does that affect you?

Interviewee: A lot

Interviewer: How?

Interviewee: I feel neglected by any own family, I feel like they have abandoned me.

Interviewer: What were you expecting? What would you have loved to get from them?

Interviewee: They have the right to be like this. They have but on the other side I just feel like they not fair. I know that I make a mistake okay fine but the way they are doing it it's like I don't know how to describe this …it has been difficult

Interviewer: And money do they give you money for food, do they still give you the money they used to give you?

Interviewee: Yeah but for the clothes they are no longer they told me that they will no longer buy me clothes

Interviewer: So, whose buying you clothes?

Interviewee: My boyfriend [his been supportive] every step of the way

Interviewer: How did he take the news when you told him you were pregnant?

Interviewee: He supported me

Interviewer: How did he react when he first found out that you are expecting his child?

Interviewee: He was shocked, but he accepted it at the first beginning when I told him, he has just taken responsibility when I told him.

Interviewer: So, what does he do for you?

Interviewee: Supportive

Interviewer: Does he come with you to the clinic, have you been going to the clinic?

Interviewee: It’s for once [and he comes with you] no he staying far from here his staying where am coming from [okay] so attending the clinic nearby my res so am away from him

Interviewer: So, does he call you to find out how you are doing with the baby

Interviewee: Every time

Interviewer: And how does he send money when he needs to send money?

Interviewee: No, I go, I go back at home after two weeks, every two weeks we meet there sometimes we meet in town.

Interviewer: So, when we talk about support, what is the most important type or support to you? What support would you love to receive? Is it support in form of money, is it support in form of

a emotional support or you just want someone to talk to or you want someone to encourage you, help you with school things. What is the most important type of support to you?

Interviewee: I just want support from someone who`ll understand me and my situation, who`ll not judge me but support will bring happiness to me, being loved.

Interviewer: Does that person have to be your boyfriend or it can be anyone?

Interviewee: No, it can be your friend, it can be anyone, it doesn`t have to be my boyfriend, it can be the member of the family, friend, yah [you just need someone] yeah someone who can understand

Interviewer: Okay so what kind of support has your boyfriend been giving you?

Interviewee: All kinds of support when I talk to him to tell him what I need he just gives me, when we talk he understands so it's good.

Interviewer: What do you think about male partner support do you think that a man, it is important for a man to support his woman when she`s pregnant? Do you think it is something important?

Interviewee: Yeah, I think it is important [why] because sometimes you feel like person it's his mistake you know because if it wasn`t for him I actually wouldn`t be pregnant so he has to be there for me, so the boyfriend needs to be there

Interviewer: So, what do you think would happen to you if your boyfriend was not there for you?

how do you think you would feel if he was not supportive?

Interviewee: I would be disappointed and sad to have the pregnancy in the absence of his support.

Interviewer: What do you think about women who do not get support from their boyfriend? ……….. how do you think they handle it?

Interviewee: It must be hard for them, it must [why] because they are daily ignoring that their father is not there for them knowing that you are only alone in the situation, your partner is no longer there it must be hard.

Interviewer: And how do you think it affects them?

Interviewee: I really don't know

Interviewer: So, what does it mean to you to be supported by your boyfriend? How does it make you feel knowing that your boyfriend supports you in your pregnancy?

Interviewee: I feel supported and know that I can get help from him

Interviewer: How does that make you feel inside?

Interviewee: Appreciated

Interviewer: okay what do you think about male partner support and mother and child health? Do you think there is a connection if a woman is being supported do you think there it is a connection if a woman is being supported do you think it affects her health or if she`s not being supported do you think it affects her health and the baby`s health

Interviewee: Yeah [how] because I would probably have stress which will affect my health so having stress all the time thinking about many bad things.

Interview 9

Interviewer→ Thank you for taking part in this study. Can you talk about your pregnancy experience? How has it been?

Interviewee→ Firstly, I did not know that I was pregnant.[until] the time I realized I was pregnant it was uhm...in the beginning of February where I started not going to my periods so I was like let me do a pregnancy test, first asked my friend her name is Maggie, she brought me like three pregnant tests and then did the pregnancy test, they all came out positive and I was very shocked because everything went normally until the end of January where I started to realize...so for me I think it was a big step and I didn`t expect to happen even though I used doing sex a little so from there I asked my friend to help me how I break to tell my parents even how I would tell my sister because the time I was staying with my sister and her husband so it was difficult at that time because it was the time were I just found out...so...at first I didn`t tell her a waited two days, I told her two days after the day I found out [yah]... I was like I need to tell you something... I don`t have periods it's been like a week now so I don`t know what is going on, tried to see a doctor he was like okay let`s just wait and then on a weekend, it was Saturday she called me and then she gave me a pregnancy test and I did it and it was positive again...still couldn`t believe it because I didn`t expect such a thing to happen and to me and was shocked... but she kept me calm and advised me what to do, how we are gonna tell our parents what not.

Since am not staying with my mom, she went to Joburg so she came back on 14th February at my parent`s birthday okay fine, I called my sister to come and tell mom whilst I was at home and then she told her she called me, she asked me questions "whose is the guy "where is he from" and what I was thinking at first...you know parents [was she angry?] she was but she was not shouting...because really if she shouted...really what was the point because the damage was already done, so I said am sorry and what not and then we called the guy...and my mom asked some questions, where you from, who you living with, yah the guy told them he stays in Pinetown, his with his own house, he works for his own company [...] from there she also came, she calmed down because you

know the guy is got something unlike the taxi drivers [...]so from there she asked me, she remembered one thing, on December she bought me like pads [yah] and she asked me about that thing, “why did you tell me I must buy pads when knowing very well you pregnant” she even saw the blood on my pant, so I told her that I didn`t know, she think that I was lying and what not even my sister I told her so basically from there the only person I was scared of it was my dad because [he had no found out yet] yes... but he was suspecting something you know because every time I give him food at night he always give me that strange look you know but then I was like okay fine maybe he doesn’t see anything and then a week or two later if am not mistaken...my mom went back to work and then came back she went to buy my granny`s house she asked my granny to tell my dad because she was afraid of facing dad and telling him that am pregnant so... my granny lives at Hillcrest they call my dad, it was a Friday morning I remember the day, Yoh! So, they call him they say he must come quickly so he came, he goes to my granny`s house and then they told him.

The only answer that he gave them, he only told them that he was suspecting me because he said different things [...] that`s the way he found out.

Interviewer→ And what was his reaction in spite of that he already suspected?

Interviewee→ He didn`t talk to me for like three days to four days and then afterwards he started slowly talking to me...he never asked me about the guy, he never asked me about my pregnancy and what not [and now] and then I was very shocked, it was 7th April, on my birthday, my aunties and cousins planned the day for me. They even did a baby shower on my birthday all together [wow] he also contributed [that was in April] yes [okay, so you are 9 months now] yes, I was very shocked that he contributed to this baby shower whole thing and what not because I didn`t expect him. He`s such an angry man, you know men, its dad you know so I didn`t expect it I was like oh.

Interviewer→ How did that make you feel knowing that [he contributed]? yah.

Interviewee→ I was happy, was happy because the thing that made me happy, am always with him even if I want something the first person to ask is dad even if my mom she`s at home, I go to dad first [so you have a close relationship] yes. So, the pregnancy thing made me frustrated because its gonna break our relationship now yah.

Interviewer→ But how is it now?

Interviewee→ Its fine, it went back to normal [now you talk and everything is] we talk about everything but except for the pregnancy whole thing situation.

Interviewer→ Why do you think he avoids talking about your pregnancy?

Interviewee→ It makes him sad, I even see in his eyes because sometimes I think it comes back to his mind like" no, this child just ruined her life" you know.

Interviewer→ So was your boyfriend called for damages and stuff?

Interviewee→ At the moment he hasn`t paid [yah, but was he called, did they have the meeting, did the family talk about the pregnancy] he is the only adult now so he is the only one looking after family but he has an aunty who lives by Nonthle [okay] basically they do not have a true relationship with his aunty. Family drama.

Interviewer→ Do you think uhm part of the reason why they haven`t discussed the damages yet is because of your father not wanting to talk about the pregnancy?

Interviewee→ uhm no... I think it is the guy...am not sure if he`s, I know he always say he is afraid of my dad and the thing is he lives very far, he`s only been here like...he came here for work but stays in [...] so he`s the type of a person who wants to do things for himself, he is no longer relating to the family because the family somehow, he says so betrayed him after he lost his parents what not you know so I didn`t wanna dwell on that family drama because am very emotional but.....by the look of things he will still pay damages since we still calling even though we hardly see each other even if before I was pregnant we hardly see each other because but we do contact [okay]

Interviewer→ So in spite of not seeing each other often how is your relationship?

Interviewee→ Okay at the moment, uhm it`s dry to be honest, it's very dry compared [compared to the way it used to be] yes since February.

Interviewer→ When did you find out that you were pregnant?

Interviewee→ February [February]...it was all how are you, how`s the baby [and that`s how the relationship is] no [uh] it's just people the way they...because I just have accepted that okay fine if he calls okay fine, if he doesn't (sulks) okay fine.

Interviewer→ Was it like that before?

Interviewee Not really...that thing obviously started happening during December...yah [okay] because we were always fighting from there since December like I don`t know it was me or my pregnancy hormones but I was very rude if I remember I treated like nobody really gets me irritated and shout [...] for weeks and afterwards call each other, it's only the beef started in December and now if I tell him it was my pregnancy hormones, he was like " no you were just rude, you didn`t want me anymore, you have got a new boyfriend" you know and I was like no.

Interviewer→ And how does that make you feel knowing your relationship is not what it used to be?

Interviewee→ Sometimes it makes me sad... but basically, I am the type of a person if things don`t go according to what I want for a long time I allow it to happen and then I try to fix it. If it fails like two or three times okay fine I will just watch him whatever you want to do, do it. If you say so am gonna reply to those things he said, if he calls he calls if he doesn't he doesn`t, if I feel like talking to him I call him but I do that most of the time because I always call him, if he doesn't pick up the phone you know guys it will be bad then you see my missed calls then you gonna get aback if you don`t its fine.

Interviewer→ Okay so tell me about his reaction when you told him you were pregnant, how was his reaction?

Interviewee→ He firstly, I used my doctor`s phone because me and my doctor have a close relationship I used him so the time I did the scan I first called my sister, told me to call him, I was like no I want to tell him face to face, what not, then she said no call him, then end up calling him am like I went to the doctor just make up the whole story I was sick, and then he said “what`s wrong with you” I was like okay fine I went to the doctor, I did the pregnancy test, I did the whole test what not and then I just found out that am pregnant and he was like “what!” am pregnant, and he was like how far are you, am 5 months and he was like “ I know I have been seeing you that you are pregnant but was waiting for you to tell me” and I was like WHAT! How come you did that? Because he reminds me he used to say uhm are you getting your periods? Yes, why? The minute I mentioned yes from that answer his faced just changed am only picking up now... when am recalling everything, now am asking him why are you asking me periods and what not? No am just asking. Do you want me to take you to a doctor? Am like no, no let’s go to the doctor? Am like no am not sick no. An then it’s all gonna end up like that and am not gonna go to the doctor then he reminds of that and just like oh you kept hiding, you know that you made me pregnant and you kept quite the whole time so you were waiting for my response and he was like “am sorry for being selfish I didn`t want you to abort my baby “because he thought I was gonna do that no girl should do that and I was like no I wasn`t gonna do that do that why do you think I would do that ? I wasn’t so sure you would keep my baby or you`ll abort and then lie, make up stories and what not. I was like oh okay fine forget about that the baby is here and if am gonna do abortion its either I die or the baby dies so am not gonna do any stupid from my own [...] he was like how far are you ? am like 5 months, he was like WHAT screaming you know guys, am gonna be a daddy [so he was happy] he was happy [okay] and at the end he was no am not believing you. Are you prank calling or something, I was like nooo I am notgoing to come there after work but he didn’t come due to him end up working late so after like 8 I don`t go out [okay]...so...it was on the 11th of February it was on the next day I was here at school, he came to pick me up and the first thing I did when I went to the car I just opened my wallet and said please the scan. He was like “what is this” and then he turns over and I

was like it's the baby look at it, he was its not my baby is that my baby inside there? Yes, am like he was like no no no no wait am gonna watch this thing while you park [...] he was shocked I think he had this mentally I was playing with his mind so he was shocked when he saw the scan because the reality basically struck him. Then we went to Glenmall and then we parked by the garage then he grabbed the picture and watch it then he looked at me, and I just watched him I think he couldn't even believe and then I was like are you okay? he was like am still shocked are you sure you pregnant? I was like touch my stomach. If you know me very well you can feel there is something inside there and he feel it. Like yoh so how are you going to tell your parents. I told him the whole procedure He then asked me how I was going to tell my parents, I explained the whole procedure how I was gonna tell them, he was like am fine so am starting to save for damages because thing is he didn`t plan but then I was like you told me way before how come you haven`t planned but you know we get money and then we use it wasn`t even in my head I was like okay fine. And then he just asked about the baby stuff and what not how we gonna do it, how we gonna see the doctor and then we discussed such things. After that I start going to the clinic, even if private or public they want to see proof that you been checking up you baby so you can use a doctor but they recommend clinics because clinics it's just the regular thing going on. After that I entered the clinic, first I was like eish so many pregnant women (laughs) at my age.

Interviewer→ And how old are you?

Interviewee→ I was afraid that my neighbors will see me going to the clinic especially to the maternity dept. And they will start gossiping me.

Interviewer→ How did it make you feel when you think about people knowing about your pregnancy?

Interviewee→ At first, I was like eish. They gonna say bad comments...you know how people especially townships...so

Interviewer→ Did you feel like people had different expectations of you? Do you feel like you family had different expectations of you?

Interviewee→ My community...okay like for my community it was like a huge achievement to them for me you know how people are jealousy for them it was like "yeah finally we got her...wara wara [how did that make you feel] because basically last year I had so many goals and I used to talk I want this, I wanna be like my sister because my sister basically she doesn't have a kid only his husband came with a child.

Interviewer→ How did it make you feel knowing that your family is disappointed?

Interviewee→ I cried at night [you cried] yes and then during the day, I was a normal person and then at night it strikes me. They gonna take my dad otherwise now since am pregnant [they gonna take your...] dad, my dad is such a person that he knows people and he`s the guy that can be a role model to somebody, he doesn`t have much he`s is just an average guy but people do come to him for advice [...]. My dad is a respectable man in our community and people come to him for advice.

Interviewer→ So how far along are you in your pregnancy?

Interviewee→ I am... am 37 then next week is 38 [so you are due anytime] its 37 and next week is 38.

Interviewer→ So have you prepared everything for the baby?

Interviewee→ So far, I have just prepared clothes...ah I think am only gonna buy cosmetics on the weekend or Monday.

Interviewer→ And if you don`t mind me asking how old are you?

Interviewee→ 19

Interviewer→ Okay so what are some of the... before I go to that before you got pregnant what did you imagine pregnancy should be like? What did you think ...what kind of an experience did you... wish your first pregnancy to be pregnancy?

Interviewee→ Basically I wanted to be pregnant by the age of 23 or 24 just like my brother and then by the time I wanted to be... leave the house even if i don't have a car but to have my own place where I can have a big room for my baby and what not you

know those dreams and then have like a proper relationship with the guy and all maybe we in the process of getting married or something...yah that was my idea.

Interviewer→ How does it make you feel that it did not happen that way?

Interviewee→ It makes me sad bruh because you know what the minute I open those book-novels, the bathing of the baby, the whole setting and the whole idea just comes again and makes me sad.

Interviewer→ So what are some of the challenges you have had during your pregnancy considering that you are 19, you stay with your parents and you are in school? What has been difficult during your pregnancy?

Interviewee→ Time management I won`t even lie... time management for school [its ben difficult] yeah [why] a::hm when it comes to dressing and getting things done...okay the dressing part am slow literally because I choose what I wanna wear since am pregnant its even less because some of the things are too small, they don`t fit my stomach I have to change... you know sometimes I even forget that the things are no longer fitting me so I have to change. I sleep planning my outfit but when am getting dressed am like eish this huge tummy now have to accommodate it [what else]...school wise I think my brain is functioning too slowly I won`t even lie even if am reading maybe (laughs), I will read this like maybe a paragraph this size i wouldn`t recall the important of the paragraph easily [you don`t concentrate] yes.

Interviewer→ Why do you think that happens?

Interviewee→ Ahm...I think it's how the relationship is with my baby daddy [you think a lot about that] yes [why do you think a lot about it] I don`t know ...to be honest sometimes I think he is going to run away since he hasn`t paid damages and he can see the other girls and forget about me (laughs) you know, you know how boys are.

Interviewer→ have you tried to get him to pay or no one has made an effort?

Interviewee→ Basically, I have tried, my sister have...I don`t know what he's thinking at the moment...what are his plans or intentions or he`s just some guy whose gonna do

shit to me and then he would be like "hey I don`t know you, who are you, who made you that, I don`t know you".

Interviewer→ How would you describe your relationship with him right now?

Interviewee→ Okay talking from the just two past weeks it's like the first time we know each other. I do not know if it is going back to normal or it is because I am about to give birth. I don`t know... so am just really shocked [so it hasn`t been okay] for while it hasn`t been okay but now since like three to two weeks back everything is just like smooth [so things are okay with you] yes.

Interviewer→ When you think about the future. How does it look like?

Interviewee→ The future... am type of person I think if you make me angry at a certain limit...am sure if I would say I hold grudges or not but somehow somewhere I will make you suffer because you make me suffer even if I did love you but I have to make something that gonna just bring you down, not down but... keep yourself calm, am that type of person, so by looking at the future, am still gonna do some things that will make him suffer (laughs) just a little, not too much [just to get back at him for what he has put me through] and make him think and realise that he is not just a boy but a father and make him think and realise... you know you just not any boy you are a father just be responsible because you know I don`t know if it's all the guys or males... the mentality of taking responsibility I think they don`t have that [okay] so I wanna make him to have that thing in mind that no I have responsibilities and I must act accordingly, that`s all I want.

Interviewer→ So apart from being slow at school and struggling to dress up what other difficulties have you faced with pregnancy?

Interviewee→ Eh I talk a lot, I think I talk a lot and analyse things...too much [you over analyse things] yes...and somehow even the little things am like why, maybe this maybe that.

Interviewer→ And your relationship around you your friends your siblings at home, how has it been?

Interviewee→ My siblings its good [they have been supportive] yes very [and your friends] both sides, very supportive.

Interviewer→ How does that make you feel?

Interviewee→ It makes me feel better, sometimes I do not even realise that I am fighting with my baby daddy [because] they are always here if I need something they assist me ask questions may be for instance when does the baby turns to head down, they talk to me and then there is this thing in our culture, Zulu, if you have given birth you are not supposed to be cooking for like three months since you are bleeding and then for instance most of the time I stay with my daddy so my dad is not is not allowed to eat the food that I have cooked so somebody is gonna do that duty. I can't do the whole thing and am not allowed to watch TV because everybody at night watches TV so am supposed to be kind not for bad reasons for good reasons bond with my child you know [so you do that] yes we do that and what else uhm you not allowed to visit your friend with the baby up to he or she reaches three months or after or after you can go out because they believe you know those witch thing they do have that thing because is still happening because they believe the baby is gonna have bad spirits.

Interviewer→ So it's just because you are pregnant that you cannot visit a friend with a baby?

Interviewee→ Yes.

Interviewer→ If you were not pregnant, you still wouldn`t?

Interviewee→ If am not pregnant, am not having a baby so I would any how [okay] it's just that you having a small child right now, what else am not allowed to walk around at night.

Interviewer→ Okay you said you are 38 weeks...

Interviewee→ 37 if you want [.....]

Interviewer→ So what do you think the people around you can do to help you to make it easier for you?

Interviewee→ Okay what worries me at the moment ...after giving birth and then whose is gonna take care of my baby [you haven`t planned that yet?] Yes, because basically there is nobody from outside because I don`t trust people easily [so you haven`t] like you know women and those ritual things they do and the motives but I don`t trust people especially women because they are very good in that department (laughs) [you haven`t planned yet on how you are going to take care of the baby] yes

Interviewer→ So, at what stage of your pregnancy do you feel you need the most support from your family, your friends the baby`s father...do you feel you needed the most support at the beginning when you found out you was pregnant or do you feel you need more support now that you are at the end or do you feel you`ll need more support after the baby`s born? Okay as for me I think I do need but I have support but believe [but the most when would you rather have it the most] throughout the whole pregnancy [throughout the whole pregnancy] even if the baby is here I need support why they should stop at some stage or continue at some stage I think I do need the support.

Interviewer→ What about when the baby comes...what kind of support do you think you`ll need?

Interviewee→ I do have experience on how to bath the baby, feed the baby well when its crying what's wrong they should tell me what to expect if am not figuring out they must assist me [so you feel you need people around] yes [so your mother is not taking leave or anything to come and...] she is arriving next week Monday before giving birth [okay she`s taking leave] [for how long]am not sure [okay so she`s being supportive] very [she calls, very supportive she even reminds me have you been going to the clinic recently...when she comes back i give her my card to read it and ask what is this basically about because sometimes they don`t explain it fully. They just summarize the whole thing.

Interviewer→ What is the most type of support to you? What kind of support do you want to receive during pregnancy and after the baby is born? What is the most important type of support?

Interviewee→ Emotionally because physically I can manage...I think its emotionally [okay why] because am afraid that if he or she cries am gonna cry to... in such a way I wouldn`t even be able to assist him or her.

Interviewer→ And who would rather receive the biggest emotional support from? Would you rather receive it from your mother or your partner?

Interviewee→ I do get it from mom sometimes...I don`t know am not able to talk everything...but with my boyfriend I do you know every everything I just summarize with her but with my boyfriend I do.

Interviewer→ So does he give you that or would you or is it something that you would wish he was giving you? Him talking to you being there to listen.

Interviewee→ Not too many [okay how would you rate him in terms of emotional support] so far so... I think am gonna give him 3 or half [so he`s not usually there] out of 10. Yes, he is always busy he`s got business you know, can we talk like 8 `0` clock am gonna call you am like okay fine and am gonna remind him maybe he`s gonna call me like past 9 by that time am dizzy, am not able to talk am like I will call you in the morning. The first thing I always wake up around past 8 by that time he is in his office [ so you don`t really talk much] we do talk we use those little times we have to talk but it becomes an unfinished business and the next time (snaps finger) I call him it becomes something else [so there is a lot of things that you don`t get out of your chest because] we do talk but most of the things it's an unfinished conversation [because he`s busy, so would you want him to create more time for you] yes.

Interviewer→ What would it mean to you if he had to create more time?

Interviewee→ I think it would be paradise or something (laughs) [it would be paradise] yes [okay] because we would have that bonding session, talk about stuff...talk about the baby [you want more emotional support from him] yes.

Interviewer→ And financial support how has he been?

Interviewee→ Financially (sulks) so far i do not know if he'll do something big huge thing for the baby but so far. (shakes head).

Interviewer→ Was he supportive during the baby shower did he contribute, was he even there?

Interviewee→ I did not even tell him [you didn`t tell him] yes just when I was at the baby shower [wow why] (laughs) because we had a fight, the time we weren`t talking to each other, so even now am quite about it you only gonna knew most of the stuff when it's a new baby, because I think he has this mentality I want a boy if that child is not a boy you gonna see it for yourself [oh he wants a boy] am not sure if he`s joking or maybe his just saying.

Interviewer→ So he wants a boy?

Interviewee→ Yes

Interviewer→ You don`t know what you are having yet?

Interviewee→ I do [you do] it's a boy... I didn't tell him [oh okay so you want to surprise him] yes

Interviewer→ How would it make you feel if you knew you were having a girl and he was talking about wanting a boy? How were you going to feel?

Interviewee→ Its nature ... am not in control. [Do you think he was going to mean what he said] ahhh. [was he really going to neglect the baby if it was a girl]. sometimes it is hard to judge because of things that's been happening because people do surprise you in the end... so I can`t tell.

Interviewer→ So where are you going to have your baby from?

Interviewee→ I have been told that at Hillcrest private hospital, it is a beautiful hospital I never been to it.

Interviewer→ How much does it cost?

Interviewer→ It should 8000 or something I don`t know and I cannot afford but he can afford that [so it's out of the question, would he pay for that] he can [he can afford it]

(coughs) if he does pay since I am 9 months and I can come and pay for all facilities and then send documents and book for my bed, anytime the baby wants to come I can go easily since he hasn`t done that most probably am going to go to a public hospital which I don`t like, because since we don't have public hospital around here my area, I have to come at Marian hill Hospital but in times of need I don`t have a choice because it's the most nearest hospital.

Interviewer→ So at the point you are not sure what is going to happen?

Interviewee→ Yah [public hospital]

Interviewer→ If he pays at the private you`ll go at a private?

Interviewee→ Yes [if he doesn`t] I go to a public hospital?

Interviewer→ Do you think he can afford it? Private?

Interviewee→ He can.

Interviewer→ So why hasn`t he paid? Have you suggested to him? What did he say?

Interviewee→ The last time it was Monday when he was like okay he first asked me I didn`t even start the conversation he was like "how far are you right now" am like 9 months "so when are you due" anytime this month I didn`t tell him the date that I receive from the first scan and the last scan... so that anytime , any day this month "so are you going to give birth at the hospital" I was like if I did afford I would but I don't have cash so am gonna go the clinic and he's like "whose baby is gonna be delivered at the clinic and what not." am like if you don`t want me to deliver at the clinic do something about a private hospital maybe Hillcrest because it's the nearest compared to Marianhill he was like "okay... I will make a plan" that was his answer maybe he he's still making it a plan even today so far me if it happens that my water broke am gonna go to King Edward or any public hospital [king Edward is near your place] its down, yes it's the nearest hospital its public because st Augustine is private, Entabeni is private

Interviewer→ So what kind of support do you feel you will need the most after the baby comes?

Interviewee→ First month maybe by the second month I will be used to it but first month... I need 100% support [okay what kind of support do you need emotional support, do you need financial support? What will be the most important kind of support for you when the baby comes?

Interviewer→ Emotional support, yah because some of the things i have read about it and they have told me so am expecting them to happen... then I think the thing I will need most is financially [financial support] I think it is very costly they get sick easily

Interviewer→ Who is going to provide that financial support? Is it your mother or it is your baby`s father?

Interviewee→ Okay my family is very supportive...my mom has this I don`t know it's an ego or some sort "I can raise any baby and what not, am working. That's my granddaughter because she`s the one who always giving me money when am supposed to see the doctor [okay, she pays for the scans] yah [so far your boyfriend has not paid for the scans] NO [he hasn`t given you any money] NO [he hasn`t bought anything for the baby] NO [when is he planning to?] I don`t know I always ask him [and what does he say?] "am gonna come around, no give me 10 minutes I will be at Barclays" and then 10 minutes he turns it into a month...whole day, a year [wow]. That`s the answer am getting but I don`t wanna judge him [yet] at the moment I just wanna see what he will do when the baby arrives.

Interviewer→ So generally his support has not been very... much?

Interviewee→ Its weak [its weak okay]

Interviewer→ How does it make you feel knowing you are in this state and you need his support?

Interviewee→ It makes me angry [it makes me angry] not to anybody but to him and when I want to voice out (snaps finger), I just send sms to provoke him if he talks am just gonna blow.

Interviewer→ Does it affect your wellbeing when you are angry? Do you find yourself crying, feeling sad feeling depressed?

Interviewee→ Yes there was this one time I was depressed in such a way that I didn`t go to school for a week [wow]. I told him that am sick but he was like "what`s wrong with you, is my baby fine" the baby is okay but if am not well [so it's more about the baby than you?] Yes. If am not well, automatically the baby will be affected so that thing doesn't click with his mind.

Interviewer→ So what does it mean for you to have a partner not support you? how does it make you feel?

Interviewee→ Honestly, I just see myself as a single parent, I think that thing if somehow motivates me someway to be just independent from now on. To not bug him that much but I will tell him if I need something if he doesn`t bring it (snaps fingers), I will hustle not in a bad way but in a good way (laughs).

Interviewer→ Do you think there is a relationship between partner support, your wellbeing and the baby`s wellbeing? Do you think how much support he puts in has an effect on your health? On your well-being?

Interviewee→ It damages my system physically, emotionally, it's just.... even if he calls "how are you, are you good?" it doesn`t have much effect, it's just oh you did it thank God and I go over it.

Interviewer→ Do you think it has an effect on the way you feel the fact that he is not supportive?

Interviewee→ Maybe he also contributes to my mental state [okay] since i have told you that am very slow because I think too much [okay]

Interviewer→ But how is the school work so far, the grades how are the exams going?

Interviewee→ I am managing to get sixties [where those your marks before you got pregnant] my lowest mark was... fifty percent, it was 60-60 right but my goal I wanted 70 this year.

Interviewer→ So do you feel your grades have gone down since you got pregnant?

Interviewee→ Am very weak in Economics so just rub it off [but the other courses] is very good it's like 60-69 and then...judging to my DP I got an average mark for I got 45-46 which is bad. [so, your grades have been affected] one up, one bad it's must be like steps.

Interviewer→ So you have told me you do not have a concrete plan yet for what is going to happen when the baby is born, you know you would have finished your exams, it would be during vac, so you have a few weeks...

Interviewee→ After the vac what's gonna happen [yes what's going to happen?] I do not have a plan [your mom does not have a plan] because she`ll have to go back to Joburg at some point, right?] Yes, I think that's the thing we have to discuss when she comes.

Interviewer→ Do you plan to hire someone?

Interviewee→ I do not like to hire people.

Interviewer→ So what is going to happen, are you going to drop out of school or you have a grandmother (laughs)

Interviewee→ My mother`s sister, she lives in Maritzburg [Pietermaritzburg], she did say she can take care of baby once the baby is born so that I can go back to school and study cause that is what I want to do. She was the one who volunteered to do that. Am the type of a person who believes when things are happening, if there is no action trust am just "oh you have just said that verse again". So, if your actions speak more than your words so am that type of person but I don`t think she`ll mentions thing and doesn`t do it at the end she`s the type of person if she says something she does it [okay].

Interviewer: Thank you so much for your time

Interviewee: You are welcome

Interview 10

Interviewee→ so thank you so much for taking part in this study I really appreciate. So, can you talk about your pregnancy experience? How has it been being pregnant while studying?

Interviewee→ Well for me I haven't had many difficulties cause I can balance between my school work. I was able to do all my assignment on time and everything and I passed my modules for my first semester I didn't have any... just last year when I was staring, all the morning sickness. That was the only time.

Interviewer→ okay, how far along are you?

Interviewee→ Am due this month delivering next week, I have bought baby things such as blankets.

Interviewer: So how was it? The beginning... how did you cope?

Interviewee→ gosh it was just all bad cause I was just at the hospital. I was doing my hours there so was hard and I had to study I had to sleep you know....... you just have to sleep because you are lazy. That was the only time when I was lazy

Interviewee→ And at home how has it been? Are you studying and resting?

In*terviewe*r→ I am studying at home well I am engaged to be married soon so I don't have the problem with that.

Interviewer→ So when you got pregnant or when you broke the news, did they have a problem?

Interviewee→ They did not have a problem because it is the husband anyways

Interviewer→ Is this your first pregnancy?

Interviewee→ It is not my first pregnancy, I have twins. [OH! You have twins?]

Interviewer→ You are the first person am interviewing to tell me that this is not your first pregnancy. Were you still in school with your first pregnancy?

Interviewee→ Yes, I was at school.

Interviewer→ How old are your twins?

Interviewee→ They are 4 years old.

Interviewer→ Is this pregnancy any different from the first one?

Interviewee→ Yes, it is different cause I did not reach the last trimester. I delivered at 7 months. [Was it a C-section?]. No, it was a natural birth.

Interviewer→ Are you feeling tired now that you have reached the end of this one?

Interviewee→ I am not feeling tired yet especially that my baby bump is not that big. I don't carry much, I don't have a big stomach so yeah. It's just that I didn't reach my nine months last time.

Interviewer→ Were you engaged the last time?

Interviewee→ No, I was not.

Interviewer→ How was the reaction at home?

Interviewee→ My mother noticed I was pregnant in the first month but since in our culture the charge for damages was paid, so they cooled down then we broke up, we broke up.

Interviewer→ Is he the father of your unborn baby?

Interviewee: No, he is not. [So, this one went well because of the engagement?] Yeah

Interviewee→ Apart from your own experience, what did you think pregnancy is like before you got pregnant? And did you have any unexpected experiences that you did not foresee?

Interviewee→ Not really.

Interviewer→ What do you think pregnancy should be like?

Interviewee→ Since I cannot judge people who are pregnant, so there is nothing really I can say.

Interviewer→ What am trying to ask is what do you think a normal pregnancy should be like? Like the perfect pregnancy.

Interviewee→ Just for a person whose work is to wake up and eat and not stress with school work, not having to go to school or work.

Interviewer→ How does it make you feel to be pregnant and schooling at the same time?

Interviewee→ Maybe the stigma, maybe people look at you and they talk about you and all that. At work you can't even focus, like for people who are working. Those are the difficulties they might face.

Interviewer→ What do you think causes people to stigmatise and where does this stigma come from?

Interviewer→ May be judging.

Interviewer→ Why do they judge?

Interviewee→ I think they judge because they think no...you are still at school, why should you fall pregnant?

Interviewer→ Do you sometimes feel judged?

Interviewee→ No, I do not feel judged.

Interviewer→ What about for those who are judged, do you sympathise with people who feel judged? And how do you feel about people who judge others when they are pregnant?

Interviewee→ Well, I do feel for those who are judged cause it is not good for a person to be judged because I guess a child is just some people plan it, others don't. So, it just happens.

Interviewer→ You said you are almost due next week, how has been your support system during your pregnancy and how have the people around you offered support considering your situation knowing that you are not married but engaged (you are traditionally married). Are you living with the father of your unborn baby?

Interviewee→No I am not.

Interviewer→ So, how have the people around you, your family, parents, friends, siblings and partner especially, offered support during your pregnancy?

Interviewee→ With my partner first of all he said he doesn't want me to go to clinics and all that, so he put me on his medical aid [medical insurance]. He didn't want me to be going to the clinics. There's nothing wrong with the clinic but for me he preferred the gynaecologist who is gonna do everything. With my family, it has been okay, the cravings, they understand. They do buy me whatever I crave for and my partner equally does the same and my siblings and everyone, they have started buying clothes for the baby, maybe like my 6th or 7th month. There has been a lot of support.

Interviewer→ You said your partner has put you on his medical aid, does that mean he is catering for all your medical bills? Yes [And the baby's clothes] We already bought that.

Interviewer→ Does he go with you to see the Doctor?

Interviewee→ He can't come because he stays far, he stays in North Coast. So, he doesn't come

Interviewer→ Does it make you less supportive?

Interviewee→ No I don't because I understand the situation. He can't be here because most of my appointments are on Wednesdays and he's working on those days so he can't come along with me to the doctor

Interviewer→ What is the most important type of support that you like to receive or that you would have loved to receive throughout your pregnancy? What matters the most to you?

Interviewee→ I would like to be supported emotionally because money doesn't buy love. It is the tender loving care that is most important. I appreciate that because that is what is important, that is what I prefer, it's what I need.

Interviewer→ Why do you think emotional support is the most important?

Interviewee→ I appreciate that because that is important, that is what I prefer, it's what I need.

Interviewer→ So, do you get that from your partner?

Interviewee→ Yes, I do. Well my partner is not good at giving money and all that but he can do everything and anything to support, but like I said, he put me on his medical aid, he can buy or make anything that I crave for, but giving money no. [So, he supports you?] yes

Interviewer→ How does it make you feel and what does it mean to you?

Interviewee→ It makes me feel happy and I appreciate it.

Interviewer→ So, does he support you emotionally?

Interviewee→ Yes, he does and I am satisfied with it.

Interviewer→ What about the fact that he does not give you money, does it bother you. Are there certain things that you feel you need to buy?

Interviewee→ It does not bother me a lot because I still live under my parents, my parents are the ones paying for my studies and everything, they give me petrol money and everything so I really don't mind because there are other things that he needs to do. So, what he caters for me is enough

Interviewer→ where are you going to have you baby from and will you be staying with your parents?

Interviewee→ I will be staying at home but my mother stays in Margate, so will deliver and will live there with her for two weeks.

Interviewer→ If your mother is in Margate, who is home with you?

Interviewee→ My father is there and there is a maid who will look after the baby and the father will pay her

Interviewer→ So if you deliver from home, are you going to travel with the baby?

Interviewee→ laughs yah I am gonna have to travel or my mother is gonna come it will depend how busy she is because she is the person I will need because you know you cannot be good with the baby when the baby is small.

Interviewer→ So when you say your mother is the person that you will need is the most important source of support to you.

Interviewee→ Not that she is the most with the baby whilst the baby is still little...she is with bathing the baby and all that because I am not good with doing all those things but I do need both my parents but with the father cause my father is not that kind of a person, he knows nothing about bathing the baby (yeah, I understand)

Interviewer→ so when you say your mother will be there for you after you deliver to take care of the baby in the first few days of first few weeks. Do you feel you will need her more than you do now? When do you feel you will need more support is it how is it after the baby is born?

Interviewee→ I think it is after the baby is born not financially just to help me with the baby just the first few weeks of the baby not with the money because the father will do everything any ways.

Interviewer→ so who will be the biggest source of support after the baby comes?

Interviewee→ its gonna be the father of the child.

Interviewer→ what about in terms of taking care of the baby?

Interviewee→ since I am at school there is a nanny. There is someone who will look after the child [and that nanny will be paid by?] the father of the child.

Interviewer→ so what part are you parents playing apart from your mother being there to look after the baby for you?

Interviewee→ they pay my school fees they doing what concerning me not with the baby coz I am still under their care anyways.

Interviewer→ when you think about the 9 months of pregnancy when do you feel a woman needs the most support? Do you feel it is at the beginning of the pregnancy in the middle or at the end? In your case for example when do you feel you need the most support during your pregnancy.

Interviewee→ okay the support you need is throughout your whole pregnancy.

Interviewer→ why do you say throughout the whole pregnancy?

Interviewee→ because you do get sick during the ... all the trimesters do get sick but you do need it throughout.

Interviewer→ so there is no particular time that you would want exactly the most.......

Interviewee→ At the beginning of pregnancy, maybe you are not sure what is happening to you, may be for some people it's the first time you don't know what is happening, all the sickness. At the end that's exactly what's happening to you, breaking water and all that. Some of the things you wouldn't know so someone should tell you.

Interviewer→ so when it comes to school work how has been you support system? Has there ever been a time it was difficult to balance school and being pregnant.

Interviewee→ no (okay) there hasn't, honestly. There hasn't (you have handled everything well) yes

Interviewer→ and your friends, do you have friends how has it been?

Interviewee→ as I said there hasn't because I haven't missed any lecture (okay) missing lecture is because of not coming because I didn't wanna go not because of pregnancy not because I had to go to the doctor because all my appointment are in the afternoon so yah.

Interviewer→ so how does the involvement of your partner influence the decision that you make in your pregnancy? Do you always rely on him to make decisions with you? Do you always consult him on the decision that you make?

Interviewer→ yah I have to. I do make all the decisions I make consult him I tell him.

Interviewee→ how do you think he would feel if you did not consult him.

Interviewer→ he would like that cause his way strict and traditional person so he wouldn't like it.

Interviewee→ okay so do you like this way that you have to consult him?

Interviewer→ yah (you are okay with that) yes

Interviewee→ so do you think there is a relationship between male partner involvement and mother and child health?

Interviewee→ yes it does [how so] everything that happens to me and the baby he has to know.

Interviewer→ do you feel if he wasn't supportive then things wouldn't go well with you.

Interviewee→ I will be stressed and before time and get sick and all that so he has to be there since he is the one who impregnated me he has to be there throughout [and if his not there] it can be stressful.

Interviewer→ do you know people who have gone through stress because their partners were not there.

Interviewee→ yes because some other people you tell partner in the first month and he denies it some people can even be attempted to abort the child and then they get sick. It can be stressful [okay]

Interviewer→ thank you so much for your time

Interview 11

Interviewer→ So, thank you so much for taking part in this study, i really appreciate your participation. So, we are going to talk about your support needs in pregnancy. Just feel free we just having a chat. We are talking about your pregnancy experience as a student.

Interviewee→ This is not my first pregnancy [okay] it's my second. My daughter is one year old [oh you have a one old? yah she was born last year in June [okay] I came here on campus last year i was pregnant and also this year am pregnant so a lot of people knowing me.

Interviewer→ So, what year are you?

Interviewee→ Am in 2nd year

Interviewer→ Which programme?

Interviewee→ Am in Psychology [okay that's good]

Interviewer→ And how old are you if I may ask?

Interviewee→ Am 19

Interviewer→ So last year when you had your daughter you were 18?

Interviewee→ I was 18

Interviewer→ So how has it been having two babies?

Interviewee→ It was really hard

Interviewer→ What has been your experience?

Interviewee→ I should take care of myself from now on and not get pregnant again cause this thing is really disturbing

Interviewer→ Is the father the same for the two babies

Interviewee→ yah. And his supporting really supporting

Interviewer→ So how was it let's start with the first pregnancy how was it last year?

Interviewee→ I came here maybe I was about 6 months, for 6 months when I came on campus I usually did not attend all my lectures, had to miss some of my lectures because I had to visit at the clinic to check my baby if she's okay...she was born on the 3rd she was supposed to be born on the 10th so I had to make it faster.

Interviewer→ You had a c section?

Interviewee→ Yah and I was writing on the 10th so I had to [do it quickly so that you could be in the exam room] I was studying at the ward [wow] in hospital, at night, it was really hard for me I had to study there whilst my baby is hurting inside.

Interviewer→ That was before or after the c section?

Interviewee→ Before [Before the c section and afterwards] the night before I was studying because I was going to write exams in two days...she came I gave birth to her and in two days I went home came here on campus and wrote my exams but thank God we survived [you did well in your exams] yah I really did well [that's good]

Interviewer→ What about at home how was it like the first time you got pregnant? Or even this time. First tell me about the first time then tell me about this time, what was the reaction from your parents?

Interviewee→ I live with a single mother. I don't have a father his married to another woman now.

Interviewer→ So how did your mother take it?

Interviewee→ She was okay with the first pregnancy but now this thing she`s not okay now.

Interviewer→ Has your partner paid damages for the first pregnancy.

Interviewee→ Not yet. His still organizing

Interviewer→ And how`s the thing for damages since this is the 2nd time you are carrying his child. Is it paid twice?

Interviewee→ His supposed to pay twice [he has not yet paid the first one] yah

Interviewer→ So just tell me how it was like at home from your mother when you got pregnant for the 2nd time for this pregnancy.

Interviewee→ At first, she was supportive she even supports my baby the first one even now she does may be its because she loves me. I thought she`ll kick me out of the house but she didn`t, she`s supportive.

Interviewer→ How does it make you feel to know that you have someone who is supportive, your mother is supporting you.

Interviewee→ I see that I must not disappoint her in future I must do everything right from now on because she looking after me and my sisters and brothers [are you the 1st born] yah am the 1st born they looking up to me.

Interviewer→ do you feel like it's easier going through this situation knowing that you mother is supporting you? How does is make you feel?

Interviewee→ It's not easy [it's still not easy even with her support] yah [why] because I have disappointed her so many times this is really disappointing but the baby am okay since I did not abort the baby didn`t do such things, I didn`t kill my babies I kept them it's what makes her happy because a baby is a blessing from God that's what she says she` s proud yah [she`s proud but you still feel sad that you have disappointed her] yeh

Interviewer→ But never the less how do you feel about your babies?

Interviewee→ Whoo I love them (laughs) I love them a lot, my one-year old daughter she loves me she also loves me, when am here on campus, I usually miss her sometimes I have to go home early [just to go and see her] yah

Interviewer→ What about this one, do you know what baby you are carrying?

Interviewee→ I went for a scan they told me I was too late they can`t see the gender yah

Interviewer→ So tell me what are some of the challenges you have faced in your pregnancy? both pregnancies what are some of the things you have found to be difficult that have given you stress or pressure what are some of the difficulties pregnancy comes with, in your situation when you are not married, you are living with your mother ,she`s paying for school fees, you are a student, what are the challenges?

Interviewee→ Ah sometimes I have to sleep early and not study those are some of the challenges [ah what causes you not to study] I don`t know this baby always tiring me hey it's too heavy my mother always say that I sleep too much now I have to take some treatment every day, I have to take some treatment every day, eat I eat a lot, I get hungry sometimes I bring lunch on campus have to bring more money to buy food yah sometimes I have to leave early if I feel that I have a problem with the baby inside I have to leave and go to the hospital and my appointments were usually on the day that am attending I have to go see the doctor sometimes.

Interviewer→ And how has been your school work have you grades gone up or down. How are you coping with school work?

Interviewee→ It's okay but I had...last year I had one sup and I did not write it on the first semester when I was pregnant I didn`t know that I had to come back because my baby was too small I didn`t come back to write the sup [so what happened]so I have to do it this year I have to do five modules and the sup that I missed last year [but you are coping] yah am really coping studying hard this year I don`t sleep I don`t sleep [that's good]

Interviewer→ what about at home what are the challenges you have faced considering one you are pregnant, two you already have a baby at home you are living under your mother`s roof how has it been at home we have talked about school what about at home?

Interviewee→ Am really coping at home...things are good I have a sister she`s also supporting me [how does she support you] she looks after my baby the first when i need her when

am not okay when am feeling down. And my small brother he also has a problem because he is disabled [okay] so my mother has to hire an aunty or nanny to look after the babies.

Interviewer→ So when you look at your surrounding the people around how do you think they can help you to cope better with pregnancy?

Interviewer→ Am not getting a lot of attention even with my friends I don`t have friends now [Did you have friends before] yah lived at Umlazi but now ah...moved to Pinetown so my friends I left them there.

Interviewer→ Why did you move?

Interviewer→ My mother bought a new house there in Pinetown so I left my friends there I used to chat with them on Facebook and see the pictures of the baby they were so shocked that I have a baby even now some of them don`t know that am pregnant again but my boyfriend is my friend now am always with him his really supporting.

Interviewer→ Do you feel like you need support from your friends if you were back at Umlazi?

Interviewee→ No...

Interviewer→ It's not important, okay. What`s the most important support for you?

Interviewee→ My partner [that`s the most important, that`s the number 1 support?] I think my mother [and your mother]

Interviewer→ Okay why do you say those two are the most important people you need support from?

Interviewee→ My mother she`s the one supporting us at home [supporting in terms of] ...buying us clothes doing everything for us even paying my school fees because here on campus I don`t even have bursary I pay cash. She`s the one who looks after my babies hiring a nanny to look after them and buy us food and clothes my sister and brother. And my partner...

Interviewer→ Before we go to your partner...your mother you said she`s a single mother and she`s got how many kids [3] and she`s able to support you and pay for your school fees it means that she`s well off, she`s got a good job?

Interviewee→ She`s a nurse [oh she`s a nurse] yah in Inkhosi Albert Lithuli [so it's a burden for her to be able to do all these things as a single mother] yah [it is not burden] it is because she`s not affording things that are much she`s paying school fees and buying me clothes but not a lot of clothes I have to wear small clothes and food for my brother and sister.

Interviewer→ Okay let's go to your partner why do you say you need support from your partner? why is it important to you?

Interviewee→ Because my future is with him now I don`t have anyone else I don`t want any partner beside him [why if I may ask] (laughs) I have two babies with him that's why I don`t want my baby to live with other men. He is the one who supports the baby, brings food, his always there for me every time I need him but now his not working his also a student here at Westville campus I was studying with him in high school [so you have been with him since high school] yah.

Interviewer→ If I may ask what's the age difference between you and him?

Interviewee→ His 20 now and am 19...we have one yr.

Interviewer→ What kind of support does he give you? You have said it is important to receive support from him. So what kind of support does he give you?

Interviewee→ He supports the baby the first one he buys things for her clothes, food.

Interviewer→ How does he manage considering that his also a student?

Interviewee→ He works part time [so he supports his child and you during you pregnancy how does he support you] he used to come here on campus and bring me food but not always when his free, when he doesn`t have lectures he used to come here and give it to me when an at home...he`ll bring me some food the things that i was craving for.

Interviewer→ Okay. And what about finances whose paying for your hospital bills, whose buying your baby clothes the baby that is not yet born.

Interviewee→The first one my mother had a medical aid but she cancelled it this year she paid the hospital bill everything in the hospital was paid by her the clothes and food was bought by the father.

Interviewer→ What about this pregnancy?

Interviewee→Now I think I will be giving birth in the government hospital cause last year it was a private she had a medical aid but now she cancelled it so I have to ask my partner for money [he has committed himself to] yah [...]

Interviewer→ Considering that you are 8 months into the pregnancy have you started buying things for the baby, has your partner started preparing for the arrival.

Interviewee→We are now preparing.

Interviewer→ So does it mean your mother is not going to support?

Interviewee→She is yah she is but not that much i don`t want to stress her a lot i want to do things in my own way now.

Interviewer→ So you have told me the support that you long for maybe there are things that he doesn`t do that you would like him to do. What kind of support do you want to receive from him that you are maybe not receiving or even that you are receiving. What is the most important type of support?

Interviewee→I don`t want him to treat me as his baby mother...mother of his babies i want him to treat me also his girlfriend. Now we usually talk about the baby a lot we don't talk about us.

Interviewer→ Has your relationship changed since the arrival?

Interviewee→Yah its changed but not a lot because we still love each other we used to talk about marriage how we want out future to be.

Interviewer→ Do you still talk about marriage?

Interviewee→Yah we do.

Interviewer→ So apart from you been treated as a girlfriend what other type of attitude do you want to get from him? What other type of support?

Interviewee→I don`t want him to treat me as the mother of his babies, I want him to treat me also as his girlfriend. Now we usually talk about the baby a lot. We don't talk about us. I want us to date like we used to before, go out with him, watch some movies and get a lot of support from him.

Interviewer→ So tell me when you have the baby who is going to help you? Are you going to have another c section?

Interviewer→ No [you won`t this time]

Interviewer→ So who's going to help you after the baby comes?

Interviewee→The nanny. I have to stay a few days at home and not attend, get on campus like last year [so the nanny is paid by your mother] yah by my mother.

Interviewer→ We have talked about the support that you are receiving now and the support that you need now. What about after the baby comes what would be the most important type of support that you would want to receive? Do you still want... you have said that now that you are pregnant you want that kind of support where your relationship is like fresh, where you want him to treat you like not like just a mother of his children isn`t it? What of when the baby comes what would be the most important support that you want to receive? Do you still want him to continue that or maybe there is something different? Are your needs going to change when the baby comes?

Interviewee→Yah they are going to change i want him to support my babies more than me because they are my first priority now.

Interviewer→ So what kind of support will you need then.

Interviewee→ I want him to be a father even if we have r/ship problems I still want him to support the babies and not abandon them. [support in form of] always be there for them not leave them in future even if we break up is still want him to look after the babies.

Interviewer→ When you think about your pregnancy, this is the 2nd time that you are pregnant, so obviously you have some experience with pregnancy, when you look at both of your pregnancies at what point do you require the most support? During your pregnancy is it at the beginning when find out you are pregnant, is it in the middles or it's at the end when you are about to deliver. When is the support required when do you want the most support when you ae pregnant.

Interviewee→ From the beginning to the end but the most it's at the end when you`ll be delivering.

Interviewer→ Why do you say that.

Interviewer: Mostly it's at the end when you'll be delivering (when she needs the most support) because at that time you have a lot of complications, you need someone to look after you because you can deliver anytime. You need to have transport that will take you to the hospital immediately and always bring your card so that you are well prepared.

Interviewer→ So you need more support at the end but you say that you would love it from the beginning. From the support that you have mentioned you have talked about how do you want your partner to be there for you to still take you out on dates or support the children. What is the most important type of support to you? What would you rather, if he could only give you one type of support, what would you rather have?

Interviewee→ I would rather have him as the father of my baby but not my partner if I have to choose one thing. I would rather have him as the father of my baby [why?] I don`t want my babies to suffer like I did because I don`t have a father I wasn`t raised by a male person I was only raised by a mother so I don`t want my babies to feel that.

Interviewer→ So let's talk about your health during your pregnancy does your partner ever come with you to appointments, antenatal?

Interviewee→ This time he does but last year I didn`t get time to go with him because I lived at the resident sometimes... and he also live at Kwamashu but now he lives near my house when I have to go to the doctor he always come with me, when I have to check the blood he also checks his blood, whenever am going somewhere his ever with me but not here on campus because I have to study, attend.

Interviewer→ What does it mean to you to have your partner supporting you in all these ways. How does it make you feel as a pregnant woman as a student as a mother you know?

Interviewee→ I feel so lucky because I loved him from the 1st time I knew that I will have a bright future with him so yah am really thankful for him to be always with me because there are some mothers who are single now there are some young teenagers who don`t have partners like [...] I feel grateful

Interviewer→ What do you think would happen if ...

Interviewee→ If he leaves?

Interviewer→ No if he did not support you. How would it make you feel say he was not part of the pregnancy he was not part of his daughter`s life? How would it make you feel if he was absent?

Interviewee→ I will be so sad and even my mother she would have those complications she would not feel well because I would have to have her by my side. It would be the worst thing because his the one who must be supportive always must be supportive.

Interviewer→ So do you feel your partner`s presence in your life influences the decisions you make?

Interviewee→ Yah most of the things that I do now his part of everything I must say everything because when I come here on campus I know that I have to finish my degree and work for our family when he does things he must do he must make us his priority everything that I do now it's part of his life also.

Interviewer→ Thank you so much for taking part in the interview I really appreciate.

Interview 12

Interviewer→ Thank you so much can you talk about your pregnancy experience?

Interviewee→Oh well it has its highs and lows but okay stressful but okay but it involves my family that part of course it involves my family, mom, mostly, sister at times she used to ask how it's going. So, it's been good.

Interviewer→ So that was your first pregnancy, right?

Interviewee→Yes

Interviewer→ So apart from your own experience with pregnancy what do you think pregnancy should be like for a woman? It should involve a partner, it should involve support of friends it involves a lot of family and you know how stressful and another thing I think you know just stay home just relax [...]

Interviewer→ Can you just tell me about your experience from the very beginning when you found out you were pregnant?

Interviewee→What happened to me I was shocked i couldn`t believe it was just a matter of...is this happening? Am I really...shocked, scared, happy shy just mixed emotions too many going on at the time ...just hormones were up at the same time you wanna cry but you no don`t you can`t do you can`t do this just wait till you get home and see what you gonna do when you get there.

Interviewer→ How was the reaction from your parents?

Interviewee→Well it was silence... yah it was silence cause they just didn`t know how to deal with it they never even thought such could happen. They were just shocked.

Interviewer→ Is it a kind of a situation where they believed, like they didn`t expect that from you and then it just hit them by surprise?

Interviewee→They knew the partner I was with they just never met him they knew his mom where he stayed but they just never met him. So once before in primary (sulks) high school ahm his mom called us at her place and she decided to go and tell my mom

about us and so they have known from then and hey things have been going on ever since.

Interviewer→ So what is the age difference between you and you partner? How old are you?

Interviewee→ 21 he`s like 24, about 3 years actually more maybe 3years and 3 months something like that. What does he do?

He is a stubborn man let's just call it that when he gets a job he feels like the pay is not working so he decides to quit. As if there is something better out there he will get so that`s the kinda stress he really has cause that stress has effect that for example he may be working and gets paid for example R1.600 and then just because he works Monday to Monday he will be like am not worth that and then I think to myself "You have a baby whose gonna be born you not alone you not supporting yourself alone" and am... I don`t work am still studying can`t you just push and push just for him and his like "no I can`t" and am like...that`s a stress for me that`s why am also pushing at school now for me to just school finish find a job and graduate.

Interviewer→ At what level are you in school? Level 3 [you are in your third year] yes [okay so you are almost done] yah [Do you plan to do your honours] ...maybe in a few years but right now all am looking forward right now to just lift the weight of you know lift the stress off is for me to find work for my baby.

Interviewee→ So describe some of the challenges you faced in pregnancy?

Interviewee→ Well my baby dad's drinking...for example, I'll be like I don`t have money, can you just help me out with a certain amount and he`ll just be like "okay that is enough trouble to me" and I will be like okay and then I will ask someone to help me out and then they would. So I would say other men out there are my friends and other...guys that have babies help me get though if cause all he did was drink and drink and can't you sacrifice your drinking just for me just for two one stuff and his like no am sorry I can`t sometimes he just say he has a hectic weekend where I am going to get money but the following Friday he will be drinking again and I would

be like where did you get the money to drink if you can`t give me just you know not even 100 rand, fine just a R50 and he is like I don’t have that sorry and I was like okay.

Interviewer→ So tell me a bit about your relationship with your baby father?

Interviewee→ Well we were close we spoke about everything, stupid things made up laugh we were the type of guy he is right now I don’t know.

Interviewer→ At what point did your relationship....

Interviewee→ It changed when he....when he went away that`s when it changed from there we were never the same, his that thing you know the type that he lies and forget their lies with a lie they said before so you know women are always remember we don’t forget, then defend so I was at mad at him remember what you said and what you saying now it’s like a contradiction .

Interviewer→ at what point did your relationship end?

Interviewee→ it was this ....no it was in December no it was in January.

Interviewer→ how old was your pregnancy then?

Interviewee→ I was 3 months

Interviewer→ what was the cause of the break up?

Interviewee→ all I know is that he had a girlfriend and I didn’t know he was serious with her. All of a sudden, he is like I can`t do this anymore and you are not there anymore. Am choosing, I can’t be with you any more I chose her so yah it was breaking point for me but it was the same thing that made me stronger.

Interviewer→ so what was it like going through the pregnancy without your partner by your side?

Interviewee→ well the first month it was hard cause some times you know you try to involve him and then he will just like okay cool so I was like you know what I need you know (coughs) and then there was a time where we just used to fight and fight there was

a point he started to just disrespecting and am like I can`t take this any more so I decided to just cut off all communication and that's what I did and stopped talking to him.....because even like now we don't talk at all the only time I spoke to him is if I want something from him, if he doesn't have it, okay.

Interviewer→ so at point did, if he ever did, did he buy things for the baby?

Interviewee→ I had to ask. he would have never done it if he didn't ask.

Interviewer→ but he did buy everything?

Interviewee→ After I had to beg and beg and beg and beg and him treating me like his bank, he will be treating me like I am begging him to do something he doesn`t wanna do I'll be like can we? can we not? and he be like but you know I don't have that kind of cash, you know I have to start....... and am just like you know what you not alone any more I don`t know how many times I keep telling him that because there are times whereby he doesn`t talk to me whatever how does it make me feel, how does the pregnancy make me feel how does.... I have never had anybody to talk to. I don't know how to talk to another person what's stressing me and worry you, it will be helping you by knowing and am going through something and he will be like I don't wanna worry you and I am like since you don't talk to me that makes it worse, then he didn't understand that so even if you talk he forgot. So, I stopped forcing so now if there is anything wrong with the baby, I just talk to him and we just yeah.

Interviewer→ We have talked about your challenges and your relationship what about at home what was it like with your parents?

Interviewee→ My parents there was no challenges really the only thing they just kept saying for me to push and this has really working for my baby.

Interviewer→ What about the reaction from your mom after you told her?

Interviewee→ Its my dad that I think it hurt him, and he still can`t believe it.

Interviewer→ What was his reaction?

Interviewee→I don’t know what exactly because my mom when she found out she was worried how we gonna tell my dad because my dad is the one who does everything for me he gives me money for everything.

Interviewer→ And what was her attitude towards you after she found out?

Interviewee→Well it was my mum there is no change

Interviewer→ Did she stop talking to you at any point?

Interviewee→Nah its only when I told her [And] with the day I got home it was weird, it was awkward, I felt out of place I felt like okay the ceiling is gonna crack at any time here but it didn’t. There were just it tells a situation for what it was... but one thing they were thankful for is it didn’t happen when I was younger it didn`t happen when I was still in school at least am out of school I know how it is out there fine I am a mom I won`t get how it feels just been running on myself live without having to check up on anything, getting a nanny to look after my baby and going somewhere.

Interviewer→ So you have talked about the challenges at home with your r/ship. What about at school what was it like?

Interviewee→At school I would say the only thing was sleeping I never wanted to study I was sleeping whenever I was in class I was tired. When I was at school I get hungry and I was like oh my God am so hungry I had that thing I had to carry extra cash, extra money and this so yah [and your performance at school] actually I trusted myself I just pushed myself and said God`s the only one who knows so am just gonna keep at it and keep pushing and just pushing and pushing and pushing because I don’t know tomorrow might be sunny as ever.

Interviewer→ So when you were pregnant what did you long for? What did you want the people around you to help you with in terms of support?

Interviewee→I think the best support they can do is be there don’t treat me like [...] don’t treat me as if now I am ill and you know but just know that am just a hell lot hormonal just know that otherwise don’t just say anything. For one sometimes I just used to

cry for some reason just cry myself until I fall to sleep in the morning as soon as I get up again it’s like if you haven’t slept like that and wake up and do the same thing I would be like yah.

Interviewer→ So what did you need most of the time?

Interviewee→ Just somebody to be there [be there in what way] just when I know you physically there at the same time ‘I’d feel like you emotionally there.

Interviewer→ So your baby is now about 6 weeks old his at home with your parents whose taking care of him since you are here at school?

Interviewee→He has a nanny/helper at home.

Interviewer→ So who pays the nanny?

Interviewee→My mom or my sister anybody, yah.

Interviewer→ What role does the baby`s father have in supporting for child care does he play any role?

Interviewee→His just a guy who drinks whose living his life who only cares about finance when I tell him the baby needs this then he will say, I will tell you when I have it even if he has it he tells me he doesn’t have it again. he is irresponsible I just can`t trust him at all.

Interviewer→ So let’s go back to finding out about your pregnancy what was it like you know when you just learn you are pregnant and you think about your situation you are a student, you are unemployed you are depending on your parents.

Interviewee→In a way I felt like I disappointed my parents [anything else] ... all I can say his girlfriend was back in Joburg all of the sudden he told me I am thinking of going back to Joburg.... then I think to myself how about work? you don’t have to lie to me, it’s not about work. I just kept quiet like okay because I saw lies will not get me anywhere .

Interviewer→ So you have said that when you were pregnant the most important support is just to have someone to be there for you. What about now the baby is here what about now the baby is here what support do you consider to be the most important?

Interviewee→My family, my siblings...

Interviewer→ What kind of support do they give you?

Interviewee→Everything, financially but now I know I have financial constraints of course because umm finances what I get the baby gets half and I get the other half so now I understand like that its fine he should get better more and everything.

Interviewer→ When you say what you get, what you get from where?

Interviewee→From home like financially

Interviewer→ So your allowances that your parents give you also have to share it with.

Interviewee→Men, now they just give me like half or less than half whatever and I am just like no its okay cause they are looking after him.

Interviewer→ So how does it make you feel to know that they there for you and your baby is in safe hands? What does it mean to you?

Interviewee→It's hard sometimes it's like I must finish studying ....yeah

Interviewer→ Why do you really wanna finish studying?

Interviewee→Its the support of course and at the same time to get out of home and at the same time to look after my parents too.

Interviewer→ So what do you think about male partner support during your pregnancy do you consider it to be important?

Interviewee→Its very important.

Interviewer→ Why do you say so?

Interviewee→ It helps the woman not to stress a lot to know that there is somebody he or she has a wall to lean on. Despite everything you know cause some times your family won`t support you but you know I have a partner my partner is there like something happens my partner is there 100% cause in a way I just feel like he didn't play a role he only played a role cause I initiated it I tell him you need to do this you need to do that[and if you don't tell him] cause he be like can you, can you man up like I keep saying that his exactly like his father from what he tells me his father is and that seems me it's one thing that scared me.

Interviewer→ Just tell me a little bit of what of what been going on between your family and his family concerning seeing the baby, supporting the baby?

Interviewee→ Well he has not seen the baby from day one it's just he had financial constraints so he couldn't come over, he couldn't come to the hospital but the day I went to labor he took me with and come back on his own because he couldn't stay he said this may scar me for life and I can`t stay here and I was like stay it is fine he was like this will scar me and I can`t so I was like okay.

Interviewer→ And his mother?

Interviewee→ Okay with my family and his family ahm my mom had told me that the need to come because they were not gonna go over there because he will be going to.........ahm you know my family still in the way have patriarchal thing. But mainly we can't go to females place and just since there is no male member they have to come over then my baby daddy granny said she`s not gonna do that if we don't go over there they not gonna come at all........... So I was like okay that's fine but then I told him too that since families are like this already now that's gonna hurt my daddy, it's gonna hurt you the most and you must know that if it hurts you that means you won`t see your baby for quite some time and with his mom, and my mom started meeting with his mom and ahm and spoke and asked her if she knows anything she is like yah but it's just that she is gonna make us suffer the way she suffered too if we gonna fend for ourselves so I was like okay that hurt my mom

but she is like you know what, it is fine. It's my grandson so I will raise him its fine.

Interviewer→ So how does it make you feel to know that the families are like that?

Interviewee: In a way they have always been like that it's just that now it's all worse and other thing you know there was a time whereby I asked him to tell his family as I tell mine all he told me was no I will when the time is right I am waiting for the right situation only to find out one of his friends girlfriend told me that his right situation he was making was to show that his child comes 2nd and his girlfriend1st cause he first went to his girlfriend's place to introduce themselves and whatever and she took him over to her place and he introduced her to her mom and only told his mom about the baby after before all that happened he told his girlfriend first and was like you know it is fine. He put it there clearly that we come 2nd.

Interviewer→ So from the support needs you have mentioned which one means the most to you?

Interviewee→ I would say with support [would say] if it wasn`t for my family I wouldn`t have made it. So those are the most important people to you, okay.

Interviewer→ Do you think things would have been different had your partner been involved?

Interviewee→ it could have been much easier...but now because I just told him that the baby is going out of some things he needs stuff. he`s like okay I have found something small so I will let you know when we can do shopping together am like how about you give me the money or you do it, his like no I wanna do it with you am like oh okay. Because now he thinks that money he gave me months ago it's still alive it's still supporting him right now am thinking he drinks his. Oh, he can`t think that his now not alone now has to [...]

Interviewer→ So how do you think the absence of your partner in your life influences the decisions you make concerning yourself and the baby?

Interviewee→I would say in a way it’s like am just a single parent and decide for him and the only time I get to tell is I have decided on so and so and so. At times I don`t even consider his opinion it’s just an opinion he won`t do anything about it.

Interviewer→ So you exclude him from...no...I involved him once and I saw ukuti am wasting my time he be like “I can`t talk now we`ll talk another time” like am not gonna stop this because he said so and so and so am like if you don`t wanna do this fine but just know that am not gonna call you back

Interviewer→ Do you think there is a positive r/ship between partner support and pregnancy? Do you think there is a positive r/ship, do you think you wellbeing is better if your partner is supportive.

Interviewee→Just what is it takes both male and female to do the whole process, it takes both to keep the process during the process until the end and not only from the end until after the birth and everything there has to be both.

Interviewer→ So if he is not around...

Interviewee→It’s a challenge, it’s a big challenge cause even now it’s like uhm finances he will make an issue and an issue and an issue and I will just be like Lord help me could these months just fly and at the moment he’s just like you know am just fading right now and am like I told you I don`t work I don`t have anything yet and his like “ you still studying you have got five months left and then you can fend for your baby, me nothing”. Then I think to myself in a way you don`t care whether you fend for him or not or you think of am a father that`s it. So, for me that just hurts even more but now I have realised that he just doesn`t wanna be ready and he just doesn’t care at all so its fine.

Interviewer→ Thank you so much for your time I really appreciate.

Interview 13

Interviewer→ Thank you for agreeing to take part in this study I really appreciate your time. So tell me about your pregnancy.

Interviewee→ Its so good [...] my parents are happy for me and my boyfriend. Everything is just okay. My problem is that I have become so lazy even school-wise

Interviewer→ So, this is your first pregnancy?

Interviewee→ Yes

Interviewer→ So what did you think pregnancy was like? What do you think pregnancy should be like? What would you like to experience?

Interviewee→ Once you see other girls their pregnancy is not good whilst you are at school.

Interviewer→ Why?

Interviewee→ Especially if you are at high school its better when you are at university [why is it not good] because when you deliver (speaks in Zulu).

Interviewer→ Okay I don`t understand Zulu its better if you speak English. Tell me what you find difficult in your pregnancy at school, let's start with school, is it difficult at school whilst you are pregnant?

Interviewee→ I don't study very well [you don't study well, why don`t you study well] I don`t know.

Interviewer→ How do you feel in your body?

Interviewee→ I feel good

Interviewer→ so why don't you study well.

Interviewee→ lazy... am tired

Interviewer→ How many months are you?

Interviewee→ 8 months

Interviewer→ How old are you?

Interviewee→ 20 years

Interviewer→ okay so since you say you don`t study do you pass your tests and assignment? do you do at school? How is your performance?

Interviewee→ I have submitted one of my assignment last of last week i got 60%

Interviewer→ Do you think that`s good?

Interviewee→ Not good... but its better

Interviewer→ why?

Interviewee→ It better because I [Did you always get 80 over 100, what were you getting] 70

Interviewer→ which year are you doing

Interviewee→ First year

Interviewer→ So before you used to get 70% and now you get 60% why do you think you are 60% now?

Interviewee→ I don`t know

Interviewer→ Do you think that the pregnancy has something to do with it?

Interviewee→ I think so because I sleep most of the times

Interviewer→ So what do you think people around you should do to help you?

Interviewee→ The help me with both material and emotional help.

Interviewer→ Who helps you?

Interviewee→ My mother...

Interviewer→ Where is your mother?

Interviewee→ My mother is in Jozini.

Interviewer→ And where do you live?

Interviewee→ Savannah park

Interviewer→ So your mother is far away how does it make you feel?

Interviewee→ [.....]

Interviewer→ so it`s okay you do not miss her?

Interviewee→ I miss her but.....that`s too much

Interviewer→ So talking about your mother did she react when you told her you are pregnant?

Interviewee→ She didn`t say nothing to me [she didn`t say anything, was not upset] she accepted that am pregnant she said that I must never do an abortion even if I live far from her but nothing.

Interviewer→ How does that make you feel that your mother accepted the pregnancy?

Interviewee I feel good because I was scared that my mother was going to chase me away.

Interviewer→ What about where you live who do you live with?

Interviewee→ I live with my brother and his wife.

Interviewer→ How do they treat you?

Interviewee→ They treat me very well, so you don't have any problem that you have my problem is that my brother has a baby so i don`t have the space to study from?

Interviewer→ what about the community? What do they say about the pregnancy? Do you still have as many friends as you had before you got pregnant?

Interviewee→ I don’t have many friends, they never say anything the joke around and call me mama.

Interviewer→ So tell me about your partner your boy friend does he support you?

Interviewee→ Yes

Interviewer→ What does he do?

Interviewee→ Everything cause now he gave me money to buy clothes for the baby. He gives me everything I want.

Interviewee→ So everything for the baby is ready, okay does he come with you to the clinic, what else does he do? Apart from giving you money.

Interviewer→ I live far, I am living in savannah park my boyfriend is living in Pinetown so he is too far most of the time i only go to the clinic on Tuesdays and he goes for work.

Interviewee→ is there anything else you want your boyfriend to help you with or you fine just like that.

Interviewer→ I am okay

Interviewee→ What about the money he gives you does he emotionally support you and what does he say about the pregnancy?

Interviewer→ Is he happy?

Interviewee→ He’s so happy.

Interviewer→What does he say?

Interviewee→ Many things

Interviewer→ So how do you feel knowing that your boyfriend is happy with you?

Interviewee→ I feel good

Interviewer→ Why?

Interviewee→ Because he is happy for the baby,

Interviewer: Are you looking forward of becoming a mother?

Interviewee→ Excited and looking forward to become a mother.

Interviewee→ What do you think about it?

Interviewer→ I fear the challenges of having a baby.

Interviewee→ Who's going to help you when you give birth.

Interviewer→ My mother is going to help

Interviewee→ Are you going to travel home.

Interviewee→ I will travel to home. I will be going home

Interviewer→ And what is going to happen to your school since you said that at home Jozini is far so what's going to happen to your school if you travel to Jozini.

Interviewee→ I do not know, will see what happens.

Interviewer→ How long will you go

Interviewee→ I don't know the day of labour so i don't know but I think I will be going on 12th September.

Interviewer→ And how many days are you going to go?

Interviewee→ I will come after i deliver.

Interviewer: Who is going to take care of the baby after you have given birth?

Interviewee→ I will take care of my child and we will look for the baby sitter.

Interviewer→ who is going to pay her?

Interviewee→ the baby`s father.

Interviewer→ So, how do you feel leaving the child at home whilst you are at school?

Interviewee→ I will be happy because where my mama is she`ll treat her good so I will be happy if my mother will take care of her.

Interviewer→ But how does it feel when you leave the baby at home and come to school.

Interviewee→ I think it is not going to give me any pressure.

Interviewer→ why

Interviewee→ because I will be able to take care of my child and make sure I do my academic work.

Interviewee→ so what kind of support would you want financial or emotional?

Interviewee→ both financial and emotional support

Interviewer→ now tell me how you felt when you were told you are pregnant

Interviewee→ I cried

Interviewer→ why were you crying

Interviewee→ I was scared of what my mother and father will do when the find out i was pregnant .

Interviewer→ So do you still need your boyfriend to help you after you have the baby or just now when you are pregnant.

Interviewee→ I need his support all the way because I really love him.

Interviewee→ does your r/ship affect your school life?

Interviewer→ he gives me time to do my work

Interviewee→ now you are 8 months pregnant what was the most challenging stage was it at the beginning or now.

Interviewer→ the beginning

Interviewee→ why

Interviewer→ it was because I was scared of what my parents were going to do if they found out I was pregnant. I was scared of stopping schooling.

Interviewee→ How do you feel that now you haven't stopped school.

Interviewee→ I feel great

Interviewer→ what does it mean to you, to stay in school whilst you are pregnant

Interviewee→ it gives me the courage to study.

Interviewer→ why study

Interviewee→ this is for me to get good results so that my parents will be happy.

Interviewer→ thank you very much for your time I really appreciate

Interview 14

Interviewer→ Can you talk about your pregnancy experience.

Interviewee→ Well, at first it was kind of a scary thought. It's just that I never imagined in my life me being pregnant at school, *uyabo* (you know), and all that stuff. But then as time went by, I accepted it and I was quite excited, like ok, am gonna be a mom, that is something to excite you cause in future, you have something to hold on, you know, give you a reason to wake up in the morning every day you know, I've got a mouth to feed so I really can't sleep right now- all that stuff. Yeah. But then, yeah, I've learnt to live with it, accepted it and am quite happy

Interviewer→ Is this your first pregnancy? If not how is it different from the previous one(s)

Interviewee→ Yes, it is.

Interviewer→ And how is it going so far?

Interviewee→ So far so good. I mean yeah, it's been challenging cause *uyabo,* me and my babys father broke up.

Interviewer→-At what stage of the pregnancy did that happen?

Interviewee→ At five months, actually right now am five months and three weeks. So yeah, I was like maybe four months and three weeks, or like somewhere there, so we broke up. At first, I was like heartbroken, and you know thinking, what am I gonna do with this child now? I am gonna give birth to a fatherless child and all that stuff. But then as time went by, I was like, ok, you won't be the first to be a single mom. And having my mother's support really gave me a lot of strength, like my mom is always the one asking like is she kicking? And all that stuff. Have you started the clinic? And I was like, I can do it, you know, for the sake of my baby, I have to be strong. I have to accept it. He chose to leave us, so it's up to me now, what I do.

Interviewer→ Aside from your own experience, what do you think pregnancy should be like? Describe some of the challenges you face in your pregnancy? As a student?

Interviewee→ First of all, I would say the biggest thing to me was telling my mom that am pregnant since am the only girl at home, that was like a scary thought, wow, she's gonna scream

at me, she's gonna be disappointed and all that stuff. And what happened was when I told my boyfriend that I was pregnant, he asked me to do an abortion. I was like what the hell? It's my first child, to him it's his second child so of course losing my child, I guess, he already has one so. I was like hell no. I am not gonna do it. So, we argued, I guess somehow, he adjusted like okay, fine she's not gonna do it so we continued dating but then at the end of the day he dumped me. So, telling my mom was like a scary thought but then her reaction kind of surprised me. She was so calm like ok, there's nothing surprising there, it happens these days. All you had to do was say a lot sooner so that I know the father of your baby because you know they run away.

Interviewer→-At what stage did you tell your mom?

Interviewee→ I was three months. Three to four- somewhere there.

Interviewer→ So she did no notice until you told her?

Interviewee→ She did notice cause am more like a mummy's girl (laughs). I don't sleep in my own room I sleep in her bedroom. Even when I go on my menstrual periods, if am at home, sometimes I ask her to buy for pads and she noticed like ok, this month she didn't ask and she asked me and I was like no I still have from last month. Then she was like Zinhle, you are skinny but your stomach, ah ah, and I was like no it must be something I ate. I kept making excuses but then I couldn't hide it anymore so I told her.

Interviewer→-What was her reaction?

Interviewee→ She was pretty calm. The only that I think upset her was that I didn't tell her sooner. But the rest she was calm and I was like what? I didn't expect this, if I knew I would have told her a lot sooner you know (chuckles)

Interviewer→ Where you staying home when you told her?

Interviewee→ No. I was actually here when I told her. My aunt called her and told her then she called me and we talked about it.

Interviewer→ How do you cope with being away from home?

Interviewee→ Huh It's quite a challenge, you know I miss home a lot. In Zulu some people say *uyathandiswa,* I don't know how to say it in English but most people say *ukuthandiswa ma wami (loving your mother too much) like* loving something too much cause sometimes you have cravings and all that and I always want to be with her. It's kind of difficult being away from home, you know. But then, I have to be at school, study and everything.

Interviewer→-And your school work, how are you doing?

Interviewee→ Actually am doing pretty good now. I've been having problems especially with my boyfriend but then I have adjusted and accepted it. Now it's just me and my exams and am going home.

Interviewer→ What do you think the people around you can do to help you cope with pregnancy? Is there any type of support, that you are not receiving now, that you would have lived to receive from your loved ones? Why?

Interviewee→ Actually I would have loved to have my boyfriend's support but since he is not there is nothing I can do about it. It was his choice so I had to let go.

Interviewer→-What role did you imagine he was going to play in your pregnancy?

Interviewee→ A father

Interviewer→-Do you feel you are missing out now that he is not in the picture.

Interviewee→ Yeah, I mean, besides my mom, just somebody to ask me, to hold me, feel my baby kick besides myself, you know, call me, how is the baby? You know and all that stuff. Someone to share if I go to do the scan, you know, be there with me if it's possible, or show the pictures, *uyabo,* and talk about it. It's our child of course so that kind of thing.

Interviewer→ What is the age of your pregnancy? At what stage of pregnancy do you feel you need the most support from family or friends? Why?

Interviewee→ I think I needed more support at the beginning.

Interviewer→-why?

Interviewee→ I think I needed more support at the beginning. It was something new to me. I kind of needed someone to hold my hand and say ‘it’s gonna be fine, I’m gonna be therefore you, don’t worry, we’ll get through this together. But now that he’s gone, I feel like, ok, now it’s up to you whether you continue with your life or continue living in the past, it’s up to you. The only option is to move forward, decide on your own what you are going to do, how you are going to make it. If your mom is supporting you then its fine. That’s perfect. I should live with that and I should accept it and move on.
