# Supplementary material for: Perspectives about social support among unmarried pregnant university students in South Africa
Source: PLoS One. 2023 Apr 24;18(4):e0284906. doi: 10.1371/journal.pone.0284906 (PMC10124874; doi:10.1371/journal.pone.0284906)
Supplement: Attachment — Submitted filename: PONE-D-22-16990_R1_editor comments.pdf [file pone.0284906.s003.pdf]

PLOS ONE

Perspectives about social support among unmarried pregnant university students in South Africa

--Manuscript Draft--

**Manuscript Number:**	PONE-D-22-16990R1
**Article Type:**	Research Article
**Full Title:**	Perspectives about social support among unmarried pregnant university students in South Africa
**Short Title:**	Social support among unmarried pregnant university students in South Africa.
**Corresponding Author:**	Olagoke Akintola<br>University of the Western Cape School of Public Health<br>Cape Town, SOUTH AFRICA
**Keywords:**	South Africa, Unintended pregnancy, Social support, Stressors, University students
**Abstract:**	Background: Pregnant young women in an academic environment are susceptible to stressors associated with unintended pregnancy and academic demands of universities. The challenges they face may be exacerbated by lack of social support, putting them at risk of psychological disorders such as depression. Women who receive social support during pregnancy and postpartum experience less emotional distress and report greater maternal satisfaction. However, very little is known about the perspectives about social support among unmarried pregnant students in tertiary institutions<br>Methods: Participants were purposively selected among unmarried pregnant students and those in the puerperal period at the time of the study. We conducted semi-structured qualitative interviews to explore the perspectives of unmarried pregnant students on the type of support that they need during pregnancy and the puerperium; and the period when most support is needed. The data were audio-recorded and transcribed verbatim, then analysed using thematic analysis.<br>Results: The findings show that social support (emotional, instrumental, informational, and financial) were highlighted as an important resource to cope with stressors during pregnancy and post birth. Emotional support from male partner was the most important type of support needed as it entailed a sense of being loved and cared for. Social support was identified as important throughout the different phases of pregnancy and post birth, with different support needs expressed at each of these phases.<br>Conclusion: This study identified support needs of unmarried pregnant university students in their transition to motherhood. Given the several challenges that they are faced with, unmarried pregnant students need all the available support, including male partner support to enhance wellbeing as they try to cope with academic and pregnancy-related stressors.
**Order of Authors:**	Thandiwe Msipu Phiri, Masters
	Patrick Nyamaruze, PhD
	Olagoke Akintola
**Opposed Reviewers:**	
**Response to Reviewers:**	Reviewer #1: Research ethics: It is indeed important to observe research ethics but this does not justify your statement that data cannot be shared publicly because of privacy and confidentiality issues. You can anonymize the transcripts and make them publicly available. Already you have used pseudonyms to protect the identity of participants (Line 210 and Table 1).<br>We have provided the minimal underlying data as a supporting (supplementary) information file.<br>You have correctly indicated (Line 166) that 'issues relating to pregnancy among young people are sensitive' but you have revealed the identity of the institution where data was collected (Lines 160, 161 & 163). I suggest that the name of the university be concealed.<br>Thank you for this suggestion. We have not revealed the name of the University where the study was conducted in the manuscript.

Powered by Editorial Manager® and ProduXion Manager® from Aries Systems Corporation

	Participants: The study is among unmarried pregnant university students as you clearly indicated in the title (Line 1) and some other places in the manuscript. I find it confusing when you refer to participants as 'pregnant women'; 'pregnant young women'; 'unmarried pregnant students'; young unmarried women' in Lines 46, 147, 321, 555 and at some other places. I suggest that a concept to refer to participants be identified and be used consistently. Looking at the age of the participants (Table 2), is it appropriate to refer to all of them as young women?<br>Thank you for this comment. We have chosen to refer to participants as "unmarried pregnant students" throughout the manuscript.<br>I suggest that Table 1 be moved to Results as it reflects what the study found.<br>We have moved Table 1 to the Findings section as suggested.<br>It is a good practice to describe the qualifications and experiences of researchers in a qualitative research. The description (Lines 174 - 179) of the first author (TMP) is correct, and I suggest that the other authors be described as well.<br>We have added a brief description of the qualifications and experiences of all the researchers who collaborated in the development of the manuscript. For example: "OA is a Public Health Promotion and Research Methods specialist" and "PN; a post-doctoral fellow and researcher".<br>Language: Replace Master's (Lines 175 - 177) with Masters.<br>We have made the correction and replaced Master's with Masters.<br>School (Lines 242, 482) should be replaced with university.<br>Thank you for this correction. We have used the term "university" in line 245 and "tuition" in line 485.<br>The manner of writing ANC (Lines 527, 536 and 551) should rectified. Write in full with abbreviation in brackets on first time use, then abbreviation thereafter.<br>We have rectified this error and also rectified in the different sections of the manuscript.<br>The concept 'clinics' (Lines 536 - 538) needs to be clarified as specialists run clinics in both private and government/public health services.<br>Thank you for this suggestion. We have clarified the concept "clinics" and explained that, "Some participants explained that they were not attending ANC in public clinics but instead were consulting specialists like gynaecologists and obstetricians in private hospitals to monitor their pregnancy. This was mostly because they felt that they needed to be checked by specialists who were not readily available in public clinics".<br>Lines 551 - 557 regarding ANC need to be rephrased as ANC is available through private and public health facilities.<br>We have revised this section and removed some material as it was not relevant. This section now reads: "Overall, it was established that most unmarried pregnant students seek ANC in public clinics and hospitals alone without the support of their male partners. The participants generally considered ANC to be unfriendly. In addition, partner attendance was not specifically encouraged or recommended by healthcare workers".<br>Furthermore, pregnant women can deliver in a public hospital even when they did not attend ANC at a public/government facility or even when they did not attend ANC at all. Therefore, Lines 668 - 669 should be revised.<br>Thank you for this correction. We have amended the section and explained that, "The South Africa Department of Health guidelines recommend that pregnant women seek ANC as soon as they suspect a pregnancy and encourages all pregnant women to attend at least eight ANC visits".<br>Line 553 - 554 contradicts itself regarding affordability of private healthcare (none & except), see also Lines 670 - 671 regarding ability to pay for private healthcare.<br>We have removed this contradicting sentence.<br>Pregnancy and conception (Line 639) should be conception and pregnancy.<br>Thank you for this correction, we have amended to highlight the suggested change.<br><br>Description of study design (Lines 150 - 158) should be rephrased to clarify if qualitative research is a design or a method. Based on what you wrote in Line 157, the appropriate design appears to be a narrative design or even a phenomenological design might be suitable.<br>We have rephrased in the manuscript and included "descriptive" qualitative research design. There are a number of researchers who believe and support the fact that 'descriptive qualitative research design' is a viable and acceptable research design (Doyle et al., 2020; Lambert & Lambert, 2012; Ulin et al., 2005). This design is an acceptable research design for robust scholarly research and has received varying degrees of acceptance within the academic community. In this study we use the

Powered by Editorial Manager® and ProduXion Manager® from Aries Systems Corporation

descriptive qualitative research as a design in its own right. The most frequently proposed rationale for the use of a descriptive design is to provide straightforward descriptions of experiences and perceptions (Sandelowski, 2000), particularly in areas where little is known about the topic under study.

Doyle, L., McCabe, C., Keogh, B., Brady, A., & McCann, M. (2020). An overview of the qualitative descriptive design within nursing research. Journal of Research in Nursing, 25(5), 443-455.
Lambert, V. A., & Lambert, C. E. (2012). Qualitative descriptive research: An acceptable design. Pacific Rim International Journal of Nursing Research, 16(4), 255-256.
Ulin, PR, Robinson, ET, & Tolley, EE (2005). Qualitative methods in public health: A field guide for applied research. Jossey-Bass.
Sandelowski M. Whatever happened to qualitative description?. Research in nursing & health. 2000;23(4):334-40.

Indicate under Data collection if field notes were collected or not as they are important to add to the context of the interviews.
We have explained that, “The interviewer (TMP) made field notes during the interview and then summarized these following the interview. The field notes were thereafter used to develop analytical memos.”.

Data analysis: Indicate (Line 209) that data was transcribed verbatim. This is only indicated in the Abstract (Line 39), but it is important to indicate this in the main document.
We have highlighted in the manuscript that data was transcribed verbatim. The sentence now reads, “The audio-recorded data was transcribed verbatim by the first author (TMP)”.
Results: Indicate the number of sub-themes in Line 229.
We have indicated the number of sub-themes. “The findings are organized into four main themes and ten sub-themes identified from the analysis of the data”.
Line 239 should say sub-themes, not themes. Clarify also 'four main subthemes arose
We have amended to read, “The following three sub-themes emerged from the interviews.”.
(Line 334). Is the word 'main' necessary?
We have removed the word.
The following concepts [many of the participants; several participants; most participants; a few of the participants] are not appropriate to report qualitative findings as they suggest numbers; appropriate concepts are some participates, other participants and others. Writing just 'participants' may also imply that it is all participants who shared that experience. I found both appropriate and inappropriate concepts in this manuscript and suggest that the inappropriate concepts be replaced.
Thank you for this comment. We have used the suggested qualifiers in some places where possible, but in some instances, we felt that some of the qualifiers we had used were the most appropriate way to convey the general experiences of the participants in relation to a particular phenomenon.
Line 311 should acknowledge that some participants had previous pregnancies and therefore knew what to expect.
We have amended this section to read, “One of the participants who had a previous pregnancy explained the difficulties of having a first pregnancy without adequate knowledge of the accompanying physical changes”.
Lines 319 - 321 and 330 - 331 appears to be misplaced; they would suit the Discussion section.
We have removed the sentence in line 319-321. In line 330-331 we have amended the sentence to show our interpretation of the participant’s experience. The sentence now reads, “The participant’s narrative highlights the challenges of poor nutritional intake during pregnancy”.

Context: It is important to mention if these pregnancies were planned or not, if participants disclosed their pregnancies to whoever they expected to give social support and whether they are still in a continuing and healthy relationships with the partner who impregnated them. Where (on-campus or off-campus) and with whom were participants staying? Lines 599, 606-607 are not clear regarding where participants were staying.
In the design and data collection of the study we did not specifically seek to explore

	information on the nature of the pregnancies (planned or unplanned); whether they were staying on-campus or off-campus; and with whom they were staying. However, some of this information emerged during the course of our interviews and hence it is reflected in some of the narratives. In instances where this information emerges we attempt to provide some context. Pregnancies were disclosed in all cases to significant others who were expected to offer social support and we have highlighted this in the manuscript.<br>Discussion: Lines 590 and 648-649 give an impression that types of social support were rated in this study. Rating is a quantitative concept.<br>The study sought to explore the social support needs of unmarried pregnant students in tertiary institutions during pregnancy and the puerperal period, and provide insight into the types of social support that were most important to them (primary source of support). However, we have revised the language such that it does not reflect quantitative concepts:<br>“Although a previous study reported on the importance of emotional support during pregnancy, it was not rated regarded as the most important type of support needed”.<br>“Partners’ instrumental and financial supports were also considered to be important sources of support”.<br>Lines 653-656 should say some African cultures because in some communities in South Africa, paying damages means admission that one has impregnated a woman by paying a fine and not a commitment to support the child financially (paying damages is a once-off payment).<br>Thank you for this correction. We have specified that: “In some African cultures, payment of damages is perceived as a form of acceptance of paternity. This process was perceived by the participants to give a form of dignity to her family and some assurance that the father was prepared to take care of the child”.<br><br>Recommendation: It is not clear on which findings are the recommendations on behaviour change programme (Line 688) and rights-based approach (Line 639) based.<br>In the manuscript we explain that the participants mentioned that some male partners were not usually willing to help them with tasks. Experiences of social stigma were also reported at public health facilities. Hence, to overcome some of these challenges’ interventions informed by behaviour change and rights-based approach are essential. We explain in the manuscript that:<br>“Absence or lack of emotional social support from significant others, particularly partners may exacerbate the stressors of pregnancy among students. Tertiary institutions should partner with established social initiatives offering services for men in the country to engage and mentor young men one-on-one and in groups about the importance of the father’s role in the child’s development and how to best support their partners to improve pregnancy outcomes, irrespective of relationship status. Culturally sensitive health promotion programmes targeted at ‘father inclusive’ practices and behaviour change communication programmes to stimulate social support may have positive changes in terms of the supportive role of partners”.<br>“Male involvement programmes can be integrated in ANC whereby services are restructured to accommodate male partners to ensure a sense of involvement and responsibility. However, for these programmes to work, the ANC environment should be user friendly for young women and their partners. A rights-based approach must be consistently adopted when delivering maternal healthcare. Training and on-going education should be prioritised for healthcare workers regarding the provision of positive, respectful and supportive non-judgmental antenatal and postnatal care to their clients irrespective of their age or marital status”.<br>Who are the 'antenatal health personnel' in Line 695?<br>We have amended the sentence and explained that: “Training and on-going education should be prioritised for healthcare workers regarding the provision of positive, respectful and supportive non-judgmental antenatal and postnatal care to their clients irrespective of their age or marital status.”<br>Reviewer #2: There is need to rephrase the topic to experiences of unmarried pregnant students on male partner social support to speak to the background of the study.<br>Thank you for this comment. We have revised the background to highlight that the main issue of concern is social support needs for unmarried pregnant students and not only male partner social support. While we acknowledge that male social support is one of the main themes that emerged in the findings, the focus of the study is social support in general. However, we have rephrased the topic to, “Perspectives about social support among unmarried pregnant university students in South Africa”.

	On abstract the section of methods line 35, the candidates say perceptions there must be consistency in usage of words, what is explored are experiences.<br>We have rephrased to clearly highlight that the study is exploring perspectives about social support among unmarried pregnant students in a university setting and not experiences.<br>40-41 the results should be reported in line with the study aim which is pregnant women support needs and when it was needed the most.<br>We have revised this section to highlight the aim of the study which is, "to explore the perspectives of social support among unmarried students in a university setting during pregnancy and the puerperium; and the period when most support is needed". The results are reported in line with this study aim.<br>Under line 150, it should be clear which research design under qualitative research approach was employed that informed the thematic content analysis.<br>We have rephrased in the manuscript and included "descriptive" qualitative research design. There are a number of researchers who believe and support the fact that 'descriptive qualitative research design' is a viable and acceptable research design (Doyle et al., 2020; Lambert & Lambert, 2012; Ulin et al., 2005). This design is an acceptable research design for robust scholarly research and has received varying degrees of acceptance within the academic community. In this study we use the descriptive qualitative research as a design in its own right. The most frequently proposed rationale for the use of a descriptive design is to provide straightforward descriptions of experiences and perceptions (Sandelowski, 2000), particularly in areas where little is known about the topic under study. In this type of design, data analysis "does not use a pre-existing set of rules that have been generated from the philosophical or epistemological stance of the discipline that created the specific qualitative research approach. Rather, qualitative descriptive research is purely data-derived in that codes are generated from the data in the course of the study" (Lambert & Lambert, 2012, p.256). Thus, we have further explained in the manuscript that. "As qualitative descriptive research is purely data-derived, we specifically employed an inductive approach to thematic analysis".<br><br>Under 171 how many students were snowballed were in puerperal period.<br>Among the first group of students who were approached, all of them were pregnant. None of them were in the puerperal period. However, through snowball sampling we were able to identify these students who had recently given birth. Essentially, all of the participants in the puerperal period were recruited through snowball. In the manuscript we explain that: "The first author (TMP) initially approached three pregnant young students who stayed in the same residence as her. She (TMP) discussed about the study with those young pregnant students and requested them to identify and recommend other students who were pregnant or had recently given birth".<br>Line 145 should specify the experiences from unmarried pregnant students are about "male social support".<br>We have explained earlier on that the study was not specifically focusing on "male partner support" but rather support needs of unmarried pregnant students. We have explained in the manuscript that, "Therefore, the aim of this study is to explore the perspectives about social support among unmarried pregnant students in a university setting".<br>119-144 provides an impression that the social support explored is only from male partners. Line 147 emphasis the said point which contradicts the study topic.<br>We have revised this section and deleted some material which suggested that the social support explored is only from male partners.<br><br>On line 197 the interview indicates that the schedule elicited the support needs of pregnant students and the period when most support is needed. The results should be aligned to what the interview guide elicited<br>We have revised this section to include all the topics covered in the interview schedule. This section now reads, "The questions contained in the interview schedule included the socio-demographic variables of participants and their partners, the support needs of pregnant students, the period when most support is needed and types of male partner support needed".<br>on line 149, the section on study objectives should be included.<br>We have included the objectives of the study:<br>1) To explore the social support needs of unmarried students in tertiary institutions during pregnancy and the puerperium.

Powered by Editorial Manager® and ProduXion Manager® from Aries Systems Corporation

	2) To explore the period when most support is needed by unmarried students in tertiary institutions during pregnancy and the puerperium.<br><br>line 171-173, how many peripheral students were identified, only pregnant student are mentioned.<br>Among the first group of students who were approached none of them were in puerperal period and we only managed to gather these participants in the puerperal period through referral. We have explained that: “The first author (TMP) initially approached three pregnant young students who stayed in the same residence as her. She (TMP) discussed about the study with those young pregnant students and requested them to identify and recommend other students who were pregnant or had recently given birth”.<br><br>Under 189, what ethical codes were considered in this study and the study is sensitive in nature, how was the emotional risk addressed.<br>We explained that: “Data collection was carried out in accordance with relevant guidelines and the participants could withdraw from the study at any point. Although the study was of a sensitive nature, we did not anticipate any negative effects on the participants. However, we had arranged counselling services for the participants if the need arises but none of the participants requested for these services”.<br>Line 205, What was used to record data and where was data stored.<br>We have explained that: “We used a digital audio voice recorder and the recordings were safely stored in a password protected computer”.<br>Under 226, I suggest authors remove the social support needs from the general social network. That is, the first section of social support needs can be deleted, and the results presents the social support needs from participants male partners and the period they needed the support in order to align with the strong assertions made in the background.<br>Thank you for this suggestion. We have revised the background to highlight that the study will explore social support needs of unmarried pregnant students and not primarily focus on the social support needs from participants male partners. We believe that removing the social support needs from the general social network will not do justice to the study as these are some of the main issues that were expressed by the participants.<br><br>558 most participants are undergraduates and what implications does this have to the present study. The socio-economic background of participants is something that could have been explored from the participants<br>We did not specifically seek to explore the influence of the socio-economic background of participants nor did we aim to understand the implications of the level of study on the experiences of the participants. We acknowledge that these aspects could have provided a different perspective and insight to the study’s findings. However, there are sections where we elaborate on the influence of the participants’ socio-economic background. For example, “The baby needs so many things so as to grow in a good state; nappies, warm clothes, milk, formula [baby food] since am not going to breastfeed so financially it will be much required from him. [She gives an example]-cause my cousin gave birth in April and the father of the baby hasn’t given her a single cent since she gave birth, since she fell pregnant, she hasn’t received any money from the baby’s daddy and telling me things are difficult, so financially it is so important”(pg. 20, line 438-443); “Lastly, financial support was needed to buy food, to cover transport costs and medical expenses and to prepare for the arrival of the unborn baby” (pg. 28, ;line 611-613).<br>What recommendations can be made to deal with students’ emotional risk that the lack of male partner support impose on unmarried pregnant students. Additionally, what strategies can be recommended to cultivate a culture of male social support towards their female pregnant partners.<br>Thank you for highlighting these issues. As part of the recommendations we have added that, “In instances where emotional support from their male partners is not feasible, unmarried pregnant students need to identify alternate sources of support such as counselling support, friends, church and social media”.<br>“Tertiary institutions should partner with established social initiatives offering services for men in the country to engage and mentor young men one-on-one and in groups about the importance of the father’s role in the child’s development and how to best support their partners to improve pregnancy outcomes, irrespective of relationship

Powered by Editorial Manager® and ProduXion Manager® from Aries Systems Corporation

	status. Culturally sensitive health promotion programmes targeted at 'father inclusive' practices and behaviour change communication programmes to stimulate social support may have positive changes in terms of the supportive role of partners. Implementors of such programmes should consider adopting innovative outreach strategies that appeal to young men's interests and hang-out spots, such as bars and sports arenas".
Additional Information:	
Question	Response
**Financial Disclosure**<br><br>Enter a financial disclosure statement that describes the sources of funding for the work included in this submission. Review the submission guidelines for detailed requirements. View published research articles from *PLOS ONE* for specific examples.<br><br>This statement is required for submission and **will appear in the published article** if the submission is accepted. Please make sure it is accurate.<br><br>**Unfunded studies**<br>Enter: *The author(s) received no specific funding for this work.*<br><br>**Funded studies**<br>Enter a statement with the following details:<br>• Initials of the authors who received each award<br>• Grant numbers awarded to each author<br>• The full name of each funder<br>• URL of each funder website<br>• Did the sponsors or funders play any role in the study design, data collection and analysis, decision to publish, or preparation of the manuscript?<br>• **NO** - Include this sentence at the end of your statement: *The funders had no role in study design, data collection and analysis, decision to publish, or preparation of the manuscript.*<br>• **YES** - Specify the role(s) played.<br><br>* typeset	The authors received no specific funding for this work.
**Competing Interests**<br><br>Use the instructions below to enter a competing interest statement for this submission. On behalf of all authors, disclose any competing interests that	The authors have declared that no competing interests exist.

could be perceived to bias this work—acknowledging all financial support and any other relevant financial or non-financial competing interests.<br><br>This statement is required for submission and **will appear in the published article** if the submission is accepted. Please make sure it is accurate and that any funding sources listed in your Funding Information later in the submission form are also declared in your Financial Disclosure statement.<br><br>View published research articles from *PLOS ONE* for specific examples.<br><br>**NO authors have competing interests**<br><br>Enter: *The authors have declared that no competing interests exist.*<br><br>**Authors with competing interests**<br><br>Enter competing interest details beginning with this statement:<br><br>*I have read the journal's policy and the authors of this manuscript have the following competing interests: [insert competing interests here]*<br><br>* typeset	
**Ethics Statement**<br><br>Enter an ethics statement for this submission. This statement is required if the study involved:<br><br>• Human participants<br>• Human specimens or tissue<br>• Vertebrate animals or cephalopods<br>• Vertebrate embryos or tissues<br>• Field research<br><br>Write "N/A" if the submission does not require an ethics statement.	This study was approved by the University of KwaZulu Natal Humanities and Social Sciences Research Ethics Committee in Durban (ref no; HSS/0584/015M). Written consent was sought and provided by all the participants. Participation was voluntary and the participants could withdraw from the study at any point. Pseudonyms are used to identify the illustrative quotes by the participants and to ensure that their confidentiality is maintained. We confirm that all methods were carried out in accordance with relevant guidelines and regulations.

Powered by Editorial Manager® and ProduXion Manager® from Aries Systems Corporation

General guidance is provided below. Consult the submission guidelines for detailed instructions. **Make sure that all information entered here is included in the Methods section of the manuscript.**<br><br>**Format for specific study types**<br><br>**Human Subject Research (involving human participants and/or tissue)**<br>• Give the name of the institutional review board or ethics committee that approved the study<br>• Include the approval number and/or a statement indicating approval of this research<br>• Indicate the form of consent obtained (written/oral) or the reason that consent was not obtained (e.g. the data were analyzed anonymously)<br><br>**Animal Research (involving vertebrate animals, embryos or tissues)**<br>• Provide the name of the Institutional Animal Care and Use Committee (IACUC) or other relevant ethics board that reviewed the study protocol, and indicate whether they approved this research or granted a formal waiver of ethical approval<br>• Include an approval number if one was obtained<br>• If the study involved *non-human primates*, add *additional details* about animal welfare and steps taken to ameliorate suffering<br>• If anesthesia, euthanasia, or any kind of animal sacrifice is part of the study, include briefly which substances and/or methods were applied<br><br>**Field Research**<br><br>Include the following details if this study involves the collection of plant, animal, or other materials from a natural setting:<br>• Field permit number<br>• Name of the institution or relevant body that granted permission	
**Data Availability**<br><br>Authors are required to make all data	Yes - all data are fully available without restriction

Powered by Editorial Manager® and ProduXion Manager® from Aries Systems Corporation

underlying the findings described fully available, without restriction, and from the time of publication. PLOS allows rare exceptions to address legal and ethical concerns. See the PLOS Data Policy and FAQ for detailed information.<br><br>A Data Availability Statement describing where the data can be found is required at submission. Your answers to this question constitute the Data Availability Statement and **will be published in the article**, if accepted.<br><br>**Important:** Stating 'data available on request from the author' is not sufficient. If your data are only available upon request, select 'No' for the first question and explain your exceptional situation in the text box.<br><br>Do the authors confirm that all data underlying the findings described in their manuscript are fully available without restriction?	
**Describe where the data may be found in full sentences. If you are copying our sample text, replace any instances of XXX with the appropriate details.**<br><br>• If the data are **held or will be held in a public repository**, include URLs, accession numbers or DOIs. If this information will only be available after acceptance, indicate this by ticking the box below. For example: *All XXX files are available from the XXX database (accession number(s) XXX, XXX.*).<br>• If the data are all contained **within the manuscript and/or Supporting Information files**, enter the following: *All relevant data are within the manuscript and its Supporting Information files.*<br>• If neither of these applies but you are able to provide **details of access elsewhere**, with or without limitations, please do so. For example:<br><br>*Data cannot be shared publicly because of [XXX]. Data are available from the*	All relevant data are within the manuscript and its Supporting Information files.

XXX Institutional Data Access / Ethics Committee (contact via XXX) for researchers who meet the criteria for access to confidential data.<br><br>*The data underlying the results presented in the study are available from (include the name of the third party and contact information or URL).*<br>• This text is appropriate if the data are owned by a third party and authors do not have permission to share the data.<br><br>* typeset	
Additional data availability information:	

Powered by Editorial Manager® and ProduXion Manager® from Aries Systems Corporation

Dear Editor

PLOS ONE

Re: Perspectives about social support among unmarried pregnant university students in South Africa.

We are submitting our research article "Perspectives about social support among unmarried pregnant university students in South Africa" to your esteemed journal *PLOS ONE* for consideration.

There is very little attention focused on students in institutions of higher learning in studies on pregnancy and social support. However, the reality is that young women and university students in particular are a vulnerable group considering the circumstances that surround their pregnancy such as their age, unstable relationships with their baby's fathers, risk of HIV infection due to the sexual culture in universities, unemployment/lack of income and academic commitments. While a lot of students choose to terminate their unplanned pregnancies, those that choose to keep their pregnancies remain in need of a supportive environment that favours their psychological wellbeing. Therefore, it seems reasonable to suggest that pregnancy is a period when women need a support system to help them cope with the changes as well as preparation for their impending role of motherhood. Findings from this study will provide insight into the support needs of unmarried pregnant students. This is the first time we are submitting our manuscript.

We have provided the minimal underlying data as a supporting (supplementary) information file and we hope that our article will be favourably considered for publication in your journal.

Yours sincerely

Olagoke Akintola (PhD)

Suggested appropriate Academic Editors:

Tanya Doherty

Dell Elizabeth Horey

Godwin Otuodichinma Akaba

Hamideh Bayrampour

Manuscript Click here to access/download;Manuscript;Manuscript.docx

Perspectives about social support among unmarried pregnant university students in South Africa

Thandiwe Msipu Phiri[a], Patrick Nyamaruze[b], Olagoke Akintola[c]*

[a]Discipline of Psychology, School of Applied Human Sciences, University of KwaZulu-Natal, Durban, South Africa.

[b]School of Public Health, Faculty of Community and Health Sciences, University of the Western Cape, South Africa

[c]School of Public Health, Faculty of Community and Health Sciences, University of the Western Cape, South Africa

*Corresponding author: oakintola@uwc.ac.za (OA)

Abstract

Background: Pregnant young women in an academic environment are susceptible to stressors associated with unintended pregnancy and academic demands of universities. The challenges they face may be exacerbated by lack of social support, putting them at risk of psychological disorders such as depression. Women who receive social support during pregnancy and postpartum experience less emotional distress and report greater maternal satisfaction. However, very little is known about the perspectives about social support among unmarried pregnant students in tertiary institutions

Methods: Participants were purposively selected among unmarried pregnant students and those in the puerperal period at the time of the study. We conducted semi-structured qualitative interviews to explore the perspectives of unmarried pregnant students on the type of support that they need during pregnancy and the puerperium; and the period when most support is needed. The data were audio-recorded and transcribed verbatim, then analysed using thematic analysis.

Results: The findings show that social support (emotional, instrumental, informational, and financial) were highlighted as an important resource to cope with stressors during pregnancy and post birth. Emotional support from male partner was the most important type of support needed as it entailed a sense of being loved and cared for. Social support was identified as important throughout the different phases of pregnancy and post birth, with different support needs expressed at each of these phases.

Conclusion: This study identified support needs of unmarried pregnant university students in their transition to motherhood. Given the several challenges that they are faced with, unmarried pregnant students need all the available support, including male partner support to enhance wellbeing as they try to cope with academic and pregnancy-related stressors.

Background

Pregnancy marks the beginning of the transition to motherhood, and it is a period of great physical and psychological changes for all women [1]. For a lot of women, it is a period of time that is spent in material and psychological preparation for the arrival of a baby, whose presence brings about several changes in the mother's life. But for some women, this can be a period characterised by considerable physical and emotional stress. The stressors range from physical exhaustion, overwhelming tasks, decreased financial resources, social isolation and depressive symptoms [1, 2]. Research shows that young women experience several stressors during pregnancy due to their age and social context. Stressors such as academic demands, lack of income and relationship problems with their partners could affect their psychological wellbeing [3, 4]. Therefore, it seems reasonable to suggest that pregnancy is a period when women need a support system to help them cope with the changes as well as preparation for their impending role of motherhood

Although social support is of great value for all mothers during pregnancy, certain groups of women are especially vulnerable during pregnancy, and these include adolescents, unmarried women, students and women of low socio-economic status [5]. Because of the risky sexual behavior among students, young women in institutions of higher learning have a high risk of unintended pregnancy [6]. There is very little attention focused on students in institutions of higher learning in studies on pregnancy and social support. This could be because they are considered as 'advantaged' as compared to women with low educational attainment [7]. However, the reality is that young women and university students in particular are a vulnerable group considering the circumstances that surround their pregnancy such as their age, unstable relationships with their baby's fathers, risk of HIV infection due to the sexual

culture in universities, unemployment/lack of income, family dependency, academic commitments and having to deal with pregnancy in an academic environment [8-10].

University students are a population that is susceptible to stress given their academic demands [11]. Most university students are at an age and social context where they are transitioning from adolescence into young adulthood. This can be a difficult period particularly for first year students who struggle with fitting in, maintaining relationships and getting good grades amid the growing demands of life at the university [12].

Pregnancy among students may also carry social stigma and has been described as a traumatic event for a lot of unmarried young women [13]. Further, some young women experience stress due to the instability of their relationships with their partners [8]. Facing these challenges in the midst of academic demands may be daunting. While a lot of students choose to terminate their unplanned pregnancies [14], those that choose to keep their pregnancies remain in need of a supportive environment that favours their psychological wellbeing. Young mothers have less support and face more challenges in terms of adjustment to motherhood as compared to older women [15]. For young mothers in an academic environment, the integration of their roles as students with their new role of motherhood can be confusing and stressful [16]. The challenges they face may be exacerbated by lack of social support, putting them at risk of psychological disorders such as postpartum depression. Furthermore, one study showed that married women received more support from their partners as compared to unmarried women [17]. This shows that marital status influences the amount of support that pregnant women receive from their partners. Women who receive support from their partners both during pregnancy and in the postpartum period have been shown to experience better mental health outcomes including less anxiety, emotional distress, depressive symptoms and report greater maternal satisfaction [18, 19].

Social support for unmarried pregnant women can come from different sources among them family, friends, male partners and their community. House [20] defines social support as perception and actuality that one is cared for, has assistance available from other people, and that one is part of a supportive social network. Social support, either received from people closest to a person or offered through organizational interventions has a positive influence on pregnancy experiences [21].

Social support is a multi-dimensional construct and has been conceptually divided into four domains: informational, instrumental, appraisal, and emotional support [22]. Emotional support includes any actions of caring towards and improvement of esteem in the recipient of the support. Emotional support for pregnant women could decrease the likelihood of psychological stress disorders and depressive symptoms. Informational support is generally advice and guidance given and, in this case, it could positively influence decisions on issues such as prenatal care, recommended nutritional and healthcare practices and preparation for labour and delivery [22]. Instrumental support involves giving help with tasks or providing material or tangible goods to an individual [22]. Instrumental support such as assistance with household chores and childcare could help expectant mothers cope with physical exhaustion and physically taxing demands which could cause strain, particularly in the last trimester of the pregnancy [23]. Appraisal support is providing evaluative feedback to others and consistent positive feedback during pregnancy and after birth may help normalise the concerns of mothers and build their self-esteem and self-confidence in their parenting role [24]. Support networks have a buffering effect on the stressors associated with pregnancy and motherhood [25]. In the context of maternal and child health, supportive relationships may have a positive effect on pregnant women, giving them a sense of personal control and enhanced feelings of wellbeing that would help them perceive their pregnancy as less stressful [25, 26].

Unmarried pregnant students are a vulnerable group considering the circumstances that surround their pregnancy such as their age, unstable relationships with their baby's fathers, unemployment/lack of income, academic commitments and social stigma. However, the support needs of unmarried pregnant students in university settings has received little attention. Therefore, the aim of this study is to explore the perspectives about social support among unmarried pregnant students in a university setting. The findings could provide insight into factors that could assist in designing interventions for this population.

Objective

We sought to achieve two objectives in the study:

1) To explore the social support needs of unmarried students in tertiary institutions during pregnancy and the puerperium.

2) To explore the period when most support is needed by unmarried students in tertiary institutions during pregnancy and the puerperium.

Methods

Study design

The descriptive qualitative research design was seen as best suited to answer the research questions in this study. Descriptive qualitative research design enables researchers to get an insider's view of participants and their experiences in a natural setting [27]. The rationale for the use of a descriptive qualitative research design is to provide straightforward descriptions of experiences and perceptions [28], particularly in areas where little is known about the phenomenon under investigation. The choice of a descriptive qualitative research design was primarily determined by the type of questions the study sought to address, that is, to gain a

deep understanding of the type of social support needed by unmarried pregnant students. This design was appropriate as it fostered maximum co-operation and closeness between the interviewer and the participants and enabled them to describe their perspectives about pregnancy and support needs while studying in the University.

Participants

Participants in the study were all female students at a public University in South Africa. Participants were recruited if they met a set of inclusion criteria which included that they: were currently registered as students at the University; were currently pregnant or in the puerperal period (less than six weeks post childbirth) at the time of the study; self-identified as being unmarried; and were willing to answer questions regarding their perspectives about their pregnancy and support needs.

Because issues relating to pregnancy among young people are sensitive and therefore not openly discussed, it was not easy to recruit participants for the study. Therefore, we used the snowball sampling technique in this study. This is a type of purposive sampling that allowed participants to recommend others in their circle who met the inclusion criteria and were willing to participate in the study [29].

The first author (TMP) initially approached three unmarried pregnant students who stayed in the same residence as her. She (TMP) discussed about the study with those students and requested them to identify and recommend other students who were unmarried and pregnant or had recently given birth. Next, she (TMP) approached and invited those who met the criteria to participate in the study. The first author was a Masters student and a young woman who had a pregnancy and delivered a baby while studying for her Masters degree. She had received training on qualitative interviewing as part of her Masters degree and additional training from the third author (OA) who is a Public Health Promotion and Research Methods

specialist. She conducted all the interviews drawing on her own pregnancy experiences. The rationale for that was that some of the participants might be ashamed to express themselves when being interviewed by the opposite sex or an individual who had not experienced pregnancy. It has been shown that the gender of the interviewer can substantially affect the response rate in data collection [30].

Thirty-three students were invited to participate in the study and seven of them were excluded because they were past the puerperal period. Two of those invited to participate mentioned that they were not comfortable to discuss their pregnancy experiences and the remaining 24 were recruited to participate in the study.

Data collection

Ethical approval for the study was obtained from the University of KwaZulu-Natal Humanities and Social Sciences Research Ethics Committee in Durban (HSS/0584/015M). In-depth interviews were conducted using an interview schedule which contained open-ended questions. The interview schedule was designed to suit the context of the study setting and to encourage unmarried pregnant students to discuss their experiences. The schedule was also reviewed and approved by the third author (OA) who was the research project supervisor. The questions contained in the interview schedule included the socio-demographic variables of participants and their partners, the support needs of unmarried pregnant students, the period when most support is needed and the role of their male partners.

The interviews were conducted in a private and quiet seminar room within the university. This gave the participants an atmosphere of safety and comfort in which they could talk about their personal experiences considering the sensitive nature of the topic. The interviewer (TMP) made field notes during the interview and then summarized these following the interview. The field notes were thereafter used to develop analytical memos. Each interview

lasted approximately 40 minutes. The open-ended questions provided the participants with an opportunity to interact freely and to discuss their perspectives regarding support needs during pregnancy in detail. Written informed consent was sought and obtained from all the participants before the commencement of interviews. Permission to record the interviews was sought and obtained from participants prior to the interviews. We used a digital audio voice recorder and the recordings were safely stored in a password protected computer. Data collection was carried out in accordance with relevant guidelines and the participants could withdraw from the study at any point. Although the study was of a sensitive nature, we did not anticipate any negative effects on the participants. However, we had arranged counselling services for the participants if the need arises but none of the participants requested for these services.

Data Analysis

The audio-recorded data was transcribed verbatim by the first author (TMP). The anonymity of the participants was maintained by assigning a pseudonym to each. We chose thematic analysis as the method of analysis for the data because it allowed us the opportunity to conduct iterations through careful reading and re-reading of the data in order to discover underlying meanings and patterns and to produce a detailed account of the phenomenon [31, 32]. As qualitative descriptive research is purely data-derived [33], we specifically employed an inductive approach to thematic analysis (34).

The first author (TMP) performed the initial round of coding following the six steps described by Braun and Clark [34]. The first step in the process was familiarization with the data by reading and re-reading the transcripts to make summaries. Secondly, she (TMP) generated initial codes and this step was followed by identifying the emerging themes, which

is the third step. Fourthly, TMP reviewed potential themes, particularly checking for inconsistencies and whether the themes overlapped. Next, the themes were defined and named. Lastly, a report was produced using quotations of what the participants said to illustrate the themes [34].

It is important to note that this process was not straightforward as presented rather it was iterative. The initial coding was then reviewed and modified by the third author (OA) after which the second author (PN; a post-doctoral fellow and researcher), conducted a general review of the themes. All the authors revised, finalized and agreed upon the themes.

Findings

In total, twenty-four students who met our criteria participated in the study (Table 1). Among these, twenty-one were pregnant and three were in the puerperal period. All participants, including those who were in the puerperal period had disclosed their pregnancy to significant others.

Table 1 Socio-demographic details of participants (n = 24)

Participant pseudonyms	Age	Gestational stage	Number of previous pregnancies
Mbali	20	Seven months pregnant	None
Lindiwe	23	Eight months pregnant	None
Busi	19	Four months pregnant	None
Samantha	19	Three months pregnant	None
Nomthandazo	19	Four weeks puerperal	None
Bongiwe	21	Three months pregnant	None
Bella	26	Six months pregnant	One
Kholeka	21	Eight months pregnant	None

Zevile	23	Five months pregnant	None
Rachael	22	Five months pregnant	None
Thuli	21	Eight months pregnant	None
Njabulo	24	Six months pregnant	None
Khethiwe	24	Four months pregnant	One
Minenhle	20	3 weeks puerperium	None
Barbara	21	seven m[illegible]ths pregnant	None
Khanyisile	22	nine months pregnant	One
Thandeka	20	four months pregnant	None
Lerato	21	Five weeks puerperium	None
Zinhle	23	Six months pregnant	None
Thuli	20	Three months pregnant	None
Liyana	26	Seven months pregnant	One
Siyanda	19	Four months pregnant	None
Nandi	23	Seven months pregnant	None
Lindiwe	22	Three months pregnant	None

A summary of the characteristics of the participants are provided in Table 2. Four of the participants had one previous pregnancy while it was the first pregnancy for the remaining twenty participants. The findings are organized into four main themes and ten sub-themes identified from the analysis of the data. The four main themes are: Support needs of unmarried pregnant students; period when the most support is needed; male partner support required; and health systems role on social support.

Table 2 Summary of the socio-demographic details of participants

**Characteristics**		**Number of participants (Percentage)**
**Age of participants**	15-19	4 (16.7%)
	20-24	18 (75%)
	25-30	2 (8.3)
**Year of study**	1st year	7 (29%)
	2nd year	9 (37.5%)
	3rd year	5 (21%)
	Honours	3 (12.5%)
**Gestational age**	1st month-birth	21 (87.5%)
	Birth-6 weeks puerperium	3 (12.5%)
**Number of previous pregnancies**	None	20 (83%)
	1	4 (17%)

Support needs of unmarried pregnant students

Support during pregnancy was perceived to be very important among all the participants. All the participants said they could not do without social support. The support needs of the participants were similar in many ways but sometimes unique to their situation. The following three sub-th[illegible]es emerged from the interviews.

Emotional support

Some of the participants indicated that they felt a sense of shame because of their pregnancy. Other participants mentioned experiencing feelings of shame and embarrassment at university and in their communities as a result of their pregnancy. This was exacerbated when people would stare at them and that was considered to be a negative attitude.

> Some of my friends ignore me due to the pregnancy because they don’t wanna walk with me to school and I also see myself as totally different from them. At first, I felt so neglected now I have accepted that am pregnant and things won’t be the same. I

> usually walk alone. Some friends will support me, some will not. [Lind[illegible], honours student]

Emotional support was considered to be the most important type of support among participants. Some participants expressed their need for a ‘listening ear’ and the desire to be understood and not be judged for getting pregnant. Their accounts showed that they experienced feelings of sadness and loneliness and considered themselves vulnerable because they got pregnant whilst pursuing their studies. Personal vulnerabilities and situational constraints led to loneliness among the participants. Due to loneliness, the unmarried students in this study expressed a need for social relationships that would offer constant assurance that someone was there for them. Therefore, to minimize these feelings of loneliness, emotional support was considered to be the most important type of support that they needed to receive among all others.

> It feels good to have people around and knowing that you are loved. Although you made mistakes - not that a baby is a mistake – but being pregnant while you are studying, being something that you have not planned obviously. So, knowing that although you have made that mistake, but people haven’t abandoned you. People around you like your loved ones and your family- that they still love you [Slindile, 20 years old, 2 weeks pueperal].

In comparison to financial support, receiving emotional support and having people who were going to be there for them in times of sadness was said to be important and it made their pregnancy easier to handle.

> I just don’t need money in that kind of way. The kind of support that I would really like, that I would really expect is for people to be there for me, to give me that much attention, to be there when I feel sad [Rachael, six months pregnant]

For some participants, having someone available to offer emotional support was important regardless of who was offering it. They reported that the availability of emotional support made a huge difference in their lives regardless of its source because their greatest need was to feel loved and cared for. Having people to talk to and understand them, without judging them was important for participants.

> A person who is pregnant just needs support, love - no matter how bad the situation is or how it has happened. Having people to talk to and that understand, are really there for you, lending their ear more than their mouth, people to talk to [Samantha, seven months pregnant].

Instrumental support

Receiving help with tasks and help to manage their time was an important support need of some participants. Their narratives revealed that instrumental support was most needed at the end of the pregnancy because as the pregnancy progressed, it became more difficult to be mobile. Therefore, they needed people to be around and help them with chores, academic tasks, accompanying them to certain places especially hospitals or going to buy them food and groceries. One participant noted that she needed company every time she was going to the health facilities unlike before she got pregnant.

> If I was sick and I wasn't pregnant I'd be like, "no please, don't come, I'll go by myself". Now! [that she is pregnant], Now I can't go to the clinic by myself, I'll just call anyone, call my sister or my aunt, she's always there she's like no I'll take you. If it [support] had to stop now, I don't know what I would do [Amahle, 18 years old, seven months pregnant].

Another participant revealed how overwhelming the tasks had become in her last trimester of pregnancy and the importance of receiving instrumental support during this period.

> When you are nearing your end [approaching delivery], you have so much to do. You have got school things to do, you have got doctors' appointments consistently one after the other, you have to buy this, get this, get that, that's when you need a more rigid [solid] support system [Khanyisile, Honours student, nine months pregnant]

These excerpts show that apart from the need to feel better about their situation, unmarried pregnant students need help with tasks to relieve them of physical stress. In addition, receiving help with tasks also had an effect on their emotional wellbeing.

Informational support

The narratives of the participants in this study revealed that young women, particularly first-time mothers are usually inexperienced with pregnancy and childbirth and need advice and information. Some participants reported that they needed people around them that could help them with information about pregnancy, childbirth and how to care for their baby.

> I do not have experience on how to bath the baby, feed the baby well when it's crying, what's wrong. They [people] should tell me what to expect if am not figuring out, they must assist me [Nomthandazo, nine months pregnant].

Other participants also indicated that they needed information on what to expect during the different stages of pregnancy and childbirth. One of the participants who had a previous pregnancy explained the difficulties of having a first pregnancy without adequate knowledge of the accompanying physical changes.

> At the beginning of pregnancy, maybe you are not sure what is happening to you, may be for some people it's the first time you don't know what is happening, all the sickness. At the end that's exactly what's happening to you, breaking water and all

> that. Some of the things you wouldn't know so someone should tell you [Naledi, nine months pregnant, second pregnancy].

Participants require guidance on pregnancy with respect to proper nutritional practices, adequate prenatal care and preparation for labour and delivery. Due to lack of information on proper nutrition practices, some participants risk having low birth weight infants as illustrated below:

> After I fell pregnant, I did not eat. I don't usually eat. I didn't know that when you fall pregnant you have to change your lifestyle and do things in a certain way. It was a shock when I went for a scan when I was five months pregnant and they said my baby looks like he's two or three months, he was underdeveloped. So, the doctor said "it's either your child is gonna be disabled or when you give birth, you're gonna have to stay in hospital for a long time cause he's really small" [Samantha, 19 years old, six months pregnant].

The participant's narrative highlights the challenges of poor nutritional intake during pregnancy.

Period when the most support is required

When asked about the specific period during pregnancy when they feel the most support is required, participants had different views and four sub-themes arose.

At the beginning of pregnancy

Some of the participants said they needed the most support, particularly emotional support at the beginning of the pregnancy. This was a period when they had just found out about their pregnancy which was something new to them. At the same time, they were still dealing with acceptance of the pregnancy whether personally or in their relationship. Therefore, they

indicated that at this initial stage of pregnancy, they required some form of assurance from someone that they were going to be supported.

> I think I needed more support at the beginning. It was something new to me. I kind of needed someone to hold my hand and say 'it's gonna be fine, I'm gonna be therefore you, don't worry, we'll get through this together' [Thandeka, 19 years old, four months pregnant].

In addition, this was the period when they were likely to be hiding the pregnancy from their family when in actual fact, they needed their support. Therefore, they found it difficult to share their stressors with others.

> It was difficult before I told my parents because you know when nobody knows at home and you start experiencing these things [disagreements/fights] with your boyfriend and you get pissed, like you wanna tell someone but you can't. [Amahle, 18 years old, seven months pregnant].

Therefore, it is important to receive support at the beginning of pregnancy for better maternal and new-born health outcomes. The different experiences that the participants had in their pregnancies determined what they perceived their support needs to be and the period that they were required.

Throughout pregnancy

Other participants said they required support throughout their pregnancy and into the puerperium because they felt their support needs were not going to change over the course of the pregnancy. Therefore, they said that they needed to receive support through every period of the pregnancy.

> Throughout the pregnancy you need support because there are times where you feel like I wish I could just give up everything like you know, school cause sometimes you find it frustrating, and you don't wanna deal with it. I think you need that pillar of strength every day; someone that will be calling you and asking you 'How are you doing? How's everything? How's the baby?' Like I think every day during pregnancy even after pregnancy [Zinhle, 25 years old, six months pregnant].

Towards the end of pregnancy

Some participants felt they needed more support as they approached their delivery date. The support required at this stage was mostly instrumental support. They indicated that it was difficult to balance antenatal care (ANC) schedules at the public clinics and academic obligations in the final trimester. This suggests that in the last trimester of pregnancy, students experience a task overload. For this reason, they need instrumental support in the final trimester of pregnancy.

> It was at the end [when she needed more support]. When you feel like the baby is about to come, you have to prepare for everything. With schoolwork it's very hard. Because at the end, I had to attend clinics more than before, like almost every week. So, the time you are attending clinics and everything, you are using up the time for studying [Slindile, 20 years old, two weeks puerperal].

One participant expressed her need to always have someone with her so that she would be well prepared in the event of labour.

> Mostly it's at the end when you'll be delivering (when she needs the most support) because at that time you have a lot of complications, you need someone to look after you because you can deliver anytime. You need to have transport that will take you to

> the hospital immediately and always bring your card so that you are well prepared [Thuli, 19 years old, eight months pregnant,].

Support post delivery

Young women usually require support and guidance after childbirth and in the case of students; they need support with childcare as they continue with their studies. Except for two who were not sure, other participants expressed their intention to continue with their studies after their child is born. When asked about childcare, some participants' responses were that their mothers or female guardians were going to look after the baby upon delivery. In cases where mothers were unavailable due to distance or employment, they were still going to offer support by paying a helper to look after the child. Therefore, parents were also going to provide financial support to a large extent. Young women in this study relied heavily on their own mothers and depended on them for childcare.

> I'll still be in Durban when I give birth cause am giving birth in September. So, my mom will travel here. My mom said "give me the child then you go back to school" and she's gonna take care of the baby...In terms of finances my mom will support me. I don't know about my child's father whether he'll be there or not [Thandeka, second year student].

Those who did not have mothers (due to death) or those whose mothers worked faraway would receive help from other older female members of the family such as aunties and sisters.

> My mother`s sister, she lives in Maritzburg [Pietermaritzburg], she did say she can take care of baby once the baby is born so that I can go back to school and study cause that is what I want to do. She was the one who volunteered to do that [Nomthandazo, first year student]

When participants were asked about financial support, some of their accounts revealed that providing for the child's welfare was the father's responsibility.

> The baby needs so many things so as to grow in a good state; nappies, warm clothes, milk, formula [baby food] since am not going to breastfeed so financially it will be much required from him. [She gives an example]-cause my cousin gave birth in April and the father of the baby hasn't given her a single cent since she gave birth, since she fell pregnant, she hasn't received any money from the baby's daddy and telling me things are difficult, so financially it is so important [Thobile].

Male partner support needed

One universal theme that emerged in all the interviews with respect to support needs was receiving support from the male partner. Male partner support was considered to be the most important source of support that unmarried pregnant students required. The lack of emotional support from male partners during pregnancy was seen to have grave consequences for both the mother and her unborn baby. These include depression, resentment and abortion.

> As much as you are the one carrying the child, you can't go through everything by yourself. I think if you don't have your pa[illegible]ers support, you can end up having resentment towards him, including the child - somebody who has no contribution towards the baby is painful [Khanyisile, Honours student].

Partner's emotional support

Receiving emotional support from partners was the biggest support need among young women. When compared to other support needs, some participants reported that they would rather have their partner to be there for them emotionally than financially. The emotional

support required by these participants ranged from the simplest acts of care like being there to talk to them or calling them every day.

> I would like to be supported emotionally because money doesn’t buy love. It is the tender loving care that is most important. I appreciate that because that is what is important, that is what I prefer, it’s what I need [Naledi, nine months pregnant]

Other participants said that their partners needed to be their greatest source of support because they were the reason why they got pregnant. Therefore, they needed to take responsibility for ‘what they did’ and ‘compensate for their mistake’. This means that male partners were expected to play a major role in providing support and relieving emotional distress.

> You know when you are pregnant, I think you expect a lot when you know that this person is the baby’s father, it’s like you want them to do more. You want them to try anyway and compensate for doing what they did to you, because if it wasn’t for him I actually wouldn’t be pregnant so he has to be there for me [Zinhle, second year student, six months pregnant]

There was a general view among some participants that the relationship with their partner should remain as it was before pregnancy or improve with the prospect of a child. However, it was found that in a lot of cases, relationships became weak as the pregnancy progressed. In some cases, the pregnancy led to the end of the relationship. Others revealed that relationships become more about the wellbeing of the unborn baby and less about the romantic relationship between the partners. Some participants reported feeling neglected and expressed longing for a romantic relationship as they did before the pregnancy.

> I don`t want him to treat me as the mother of his babies, I want him to treat me also as his girlfriend. Now we usually talk about the baby a lot. We don’t talk about us. I

> want us to date like we used to before, go out with him, watch some movies and get a lot of support from him [Thuli, second pregnancy].

Partner's instrumental support

Having a partner who is available to provide material goods and help with tasks was important among the participants. Instrumental support such as having the partner's company when they go places and buying them foods that they crave for was considered to be an important support need. The smallest acts of help with tasks were considered to be of great importance even in cases where partners could not offer financial support.

> Sometimes people ask questions about him [her partner] when you are walking alone. I need the support. If he was not there for me, it would have hurt me you know, to know that people are laughing at me [Thobile, eight months pregnant].

One participant complained about her partner who is not usually willing to help her with tasks.

> Sometimes he'll be sleeping [when he visits her in her campus room] and I have to do my schoolwork then when I ask him to do something like can you please get me some water, sometimes he's gonna be like he's tired or when I want him to do something for me he's gonna complain [Zinhle, six months pregnant].

Instrumental support was considered to be crucial particularly in matters involving doctors' appointments and preparations for the arrival of the baby. In cases of medical emergencies, partners were required to be present. Going to the hospital together was considered to be an important aspect of instrumental support as it assured them that they were not going through their pregnancy alone.

> Before I fell pregnant, I really didn't notice anything, and it didn't matter. Now it's more like I want more, more time with him. Sometimes when you go to the clinic and you see people with their boyfriends and then I'll be thinking like, where's mine? I also want him to be here and experience this together. [Amahle, seven months pregnant].

Partner's financial support

All the participants said that their tuition is paid by their parents. Therefore, the financial resources expected from male partners were for issues concerning the pregnancy. Financial needs from partners included money to buy food that they crave, maternity clothes, hospital bills and items for the baby. However, more financial support was expected after delivery as compared to during pregnancy.

> I need him not to run away from his responsibilities when the baby is here, that's all I'm just hoping for, that he'll be a father and support his child [Lindiwe, 22 years old, eight months pregnant].

Financial support did not always mean receiving money from their partners. Having their partners buy or pay for their needs was also regarded as financial support.

> Well, my partner is not good at giving money and all that, but he can do everything and anything to support me, he put me on his medical aid [medical insurance], he can buy me anything that I crave for, but giving money no. So, he supports me, yes [Naledi, second year student].

The payment of damages (Inhlawulo) was considered to be a form of financial support as it was money that could be used by the family to support the pregnant women. This study found that some of the participant's partners had not paid this fine to the young woman's family. In

the current study, men who paid damages to their partner’s family were perceived to be more responsible and could be entrusted with caring for them. The payment of damages was observed to have a calming effect on angry parents and associated with a more positive attitude towards the pregnancy. Therefore, it was considered as a type of support that was required of partners to assure the family that he would honour his responsibility.

> My sister is the one who advised me about (partner) paying the damage in December because that is when my dad is coming from Joburg. I want him (father) to know that even if he chases me [from his house?], he will know that everything was done perfect and I’m not staying with, what can I say, a thug or anything. I want him to know that am staying with the real man [Mbali, 20 years old].

On the other hand, men who did not pay damages were seen as untrustworthy.

> To be honest sometimes I think he is going to run away, since he hasn`t paid damages and he can see the other girls, and forget about me you know, you know how boys are [Nomthandazo, nine months pregnant].

Health systems role on social support and influence on male partner support

Health professionals are able to contribute to the psychological wellbeing of women during pregnancy and during the postpartum period by offering support. When asked about their experiences at ANC clinics, some participants reported that the health staff at the clinics had a negative attitude towards them. They felt stigmatized because of their age and status as students, and this was because of what the nurses would say about them.

> Ooh at first, I had hell, like the nurses ask you basic questions: “how old are you?” Then when you say 18 then everybody is like “hau?” then instantly when you say you

> go to Howard College everybody assumes like “no wonder, it’s expected”. [continues to mimic] “Where’s the baby’s daddy?” Am like “he’s working”. They’re like “yeah, you have a working baby’s daddy because that’s what you do”. They weren’t really saying it to my face, but you can hear them gossiping [Amahle, 18 years old]

When asked about how the health systems influence male partner support among pregnant students, particularly partner ANC attendance, almost all the participants said they had never attended an ANC session with their partner. Some participants said their partners did not want to go to the clinics with them because of the female dominated environment at the clinic and the amount of time spent at the clinic. In addition, nurses did not seem to be concerned about the absence of partners.

> I wanted him to be there, but he suggested that no, he will remain behind, or he will go with me and leave me there. Personally, I understand why he doesn’t want to stay with me. Clinics are just something else, many people and you wait for hours. The nurses don’t even ask about my partner. [Thobile, 19 years old]

Some participants explained that they were not attending ANC in public clinics but instead were consulting specialists like gynaecologists and obstetricians in private hospitals to monitor their pregnancy. This was mostly because they felt that they needed to be checked by specialists who were not readily available in public clinics.

> With my partner first of all he said he doesn’t want me to go to clinics and all that, so he put me on his medical aid [medical insurance]. He didn’t want me to be going to the clinics. There’s nothing wrong with the clinic but for me he preferred the gynaecologist who is gonna do everything [Naledi, second pregnancy]

Some participants mentioned that their partner did accompany them to the clinic on first visit but did not do so on subsequent visits. While they had the desire to have their partners come

to the clinics with them, they mentioned that they did not see the purpose because nurses at the clinic did not involve men in any of the activities.

> He came with me to the clinic on my second appointment, when I was about four months. It means a lot to have someone with you. But they didn't ask him any questions or asked him to do any tests [Slindile, new mother].

Overall, it was established that most unmarried pregnant students seek ANC in public clinics and hospitals alone without the support of their male partners. The participants generally considered ANC to be unfriendly. In addition, partner attendance was not specifically encouraged or recommended by healthcare workers.

Discussion

Unmarried pregnant students need emotional, instrumental, informational and appraisal support. Research on social support has consistently found that the absence of social support is a significant risk factor for antenatal and postnatal depression and anxiety among pregnant women [35, 36]. This study sought to explore the support needs of young women during pregnancy and the puerperium. Our findings suggest that the socio-cultural context of pregnancy among young women results in social stigma. Unmarried pregnant students are stigmatized in the academic environment as well as in the community. This study found that these students are sometimes ignored by their peers because of the pregnancy. This leads to social isolation, a common effect of pregnancy among young people [37]. Social isolation has been associated with loneliness, emotional distress and depressive symptoms. Experiencing depressive symptoms in pregnancy has consequences for maternal and child health outcomes because it is associated with low birth weight [38].

Four different types of social support (emotional, instrumental, informational, and financial) were highlighted as an important resource to cope with stressors by all participants. Although financial support is usually categorised as instrumental support, in this study we categorised it as an independent form of support as it was a salient theme in the study. Emotional support was described as having people who are available to share their feelings with, encourage them and show acts of caring towards them. Instrumental support expressed by participants included receiving help with tasks such as going for medical check-ups. The participants in our study indicated that they require informational support, which is receiving information on pregnancy, childbirth and how to take care of a new-born baby. Lastly, financial support was needed to buy food, to cover transport costs and medical expenses and to prepare for the arrival of the unborn baby. All these types of support play a role in fostering positive maternal and child health outcomes.

An important finding of our study is that, among the different types of social support needed, emotional support was deemed more important by the participants. Pregnancy was associated with loneliness and fear of being judged for falling pregnant within an academic setting. The young women desired to be listened to and loved more especially because of their condition which was associated with various stressors. Although a previous study reported on the importance of emotional support during pregnancy, it was not regarded as the most important type of support needed [39]. The provision of re-assurance, feeling valued and sympathetic listening result in feelings of comfort and is associated with decreased levels of depressed mood and anxiety during pregnancy and postnatal [40]. In line with Cohen and Will’s [41] stress buffering hypothesis of social support, unmarried pregnant students perceive emotional support to buffer the effects of psychological distress during pregnancy. Previous studies have also shown that perceived social support may have a positive effect on pregnant women

causing them to perceive their pregnancy as less stressful and providing a resource for effectively dealing with stressful situations such as labor [42, 43]

For unmarried pregnant students, who usually stay at university residences, instrumental support was perceived as important in assisting to balance the academic demands and pregnancy-related demands. The pregnancy came with additional responsibilities such as regular visits to the clinic and performing previously manageable tasks was perceived as daunting as a result of the pregnancy. A study in Ghana among women with new-borns and various community stakeholders found that women were dependent on network members for instrumental support to get them to a health facility for childbirth [44]. However, instrumental support seemed unavailable to pregnant women residing at university residences as they did not stay with their families. The narratives of the participants revealed that young women, particularly first-time mothers were usually inexperienced with pregnancy and childbirth and need advice and information. In a previous study, 70% of first-time pregnant women indicated that they needed information about pregnancy and childbirth [45]. The participants expressed a lack of knowledge regarding pregnancy-related sickness, delivery process, and dietary needs as well as taking care of the newly born child. A recent study among adolescent pregnant women has shown knowledge deficit on the benefits of ANC and nutrition during pregnancy, that may contribute to poor pregnancy outcomes [46].

Our study sought to understand the stage at which social support was deemed more important by the unmarried pregnant students. Social support was identified as important at different phases of pregnancy. In the first few months of pregnancy, the expectant mother develops new and often uncomfortable physical and emotional symptoms such as nausea, feelings of uncertainty and increased emotional expressions [47, 48]. In the early stages of the pregnancy, young women desired support from significant others as they were still in the process of embracing the pregnancy and thus required acceptance and assurance that they

would be supported throughout the whole pregnancy experience. Towards the end of the pregnancy, informational support was deemed more important to help balance between academic demands and regular visits to the clinic. More so, the narratives from most of the participants indicate that support was necessary throughout the whole period of pregnancy and after giving birth. The various stressors faced by unmarried pregnant students came at different stages of the pregnancy and were accentuated by the challenging academic environments characterised by high workload, meeting deadlines and socio-economic issues [49, 50]. Thus, an enduring and reliable support network was essential.

While support from different sources is important for unmarried pregnant students, participants identified male partner support as their most important source of support. Unlike community-dwelling women in Bangladesh who cited mothers and sisters-in-law as top sources of emotional support [39], participants desired their partners to be emotionally available and care for them since they were partly involved in conceiving the child. Male partners are expected to take responsibility and be available to offer emotional support to young women throughout pregnancy and after childbirth. The availability of support from male partners gives women a more positive attitude towards their pregnancy and assures them that they are not alone thereby reducing emotional distress [19]. Partner support is an important protective factor throughout conception and pregnancy and our study corroborate these findings. Importantly, participants did not want to be perceived by their partners as mothers only, but as lovers, as that entailed emotional connection. This type of support and relationship status was deemed more important than financial support. A study among couples indicated that more prenatal shared leisure predicted higher marital love and less conflict when the baby was 1 year old [51]. Even though participants in the current study were unmarried it seems that they would benefit from spending more quality time with their partners. A previous study showed that male partners who did not approve an unplanned

pregnancy were less likely to be emotionally available increasing the risk of psychological distress in women [3]. In instances where emotional support from male partners is not feasible, there is a need to design interventions that will assist unmarried pregnant students in identifying alternate sources of support such as counselling support, seeking social support from friends and their social circles as well as through the social media.

Partners' instrumental and financial supports were also considered to be important sources of support. The participants wanted to be accompanied to clinic visits and experience the pregnancy process with their partners. In previous studies, male involvement in maternal and new-born health has been associated with a variety of improved health outcomes including increased maternal access to antenatal and postnatal services, improved maternal mental health and reduced postpartum depression [52, 53]. In some African cultures, payment of damages is perceived as a form of acceptance of paternity [54]. This process was perceived by the participants to give a form of dignity to her family and some assurance that the father was prepared to take care of the child. However, similar to a previous study, expectations of partner support (emotional, instrumental and financial) are not usually met resulting in disappointment, conflicts with their partners and sometimes difficult break ups [8]. The current study found that experiencing an unintended pregnancy can lead to the disruption of a relationship. These findings complement the results of research showing that unplanned pregnancies are accompanied by family disruption and a decline in the marital quality and the couple's relationships [3, 55]. This results in emotional distress for unmarried pregnant students who may have to experience pregnancy without the support of their partner.

Unmarried pregnant students experienced social stigma at health facilities. This finding is consistent with the results from a previous study among healthcare providers in Ugu district in southern KwaZulu-Natal, South Africa which showed that health personnel exhibit a negative attitude towards pregnant young women [56]. The South Africa Department of

Health guidelines recommend that pregnant women seek ANC as soon as they suspect a pregnancy and encourages all pregnant women to attend at least eight ANC visits [57]. However, the experience of social stigma at public health facilities is likely to result in unmarried pregnant students shunning ANC, yet most of them cannot afford to use private healthcare services. Young women in our study also expressed the desire to have male partners attend ANC visits with them. However, it was reported that this does not usually happen due to the amount of time spent at ANC clinics, the female dominated environment and the negative attitudes from the nurses. These findings add to previous research which demonstrated that the main barrier to male participation in ANC was the healthcare facility environment [58]. Further, during ANC visits, nurses only ask about the existence of male partners but do not necessarily encourage partner attendance of ANC. Therefore, there is minimal influence from the healthcare professionals on emphasizing partner attendance of ANC, their perceived roles and benefit to the mother and the baby. Health systems have a role to play in fostering supportive relationships between pregnant women and their partners. Partner support and involvement in maternal healthcare has been shown to enhance maternal health outcomes [59].

Among women of all ages, pregnancy is associated with a myriad of stressors. In the contexts of academic institutions, pregnancy-related stressors are likely to add to the academic challenges faced by female students [60]. Absence or lack of emotional social support from significant others, particularly partners may exacerbate the stressors of pregnancy among students. Tertiary institutions should partner with established social initiatives offering services for men in the country to engage and mentor young men one-on-one and in groups about the importance of the father's role in the child's development and how to best support their partners to improve pregnancy outcomes, irrespective of relationship status. Culturally sensitive health promotion programmes targeted at 'father inclusive' practices and behaviour

change communication programmes to stimulate social support may have positive changes in terms of the supportive role of partners. To reach many young men, implementors of such programmes should consider adopting innovative outreach strategies that appeal to young men’s interests in their hang-out spots, such as bars and sports arenas.

Male involvement programmes can be integrated in ANC whereby services are restructured to accommodate male partners to ensure a sense of involvement and responsibility. However, for these programmes to work, the ANC environment should be user friendly for young women and their partners. A rights-based approach must be consistently adopted when delivering maternal healthcare [61]. Training and on-going education should be prioritised for healthcare workers regarding the provision of positive, respectful and supportive non-judgmental antenatal and postnatal care to their clients irrespective of their age or marital status. Pregnancy at universities is not uncommon and having clinics at university campuses equipped with comprehensive ANC may overcome some structural barriers such as the burden of transport costs and long waiting times at public clinics.

Conclusion

This study identified support needs of pregnant women in their transition to motherhood. Among the support needs of unmarried pregnant students, emotional support is perceived to be the most important type of support. Male partners appear to be the most sought-after source of emotional support for unmarried pregnant students as they desire to experience the emotional connection that existed before the pregnancy and fear the possibility of abandonment. Given the several challenges that they are faced with, unmarried pregnant students need all the available support to enhance wellbeing as they try to cope with academic and pregnancy-related stressors.

Acknowledgements

The authors would like to express their sincere thanks to the participants for the value they added to the study

References

1. Erfina E, Widyawati W, McKenna L, Reisenhofer S, Ismail D. Adolescent mothers' experiences of the transition to motherhood: An integrative review. Int J Nurs Sci. 2019;6(2):221-8. https://doi.org/10.1016/j.ijnss.2019.03.013
2. Schetter CD, Tanner L. Anxiety, depression and stress in pregnancy: implications for mothers, children, research, and practice. Curr Opin Psychiatry. 2012;25(2):141-148.
3. Barton K, Redshaw M, Quigley MA, Carson C. Unplanned pregnancy and subsequent psychological distress in partnered women: a cross-sectional study of the role of relationship quality and wider social support. BMC Pregnancy Childbirth. 2017;17(1):1-9. https://doi.org/10.1186/s12884-017-1223-x
4. Atuyambe L, Mirembe F, Annika J, Kirumira EK, Faxelid E. Seeking safety and empathy: adolescent health seeking behavior during pregnancy and early motherhood in central Uganda. J Adolesc. 2009;32(4):781-96. https://doi.org/10.1016/j.adolescence.2008.10.012
5. Colciago E, Merazzi B, Panzeri M, Fumagalli S, Nespoli A. Women's vulnerability within the childbearing continuum: A scoping review. Eur J Midwifery. 2020;12(4):18. https://doi.org/10.18332/ejm/120003
6. Mbelle N, Mabaso M, Setswe G, Sifunda S. Predictors of unplanned pregnancies among female students at South African Technical and Vocational Education and Training colleges: Findings from the 2014 Higher Education and Training HIV and AIDS survey. S Afr Med J. 2018;108(6):511-6. https://dx.doi.org/10.7196/samj.2018.v108i6.12744

7. El-Adas A. The resolution of unintended pregnancy among female students at the University of Ghana, Legon. 2007, Regional Institute for Population Studies: University of Ghana, Legon
8. Govender D, Naidoo S, Taylor M. “I have to provide for another life emotionally, physically and financially”: understanding pregnancy, motherhood and the future aspirations of adolescent mothers in KwaZulu-Natal South, Africa. BMC Pregnancy Childbirth. 2020;20(1):1-21. https://doi.org/10.1186/s12884-020-03319-7
9. Nkosi KB, Makhene A, Matlala S. Educational challenges as experienced by pregnant student nurses at a college in Mpumalanga. Curationis. 2019;42(1):1-7. https://doi.org/10.4102/curationis.v42i1.1880
10. Kebede A, Molla B, Gerensea H. Assessment of risky sexual behavior and practice among Aksum University students, Shire Campus, Shire Town, Tigray, Ethiopia, 2017. BMC Res Notes. 2018;11(1):1-6. https://doi.org/10.1186/s13104-018-3199-7
11. Pascoe MC, Hetrick SE, Parker AG. The impact of stress on students in secondary school and higher education. Int J Adolesc Youth. 2020;25(1):104-12. https://doi.org/10.1080/02673843.2019.1596823
12. Mudhovozi P. Social and academic adjustment of first-year university students. J Soc Sci. 2012;33(2):251-9. https://doi.org/10.1080/09718923.2012.11893103
13. Moseson H, Mahanaimy M, Dehlendorf C, Gerdts C. “… Society is, at the end of the day, still going to stigmatize you no matter which way”: A qualitative study of the impact of stigma on social support during unintended pregnancy in early adulthood. PLoS One. 2019;14(5):e0217308. https://doi.org/10.1371/journal.pone.0217308
14. Wang H, Long L, Cai H, Wu Y, Xu J, Shu C, et al. Contraception and unintended pregnancy among unmarried female university students: a cross-sectional study from

China. PLoS One. 2015;10(6):e0130212. https://doi.org/10.1371/journal.pone.0130212

15. Wahn EH, Nissen E. Sociodemographic background, lifestyle and psychosocial conditions of Swedish teenage mothers and their perception of health and social support during pregnancy and childbirth. Scand J Public Health. 2008;36(4):415-23.
16. Birkeland R, Thompson JK, Phares V. Adolescent motherhood and postpartum depression. J Clin Child Psychol. 2005;34(2):292-300. https://doi.org/10.1207/s15374424jccp3402_8
17. Figueiredo B, Bifulco A, Pacheco A, Costa R, Magarinho R. Teenage pregnancy, attachment style, and depression: A comparison of teenage and adult pregnant women in a Portuguese series. Attach Hum Dev. 2006;8(2):123-38. https://doi.org/10.1080/14616730600785686
18. Hildingsson I, Tingvall M, Rubertsson C. Partner support in the childbearing period—A follow up study. Women Birth. 2008;21(4):141-8. https://doi.org/10.1016/j.wombi.2008.07.003
19. Stapleton LR, Schetter CD, Westling E, Rini C, Glynn LM, Hobel CJ, et al. Perceived partner support in pregnancy predicts lower maternal and infant distress. J Fam Psychol. 2012;26(3):453-463. https://doi.org/10.1037/a0028332
20. House J. Work, stress and social support. Menlo Park, CA: Addison-Wesley; 1981.
21. Raman S, Srinivasan K, Kurpad A, Dwarkanath P, Ritchie J, Worth H. 'My Mother… My Sisters… and My Friends': Sources of maternal support in the perinatal period in urban India. Midwifery. 2014;30(1):130-7. https://doi.org/10.1016/j.midw.2013.03.003

22. Cofie LE, Barrington C, Sodzi-Tettey S, Ennett S, Maman S, Singh K. A qualitative study of women’s network social support and facility delivery in rural Ghana. PLoS One. 2018;13(11):e0206429. https://doi.org/10.1371/journal.pone.0206429
23. McDonald AD, McDonald JC, Armstrong B, Cherry NM, Nolin AD, Robert D. Prematurity and work in pregnancy. Occup Environ Med. 1988;45(1):56-62.
24. McLeish J, Redshaw M. Mothers’ accounts of the impact on emotional wellbeing of organised peer support in pregnancy and early parenthood: a qualitative study. BMC Pregnancy Childbirth. 2017;17(1):1-4. https://doi.org/10.1186/s12884-017-1220-0
25. Iranzad I, Bani S, Hasanpour S, Mohammadalizadeh S, Mirghafourvand M. Perceived social support and stress among pregnant women at health centers of Iran-Tabriz. J Caring Sci. 2014;3(4):287-295. https://doi.org/10.5681/jcs.2014.031
26. Ginja S, Coad J, Bailey E, Kendall S, Goodenough T, Nightingale S, et al. Associations between social support, mental wellbeing, self-efficacy and technology use in first-time antenatal women: data from the BaBBLeS cohort study. BMC Pregnancy Childbirth. 2018;18(1):1-1. https://doi.org/10.1186/s12884-018-2049-x
27. Ulin PR, Robinson ET, Tolley EE. Qualitative Methods in Public Health: A Field Guide for Applied Research. San Francisco, CA: Jossey-Bass; 2005.
28. Sandelowski M. Whatever happened to qualitative description?. Res Nurs Health. 2000;23(4):334-40.
29. Polkinghorne DE. Language and meaning: Data collection in qualitative research. J Couns Psychol. 2005;52(2):137. https://doi.org/10.1037/0022-0167.52.2.137
30. Padfield M, Procter I. The effect of interviewer's gender on the interviewing process: A comparative enquiry. Sociology. 1996;30(2):355-66.
31. Babbie E. Research design. The practice of social research. 12th ed. Belmont, CA: Cengage Learning; 2010.

32. Rice PL, Ezzy D. Qualitative research methods: A health focus. Melbourne: Oxford University Press; 1999.

33. Lambert VA, Lambert CE. Qualitative descriptive research: An acceptable design. Pac Rim Int J Nurs Res. 2012;16(4):255-6.

34. Braun V, Clarke V. Using thematic analysis in psychology. Qual Res Psychol. 2006;3(2):77-101.

35. Shafaie FS, Mirghafourvand M, Rahmati M, Nouri P, Bagherinia M. Association between psychological status with perceived social support in pregnant women referring to Tabriz health centers. J Matern Fetal Neonatal Med. 2018;31(12):1554-60. https://doi.org/10.1080/14767058.2017.1319934

36. Rashid A, Mohd R. Poor social support as a risk factor for antenatal depressive symptoms among women attending public antennal clinics in Penang, Malaysia. Reprod. Health. 2017;14(1):1-8. https://doi.org/10.1186/s12978-017-0404-4

37. Ellis-Sloan K, Tamplin A. Teenage mothers and social isolation: The role of friendship as protection against relational exclusion. Soc Policy Soc. 2019 Apr;18(2):203-18. https://doi:10.1017/S1474746418000106

38. Li X, Gao R, Dai X, Liu H, Zhang J, Liu X, Si D, Deng T, Xia W. The association between symptoms of depression during pregnancy and low birth weight: a prospective study. BMC pregnancy and childbirth. 2020;20(1):1-7. https://doi.org/10.1186/s12884-020-2842-1

39. Edmonds JK, Paul M, Sibley LM. Type, content, and source of social support perceived by women during pregnancy: Evidence from Matlab, Bangladesh. J Health Popul Nutr. 2011;29(2):163-173. https://doi.org/10.3329/jhpn.v29i2.7859

40. McLeish J, Redshaw M. Mothers' accounts of the impact on emotional wellbeing of organised peer support in pregnancy and early parenthood: a qualitative study. BMC Pregnancy Childbirth. 2017;17(1):1-4. https://doi.org/10.1186/s12884-017-1220-0

41. Cohen S, Wills TA. Stress, social support, and the buffering hypothesis. Psychol Bull. 1985;98(2):310.

42. Giesbrecht GF, Poole JC, Letourneau N, Campbell T, Kaplan BJ, APrON Study Team. The buffering effect of social support on hypothalamic-pituitary-adrenal axis function during pregnancy. Psychosom Med. 2013;75(9):856-62. https://doi:10.1097/PSY.0000000000000004

43. Basharpoor S, Heydarirad H, Daryadel SJ, Heydari F, Ghamari Givi H, Kishore J. The role of perceived stress and social support among predicting anxiety in pregnant women. J Holist Nurs Midwifery. 2017;27(2):9-16. https://doi:10.18869/acadpub.hnmj.27.2.9

44. Moyer CA, Adongo PB, Aborigo RA, Hodgson A, Engmann CM. 'They treat you like you are not a human being': maltreatment during labour and delivery in rural northern Ghana. Midwifery. 2014;30(2):262-8. https://doi.org/10.1016/j.midw.2013.05.006

45. Singh D, Newburn M, Smith N, Wiggins M. The information needs of first-time pregnant mothers. Br. J. Midwifery. 2002;10(1):54-8. https://doi.org/10.12968/bjom.2002.10.1.10054

46. Kululanga LI, Kadango A, Lungu G, Jere D, Ngwale M, Kumbani LC. Knowledge deficit on health promotion activities during pregnancy: the case for adolescent pregnant women at Chiladzulu District, Malawi. BMC Pregnancy Childbirth. 2020;20(1):1-9. https://doi.org/10.1186/s12884-020-03386-w

47. Bjelica A, Cetkovic N, Trninic-Pjevic A, Mladenovic-Segedi L. The phenomenon of pregnancy-A psychological view. Ginekol Polska. 2018;89(2):102-6. https://doi.10.5603/GP.a2018.0017

48. Bonillas CA, Feehan R. Normalizing the changes experienced during each trimester of pregnancy. J Perinat Educ. 2008;17(1):39-43. https://doi.org/10.1624/105812408X266287

49. Reddy KJ, Menon KR, Thattil A. Academic stress and its sources among university students. Biomed Pharmacol J. 2018;11(1):531-7. https://dx.doi.org/10.13005/bpj/1404

50. Kumaraswamy N. Academic stress, anxiety and depression among college students: A brief review. International review of social sciences and humanities. 2013;5(1):135-43.

51. Claxton A, Perry- Jenkins M. No fun anymore: Leisure and marital quality across the transition to parenthood. J Marriage Fam. 2008;70(1):28-43.

52. Tokhi M, Comrie-Thomson L, Davis J, Portela A, Chersich M, Luchters S. Involving men to improve maternal and newborn health: a systematic review of the effectiveness of interventions. PLoS One. 2018;13(1):e0191620. https://doi.org/10.1371/journal.pone.0191620

53. Yargawa J, Leonardi-Bee J. Male involvement and maternal health outcomes: systematic review and meta-analysis. J Epidemiol Community Health. 2015;69(6):604-12. http://dx.doi.org/10.1136/jech2014-204784

54. Nathane-Taulela M, Nduna M. Young women’s experiences following discovering a biological father in Mpumalanga province, South Africa. Open Fam Stud J. 2014;6(1):62-68.

55. Kazemi A, Ghaedrahmati M, Kheirabadi G. Partner's emotional reaction to pregnancy mediates the relationship between pregnancy planning and prenatal mental health. BMC Pregnancy Childbirth. 2021;21(1):1-8. https://doi.org/10.1186/s12884-021-03644-5
56. Govender D, Taylor M, Naidoo S. Adolescent pregnancy and parenting: Perceptions of healthcare providers. J Multidiscip Healthc. 2020;13:1607-1628. https://doi.org/10.2147/JMDH.S258576
57. National Department of Health, South Africa. Guidelines for Maternity Care in South Africa. A Manual for Clinics, Community Health Centres and District Hospitals. 4th ed. Pretoria: NDoH, 2015:172. http://www.health.gov.za/index.php/2014-03-17-09-9-38/policies-and-guidelines/category/230-2015p#
58. Gibore NS, Bali TA. Community perspectives: An exploration of potential barriers to men's involvement in maternity care in a central Tanzanian community. PLoS One. 2020;15(5):e0232939. https://doi.org/10.1371/journal.pone.0232939
59. Forbes F, Wynter K, Wade C, Zeleke BM, Fisher J. Male partner attendance at antenatal care and adherence to antenatal care guidelines: secondary analysis of 2011 Ethiopian demographic and health survey data. BMC Pregnancy Childbirth. 2018;18(1):145. https://doi.org/10.1186/s12884-018-1775-4
60. Pogoy AM, Verzosa R, Coming NS, Agustino RG. Lived experiences of early pregnancy among teenagers: a phenomenological study. Eur Sci J. 2014;10(2).
61. World Health Organization. A human rights-based approach to health, 2015. https://www.who.int/hhr/news/hrba_to_health2.pdf. Accessed 25 Jul 2021.

Click here to access/download
Supporting Information
S1 File.docx

~~Experiences of social support among unmarried pregnant university students in South Africa.~~Perspectives about social support among unmarried pregnant university students in South Africa

Thandiwe Msipu Phiri[a], Patrick Nyamaruze[b], Olagoke Akintola[c]*

[a]Discipline of Psychology, School of Applied Human Sciences, University of KwaZulu-Natal, Durban, South Africa.

[b]School of Public Health, Faculty of Community and Health Sciences, University of the Western Cape, South Africa

[c]School of Public Health, Faculty of Community and Health Sciences, University of the Western Cape, South Africa

*Corresponding author: oakintola@uwc.ac.za (OA)

Abstract

Background: Pregnant young women in an academic environment are susceptible to stressors associated with unintended pregnancy and academic demands of universities. The challenges they face may be exacerbated by lack of social support, putting them at risk of psychological disorders such as depression. Women who receive social support during pregnancy and postpartum experience less emotional distress and report greater maternal satisfaction. However, very little is known about the perspectives about ~~of~~ ~~male partner~~ social support among unmarried pregnant ~~adolescents and young women~~students in tertiary institutions

Methods: Participants were purposively selected among unmarried pregnant students and those in the puerperal period at the time of the study. We conducted semi-structured qualitative interviews to explore the perspectives of unmarried pregnant students on the type of support that they need during pregnancy and the puerperium; and the period when most support is needed~~with particular focus on the role that their partners play (or do not play) in providing support during pregnancy and the puerperium~~. The data were audio-recorded and transcribed verbatim, then analysed using thematic analysis.

Results: The findings show that social support (emotional, instrumental, informational, and financial) were highlighted as an important resource to cope with stressors during pregnancy and post birth. Emotional support from male partner was the most important type of support needed as it entailed a sense of being loved and cared for. Social support was identified as important throughout the different phases of pregnancy and post birth, with different support needs expressed at each of these phases.

Conclusion: This study identified support needs of unmarried pregnant university students ~~pregnant women~~ in their transition to motherhood. Given the several challenges that they are faced with, unmarried pregnant students need all the available support including male partner

support to enhance wellbeing as they try to cope with academic and pregnancy-related stressors.

Background

Pregnancy marks the beginning of the transition to motherhood, and it is a period of great physical and psychological changes for all women [1]. For a lot of women, it is a period of time that is spent in material and psychological preparation for the arrival of a baby, whose presence brings about several changes in the mother's life. But for some women, this can be a period characterised by considerable physical and emotional stress. The stressors range from physical exhaustion, overwhelming tasks, decreased financial resources, social isolation and depressive symptoms [1, 2]. Research shows that young women experience several stressors during pregnancy due to their age and social context. Stressors such as ~~the~~ academic demands, lack of income and relationship problems with their partners could affect their psychological wellbeing [3, 4]. Therefore, it seems reasonable to suggest that pregnancy is a period when women need a support system to help them cope with the changes as well as preparation for their impending role of motherhood.

Although social support is of great value for all mothers during pregnancy, certain groups of women are especially vulnerable during pregnancy, and these include adolescents, unmarried women, students and women of low socio-economic status [5]. Because of the risky sexual behavior among students, young women in institutions of higher learning have a high risk of unintended pregnancy [6]. There is very little attention focused on students in institutions of higher learning in studies on pregnancy and social support. This could be because they are considered as 'advantaged' as compared to women with low educational attainment [7]. However, the reality is that young women and university students in particular are a vulnerable group considering the circumstances that surround their pregnancy such as their

age, unstable relationships with their baby's fathers, risk of HIV infection due to the sexual culture in universities, unemployment/lack of income, family dependency, academic commitments and having to deal with ~~being~~ pregnancy~~t~~ in an academic environment [8-10].

University students are a population that is susceptible to stress given their academic demands [11]. Most university students are at an age and social context where they are transitioning from adolescence into young adulthood. This can be a difficult period particularly for first year students who struggle with fitting in, maintaining relationships and getting good grades amid the growing demands of life at the university [12].

Pregnancy among students may also carry social stigma and has been described as a traumatic event for a lot of unmarried young women [13]. Further, some young women experience stress due to ~~relationship~~ the instability of their relationships with their partners [8]. Facing these challenges in the midst of academic demands may be daunting. While a lot of students choose to terminate their unplanned pregnancies [14], those that choose to keep their pregnancies remain in need of a supportive environment that favours their psychological wellbeing. Young mothers have less support and face more challenges in terms of adjustment to motherhood as compared to older women [15]. For young mothers in an academic environment, the integration of their roles as students with their new role of motherhood can be confusing and stressful [16]. The challenges they face may be exacerbated by lack of social support, putting them at risk of psychological disorders such as postpartum depression. Furthermore, ~~the findings in~~ one study showed that married women received more support from their partners as compared to unmarried women [17]. This shows that marital status influences the amount of support that pregnant women receive from their partners. Women who receive support from their partners both during pregnancy and in the postpartum period have been shown to experience better mental health outcomes including less anxiety, emotional distress, depressive symptoms and report greater maternal satisfaction [26, 27].

Social support for unmarried pregnant women can come from different sources among them family, friends, male partners and their community. House [18] defines social support as perception and actuality that one is cared for, has assistance available from other people, and that one is part of a supportive social network. Social support, either received from people closest to a person or offered through organizational interventions has a positive influence on pregnancy experiences [19].

Social support is a multi-dimensional construct and has been conceptually divided into four domains: informational, instrumental, appraisal, and emotional support [18]. Emotional support includes any actions of caring towards and improvement of esteem in the recipient of the support. Emotional support for pregnant women could decrease the likelihood of psychological stress disorders and depressive symptoms. Informational support is generally advice and guidance given and, in this case, it could positively influence decisions on issues such as prenatal care, recommended nutritional and healthcare practices and preparation for labour and delivery [20]. Instrumental support involves giving help with tasks or providing material or tangible goods to an individual [20]. Instrumental support such as assistance with household chores and childcare could help expectant mothers cope with physical exhaustion and physically taxing demands which could cause strain, particularly in the last trimester of the pregnancy [21]. Appraisal support is providing evaluative feedback to others and consistent positive feedback during pregnancy and after birth may help normalise the concerns of mothers and build their self-esteem and self-confidence in their parenting role [22]. Support networks have a buffering effect on the stressors associated with pregnancy and motherhood [23]. In the context of maternal and child health, supportive relationships may have a positive effect on pregnant women, giving them a sense of personal control and enhanced feelings of wellbeing that would help them perceive their pregnancy as less stressful [23, 24].

~~Involvement of the male partner has been shown to improve the success of health programmes such as the prevention of mother-to-child transmission (PMTCT) of HIV in many countries [30]. This is particularly true in African settings where men are the key decision makers in relationships. On the other hand, dissatisfaction with partner support among pregnant women is associated with depressive symptoms, worries, and general emotional distress [26, 31].~~

~~While the role of partner support in pregnancy has been extensively studied, the experiences of male partner support among pregnant adolescents and young women in tertiary institutions have received little attention. When it comes to sexual risk behaviour in African tertiary institutions, there are more studies on HIV/AIDS as compared to the risk and consequences of pregnancy among students. The reality that pregnancy is common in universities has often been ignored and the health outcomes of unintended pregnancies in this environment have not been studied extensively.~~ Unmarried pregnant students are a vulnerable group considering the circumstances that surround their pregnancy such as their age, unstable relationships with their baby's fathers, unemployment/lack of income, academic commitments and social stigma. However, the social support needs of unmarried pregnant students in university settings has received little attention. Therefore, the aim of this study is to explore the perspectives ~~experiences of~~about social support among unmarried pregnant students in a university setting. The findings could provide insight into factors that could assist in designing interventions for this population.

Objective

We sought to achieve two objectives in the study:

1) To explore the social support needs of unmarried students in tertiary institutions during pregnancy and the puerperium.

2) To explore the period when most support is needed by unmarried students in tertiary institutions during pregnancy and the puerperium.

Methods

Study design

The descriptive qualitative research design was seen as best suited to answer the research questions in this study. Descriptive ~~Q~~qualitative research design enables researchers to get an insider's view of participants and their experiences in a natural setting [32]. The ~~most frequently proposed~~ rationale for the use of a descriptive qualitative research design is to provide straightforward descriptions of experiences and perceptions (Sandelowski, 2010), particularly in areas where little is known about the phenomenon under investigation. The choice of a descriptive qualitative research design was primarily determined by the type of questions the study sought to address, that is, to gain a deep understanding of the type of ~~male~~ social support needed by unmarried pregnant students. This design was appropriate as it fostered maximum co-operation and closeness between the interviewer and the participants and enabled them to ~~narrate~~ describe their perspectives about pregnancy while studying in the University.

Commented [A1]: Sandelowski M. Whatever happened to qualitative description?. Research in nursing & health. 2000;23(4):334-40.

Participants

Participants in the study were all female students at a public University in ~~the Howard College Campus of the University of KwaZulu-Natal in Durban,~~ South Africa. Participants were recruited if they met a set of inclusion criteria which included that they: were currently registered as students at the University~~ of KwaZulu-Natal~~; were currently pregnant or in the puerperal period (less than six weeks post childbirth) at the time of the study; self-identified

as being unmarried; and were willing to answer questions regarding their perspectives about their pregnancy and support needs.

Because issues relating to pregnancy among young people are sensitive and therefore not openly discussed, it was not easy to recruit participants for the study. Therefore, we used the snowball sampling technique in this study. This is a type of purposive sampling that allowed participants to recommend others in their circle who met the inclusion criteria and were willing to participate in the study [33].

The first author (TMP) initially approached three unmarried pregnant ~~young women~~ students who stayed in the same residence as her. She (TMP) discussed about the study with those students ~~young women~~ and requested them to identify and recommend other students who were unmarried and~~,~~ pregnant or had recently given birth. Next, she (TMP) approached and invited those ~~young unmarried pregnant students women~~ who met the criteria to participate in the study. The first author was a Masters ~~’s~~ student and a young woman who had a pregnancy and delivered a baby while studying for her Masters ~~’s~~ degree. She had received training on qualitative interviewing as part of her Masters ~~’s~~ degree and additional training from the third author (OA) who is a Public Health Promotion and Research Methods specialist~~n. Associate Professor with training in several diverse fields including health promotion, social/economic development, and research methods.~~ She conducted all the interviews drawing on her own pregnancy experiences. ~~to recruit participants.~~ The rationale for that was that some of the participants might be ashamed to express themselves when being interviewed by the opposite sex. It has been shown that the gender of the interviewer can substantially affect the response rate in data collection [34].

Thirty-three ~~More than 30~~ students were invited to participate in the study and seven of them were excluded because they were past the puerperal period. Two of those invited to

participate mentioned that they were not comfortable to discuss their pregnancy experiences and the remaining 24 were recruited~~selected~~ to participate in the study. ~~Current pregnancy and puerperal period were considered because the study sought to explore the experiences of pregnancy.~~

Data collection

Ethical approval for the study was obtained from the University of KwaZulu-Natal Humanities and Social Sciences Research Ethics Committee in Durban (HSS/0584/015M). In-depth interviews were conducted using an interview schedule which contained open-ended questions. The interview schedule was designed to suit the context of the study setting and to encourage unmarried pregnant students to discuss their experiences. The schedule was also reviewed and approved by the third author (OA) who was the research project supervisor. The questions contained in the interview schedule included the socio-demographic variables of participants and their partners, the support needs of unmarried pregnant students, the period when most support is needed and the role of their male partners.

The interviews were conducted in a private and quiet seminar room within the university. This gave them an atmosphere of safety and comfort in which they could talk about their personal experiences considering the sensitive nature of the topic. The interviewer (TMP) made field notes during the interview and then summarized these following the interview. The field notes were thereafter used to develop analytical memos. Each interview lasted approximately 40 minutes. The open-ended questions provided the participants with an opportunity to interact freely and to discuss their ~~pregnancy experiences~~perspectives regarding their pregnancy in detail. Written informed consent was sought and obtained from all the participants before the commencement of interviews. Permission to record the interviews was sought and obtained from participants prior to the interviews. We used a

digital audio voice recorder and the recordings were safely stored in a password protected computer. Data collection was carried out in accordance with relevant guidelines and the participants could withdraw from the study at any point. Although the study was of a sensitive nature, we did not anticipate any negative effects on the participants. However, we had arranged counselling services for the participants if the need arises but none of the participants requested for these services.

Data Analysis

The audio-recorded data was transcribed verbatim by the first author (TMP). The anonymity of the participants was maintained by assigning a pseudonym to each. We chose thematic analysis as the method of analysis for the data because it allowed us the opportunity to conduct iterations through careful reading and re-reading of the data in order to discover underlying meanings and patterns and to produce a detailed account of the phenomenon [35, 36]. As qualitative descriptive research is purely data-derived (), we specifically employed an inductive approach to thematic analysis (37).

Commented [A2]: Lambert, V. A., & Lambert, C. E. (2012). Qualitative descriptive research: An acceptable design. Pacific Rim International Journal of Nursing Research, 16(4), 255-256.

~~Next, the~~ The first author (TMP) performed the initial round of coding following the six steps described by Braun and Clark [37]. The first step in the process was familiarization with the data by reading and re-reading the transcripts to make summaries. Secondly, she (TMP) generated initial codes and this step was followed by identifying the emerging themes, which is the third step. Fourthly, TMP reviewed potential themes, particularly checking for inconsistencies and whether the themes overlapped. Next, the themes were defined and named. Lastly, a report was produced using quotations of what the participants said to illustrate the themes [37].

It is important to note that this process was not straightforward as presented rather it was iterative. The initial coding was then reviewed and modified by the third author (OA) after which the second author (PN; a post-doctoral fellow and researcher), conducted a general review of the themes. All the authors revised, finalized and agreed upon the themes.

Findings

In total, twenty-four ~~unmarried~~ students who met our criteria participated in the study (Table 1). Among these, twenty-one were pregnant and three were in the puerperal period. All participants, including those who were in the puerperal period had disclosed their pregnancy to significant others.

Participant pseudonyms	Age	Gestational stage	Number of previous pregnancies
Mbali	20	Seven months pregnant	None
Lindiwe	23	Eight months pregnant	None
Busi	19	Four months pregnant	None
Samantha	19	Three months pregnant	None
Nomthandazo	19	Four weeks puerperal	None
Bongiwe	21	Three months pregnant	None
Bella	26	Six months pregnant	One
Kholeka	21	Eight months pregnant	None
Zevile	23	Five months pregnant	None
Rachael	22	Five months pregnant	None
Thuli	21	Eight months pregnant	None
Njabulo	24	Six months pregnant	None
Khethiwe	24	Four months pregnant	One
Minenhle	20	3 weeks puerperium	None

Barbara	21	seven months pregnant	None
Khanyisile	22	nine months pregnant	One
Thandeka	20	four months pregnant	None
Lerato	21	Five weeks puerperium	None
Zinhle	23	Six months pregnant	None
Thuli	20	Three months pregnant	None
Liyana	26	Seven months pregnant	One
Siyanda	19	Four months pregnant	None
Nandi	23	Seven months pregnant	None
Lindiwe	22	Three months pregnant	None

A summary of the characteristics of the participants are provided in Table 2. Four of the participants had one previous pregnancy whil~~est~~ it was the first pregnancy for the remaining twenty participants. The findings are organized into four main themes and ten sub-themes identified from the analysis of the data. The four main themes are: Support needs of unmarried pregnant students; period when the most support is needed; male partner support required; and health systems role on social support and influence on male partner support.

Table 2 Summary of the socio-demographic details of participants

**Characteristics**		**Number of participants (Percentage)**
**Age of participants**	15-19	4 (16.7%)
	20-24	18 (75%)
	25-30	2 (8.3)
**Year of study**	1st year	7 (29%)
	2nd year	9 (37.5%)
	3rd year	5 (21%)
	Honours	3 (12.5%)
**Gestational age**	1st month-birth	21 (87.5%)
	Birth-6 weeks puerperium	3 (12.5%)

**Number of previous pregnancies**	None	20 (83%)
	1	4 (17%)

Support needs of unmarried pregnant students

Support during pregnancy was perceived to be very important among all the participants. All ~~Social support is a part of pregnancy that all~~ the participants said they could not do without social support. The support needs of the participants were similar in many ways but sometimes unique to their situation. The following ~~were the~~ three sub-themes emerged~~ing~~ from the interviews.

Emotional support

~~Many~~ Some of the participants indicated that they felt a sense of shame because of their pregnancy. ~~Several~~ Other participants mentioned experiencing feelings of shame and embarrassment at university ~~school~~ and in their communities as a result of their pregnancy. This was exacerbated when people would stare at them and that was considered to be a negative attitude.

> Some of my friends ignore me due to the pregnancy because they don't wanna walk with me to school and I also see myself as totally different from them. At first, I felt so neglected now I have accepted that am pregnant and things won't be the same. I usually walk alone. Some friends will support me, some will not. [Lindiwe, honours student]

Emotional support was considered to be the most important type of support among participants. ~~Several~~ Some participants expressed their need for a 'listening ear' and the

desire to be understood and not be judged for getting pregnant. Their accounts showed that they experienced feelings of sadness and loneliness and considered themselves vulnerable because they got pregnant whilst pursuing their studies. Personal vulnerabilities and situational constraints led to loneliness among the participants. Due to loneliness, the ~~young~~ unmarried students~~women~~ in this study expressed a need for social relationships that would offer constant assurance that someone was there for them. Therefore, to minimize these feelings of loneliness, emotional support was considered to be the most important type of support that they needed to receive among all others.

> It feels good to have people around and knowing that you are loved. Although you made mistakes - not that a baby is a mistake – but being pregnant while you are studying, being something that you have not planned obviously. So knowing that although you have made that mistake, but people haven't abandoned you. People around you like your loved ones and your family- that they still love you [Slindile, 20 years old, 2 weeks pueperal].

In comparison to financial support, receiving emotional support and having people who were going to be there for them in times of sadness was said to be important and it made their pregnancy easier to handle.

> I just don't need money in that kind of way. The kind of support that I would really like, that I would really expect is for people to be there for me, to give me that much attention, to be there when I feel sad [Rachael, six months pregnant]

For some participants, having someone available to offer emotional support was important regardless of who was offering it. They reported that the availability of emotional support made a huge difference in their lives regardless of its source because their greatest need was

to feel loved and cared for. Having people to talk to and understand them, without judging them was important for participants.

> A person who is pregnant just needs support, love - no matter how bad the situation is or how it has happened. Having people to talk to and that understand, are really there for you, lending their ear more than their mouth, people to talk to [Samantha, seven months pregnant].

Instrumental support

Receiving help with tasks and help to manage their time was an important support need of ~~most~~ some participants. Their narratives revealed that instrumental support was most needed at the end of the pregnancy because as the pregnancy progressed, it became more difficult to be mobile. Therefore, they needed people to be around and help them with chores, academic tasks, accompanying them to certain places especially hospitals or going to buy them food and groceries. One participant noted that she needed company every time she was going to the health facilities unlike before she got pregnant.

> If I was sick and I wasn't pregnant I'd be like, "no please, don't come, I'll go by myself". Now! [that she is pregnant], Now I can't go to the clinic by myself, I'll just call anyone, call my sister or my aunt, she's always there she's like no I'll take you. If it [support] had to stop now, I don't know what I would do [Amahle, 18 years old, seven months pregnant].

Another participant revealed how overwhelming the tasks had become in her last trimester of pregnancy and the importance of receiving instrumental support during this period.

> When you are nearing your end [approaching delivery], you have so much to do. You have got school things to do, you have got doctors' appointments consistently one

> after the other, you have to buy this, get this, get that, that’s when you need a more rigid [solid] support system [Khanyisile, Honours student, nine months pregnant]

These excerpts show that apart from the need to feel better about their situation, unmarried pregnant students need help with tasks to relieve them of physical stress. In addition, receiving help with tasks also had an effect on their emotional wellbeing.

Informational support

The narratives of the participants in this study revealed that young women, particularly first-time mothers are usually inexperienced with pregnancy and childbirth and are in need of advice and information. Some ~~P~~participants reported that they needed people around them that could help them with information about pregnancy, childbirth and how to care for their baby.

> I do not have experience on how to bath the baby, feed the baby well when it’s crying, what’s wrong. They [people] should tell me what to expect if am not figuring out, they must assist me [Nomthandazo, nine months pregnant].

Other participants ~~Young women~~ also indicated that the needed~~need~~ information on what to expect during the different stages of pregnancy and childbirth. One of the participants who had a previous pregnancy explained the difficulties of having a first pregnancy without adequate knowledge of the accompanying physical changes.

> At the beginning of pregnancy, maybe you are not sure what is happening to you, may be for some people it’s the first time you don’t know what is happening, all the sickness. At the end that’s exactly what’s happening to you, breaking water and all that. Some of the things you wouldn’t know so someone should tell you [Naledi, nine months pregnant, second pregnancy].

Participants require guidance on pregnancy with respect to proper nutritional practices, adequate prenatal care and preparation for labour and delivery. ~~This is because inadequate nutrition in pregnancy is a risk factor for low infant birth weight, a major cause of neonatal mortality [38].~~ Due to lack of information on proper nutrition practices, some participants risk having low birth weight infants as illustrated below:

> After I fell pregnant, I did not eat. I don't usually eat. I didn't know that when you fall pregnant you have to change your lifestyle and do things in a certain way. It was a shock when I went for a scan when I was five months pregnant and they said my baby looks like he's two or three months, he was underdeveloped. So, the doctor said "it's either your child is gonna be disabled or when you give birth, you're gonna have to stay in hospital for a long time cause he's really small" [Samantha, 19 years old, six months pregnant].

The participant's narrative ~~narration from this participant~~ highlights the challenges of poor nutritional intake during pregnancy. ~~is excerpt is an example of the risks associated with inadequate nutrition and late initiation of prenatal care, which is common among women with unintended pregnancy.~~

Period when the most support is required

When asked about the specific period during pregnancy when they feel the most support is required, participants had different views and four ~~main~~ sub-themes arose.

At the beginning of pregnancy

~~A few~~Some of the participants said they needed the most support, particularly emotional support at the beginning of the pregnancy. This was a period when they had just found out about their pregnancy which was something new to them. At the same time, they were still

dealing with acceptance of the pregnancy whether personally or in their relationship. Therefore, they indicated that at this initial stage of pregnancy, they required some form of assurance from someone that they were going to be supported.

> I think I needed more support at the beginning. It was something new to me. I kind of needed someone to hold my hand and say ‘it’s gonna be fine, I’m gonna be therefore you, don’t worry, we’ll get through this together’ [Thandeka, 19 years old, four months pregnant].

In addition, this was the period when they were likely to be hiding the pregnancy from their family when in actual fact, they needed their support. Therefore, they found it difficult to share their stressors with others.

> It was difficult before I told my parents because you know when nobody knows at home and you start experiencing these things [disagreements/fights] with your boyfriend and you get pissed, like you wanna tell someone but you can’t. [Amahle, 18 years old, seven months pregnant].

Therefore, it is important to receive support at the beginning of pregnancy for better maternal and new-born health outcomes. The different experiences that the participants had in their pregnancies determined what they perceived their support needs to be and the period that they were required.

Throughout pregnancy

~~Most of the~~Other participants said they required support throughout their pregnancy and into the puerperium because they felt their support needs were not going to change over the course of the pregnancy. Therefore, they said that they needed to receive support through every period of the pregnancy.

> Throughout the pregnancy you need support because there are times where you feel like I wish I could just give up everything like you know, school cause sometimes you find it frustrating, and you don't wanna deal with it. I think you need that pillar of strength every day; someone that will be calling you and asking you 'How are you doing? How's everything? How's the baby?' Like I think every day during pregnancy even after pregnancy [Zinhle, 25 years old, six months pregnant].

Towards the end of pregnancy

Some participants felt they needed more support as they approached their delivery date. The support required at this stage was mostly instrumental support. ~~Participants~~ They indicated that it was difficult to balance antenatal care (ANC) ~~clinics~~ schedules at the public clinics and academic obligations in the final trimester. This suggests that in the last trimester of pregnancy, students experience a task overload. For this reason, they need instrumental support in the final trimester of pregnancy.

> It was at the end [when she needed more support]. When you feel like the baby is about to come, you have to prepare for everything. With schoolwork it's very hard. Because at the end, I had to attend clinics more than before, like almost every week. So, the time you are attending clinics and everything, you are using up the time for studying [Slindile, 20 years old, two weeks puerperal].

One participant expressed her need to always have someone with her so that she would be well prepared in the event of labour.

> Mostly it's at the end when you'll be delivering (when she needs the most support) because at that time you have a lot of complications, you need someone to look after you because you can deliver anytime. You need to have transport that will take you to

> the hospital immediately and always bring your card so that you are well prepared [Thuli, 19 years old, eight months pregnant,].

Support post delivery

Young women usually require support and guidance after childbirth and in the case of students; they need support with childcare as they continue with their studies. Except for two who were not sure, ~~most~~ other participants expressed their intention to continue with their studies after their child is born. When asked about childcare, ~~most~~ some participants' responses were that their mothers or female guardians were going to look after the baby upon delivery. In cases where mothers were unavailable due to distance or employment, they were still going to offer support by paying a helper to look after the child. Therefore, parents were also going to provide financial support to a large extent. Young women in this study relied heavily on their own mothers and depended on them for childcare.

> I'll still be in Durban when I give birth cause am giving birth in September. So my mom will travel here. My mom said "give me the child then you go back to school" and she's gonna take care of the baby...In terms of finances my mom will support me. I don't know about my child's father whether he'll be there or not [Thandeka, second year student].

Those who did not have mothers (due to death) or those whose mothers worked faraway would receive help from other older female members of the family such as aunties and sisters.

> My mother`s sister, she lives in Maritzburg [Pietermaritzburg], she did say she can take care of baby once the baby is born so that I can go back to school and study cause that is what I want to do. She was the one who volunteered to do that [Nomthandazo, first year student]

When participants were asked about financial support, some of their accounts revealed that providing for the child's welfare was the father's responsibility.

> The baby needs so many things so as to grow in a good state; nappies, warm clothes, milk, formula [baby food] since am not going to breastfeed so financially it will be much required from him. [She gives an example]-cause my cousin gave birth in April and the father of the baby hasn't given her a single cent since she gave birth, since she fell pregnant, she hasn't received any money from the baby's daddy and telling me things are difficult, so financially it is so important [Thobile].

Male partner support needed

One universal theme that emerged in all the interviews with respect to support needs was receiving support from the male partner. Male partner support was considered to be the most important source of support that unmarried pregnant students required. The lack of emotional support from male partners during pregnancy was seen to have grave consequences for both the mother and her unborn baby. These include depression, resentment and abortion.

> As much as you are the one carrying the child, you can't go through everything by yourself. I think if you don't have your partners support, you can end up having resentment towards him, including the child - somebody who has no contribution towards the baby is painful [Khanyisile, Honours student].

Partner's emotional support

Receiving emotional support from partners was the biggest support need among young women. When compared to other support needs, ~~most~~ some participants reported that they would rather have their partner to be there for them emotionally than financially. The

emotional support required by ~~some of ~~these participants ranged from the simplest acts of care like being there to talk to them or calling them every day.

> I would like to be supported emotionally because money doesn't buy love. It is the tender loving care that is most important. I appreciate that cause that is what is important, that is what I prefer, it's what I need [Naledi, nine months pregnant]

Other ~~P~~participants said that their partners needed to be their greatest source of support because they were the reason why they got pregnant. Therefore, they needed to take responsibility for 'what they did' and 'compensate for their mistake'. This means that male partners were expected to play a major role in providing support and relieving emotional distress.

> You know when you are pregnant, I think you expect a lot when you know that this person is the baby's father, it's like you want them to do more. You want them to try anyway and compensate for doing what they did to you, because if it wasn't for him I actually wouldn't be pregnant so he has to be there for me [Zinhle, second year student, six months pregnant]

There was a general view among some participants that the relationship with their partner should remain as it was before pregnancy or improve with the prospect of a child. However, it was found that in a lot of cases, relationships became weak as the pregnancy progressed. In some cases, the pregnancy led to the end of the relationship. ~~Participants ~~Others revealed that relationships become more about the wellbeing of the unborn baby and less about the romantic relationship between the partners. Some ~~P~~participants reported feeling neglected and expressed longing for a romantic relationship as they did before the pregnancy.

> I don`t want him to treat me as the mother of his babies, I want him to treat me also as his girlfriend. Now we usually talk about the baby a lot. We don't talk about us. I

> want us to date like we used to before, go out with him, watch some movies and get a lot of support from him [Thuli, second pregnancy].

Partner's instrumental support

Having a partner who is available to provide material goods and help with tasks was important among the participants. Instrumental support such as having the partner's company when they go places and buying them foods that they crave for was considered to be an important support need. The smallest acts of help with tasks were considered to be of great importance even in cases where partners could not offer financial support.

> Sometimes people ask questions about him [her partner] when you are walking alone. I need the support. If he was not there for me, it would have hurt me you know, to know that people are laughing at me [Thobile, eight months pregnant].

One participant complained about her partner who is not usually willing to help her with tasks.

> Sometimes he'll be sleeping [when he visits her in her campus room] and I have to do my schoolwork then when I ask him to do something like can you please get me some water, sometimes he's gonna be like he's tired or when I want him to do something for me he's gonna complain [Zinhle, six months pregnant].

Instrumental support was considered to be crucial particularly in matters involving doctors' appointments and preparations for the arrival of the baby. In cases of medical emergencies, partners were required to be present. Going to the hospital together was considered to be an important aspect of instrumental support as it assured them that they were not going through their pregnancy alone.

> Before I fell pregnant, I really didn't notice anything, and it didn't matter. Now it's more like I want more, more time with him. Sometimes when you go to the clinic and you see people with their boyfriends and then I'll be thinking like, where's mine? I also want him to be here and experience this together. [Amahle, seven months pregnant].

Partner's financial support

All the participants said that their tuition ~~school fees~~ are paid by their parents. Therefore, the financial resources expected from male partners were for issues concerning the pregnancy. Financial needs from partners included money to buy food that they crave, maternity clothes, hospital bills and items for the baby. However, more financial support was expected after delivery as compared to during pregnancy.

> I need him not to run away from his responsibilities when the baby is here, that's all I'm just hoping for, that he'll be a father and support his child [Lindiwe, 22 years old, eight months pregnant].

Financial support did not always mean receiving money from their partners. Having their partners buy or pay for their needs was also regarded as financial support.

> Well, my partner is not good at giving money and all that, but he can do everything and anything to support me, he put me on his medical aid [medical insurance], he can buy me anything that I crave for, but giving money no. So, he supports me, yes [Naledi, second year student].

The payment of damages (Inhlawulo) was considered to be a form of financial support as it was money that could be used by the family to support the pregnant women. This study found that some~~most~~ of the participant's partners had not paid this fine to the young woman's

family. In the current study, men who paid damages to their partner's family were perceived to be more responsible and could be entrusted with caring for them. The payment of damages was observed to have a calming effect on angry parents and associated with a more positive attitude towards the pregnancy. Therefore, it was considered as a type of support that was required of partners to assure the family that he would honour his responsibility.

> My sister is the one who advised me about (partner) paying the damage in December because that is when my dad is coming from Joburg. I want him (father) to know that even if he chases me [from his house?], he will know that everything was done perfect and I'm not staying with, what can I say, a thug or anything. I want him to know that am staying with the real man [Mbali, 20 years old].

On the other hand, men who did not pay damages were seen as untrustworthy.

> To be honest sometimes I think he is going to run away, since he hasn`t paid damages and he can see the other girls, and forget about me you know, you know how boys are [Nomthandazo, nine months pregnant].

Health systems role on social support and influence on male partner support

Health professionals are able to contribute to the psychological wellbeing of women during pregnancy and during the postpartum period by offering support. When asked about their experiences at ~~antenatal~~ ANC clinics, some participants reported that the health staff at the clinics had a negative attitude towards them. They felt stigmatized because of their age and status as students, and this was because of what the nurses would say about them.

> Ooh at first, I had hell, like the nurses ask you basic questions: "how old are you?" Then when you say 18 then everybody is like "hau?" then instantly when you say you

> go to Howard College everybody assumes like “no wonder, it’s expected”. [continues to mimic] “Where’s the baby’s daddy?” Am like “he’s working”. They’re like “yeah, you have a working baby’s daddy because that’s what you do”. They weren’t really saying it to my face, but you can hear them gossiping [Amahle, 18 years old]

When asked about how the health systems influence male partner support among pregnant ~~women~~students, particularly partner ANC attendance, almost all the participants said they had never attended an ANC session ~~antenatal clinic~~ with their partner. Some participants said their partners did not want to go to the clinics with them because of the female dominated environment at the clinic and the amount of time spent at the clinic. In addition, nurses did not seem to be concerned about the absence of partners.

> I wanted him to be there, but he suggested that no, he will remain behind, or he will go with me and leave me there. Personally, I understand why he doesn’t want to stay with me. Clinics are just something else, many people and you wait for hours. The nurses don’t even ask about my partner. [Thobile, 19 years old]

Some participants explained that ~~said~~ they were not attending ~~antenatal clinics~~ANC in public clinics but instead were consulting specialists like gynaecologists and obstetricians in private hospitals to monitor their pregnancy. This was mostly because they felt that they needed to be checked by specialists who were not readily available in public clinics.

> With my partner first of all he said he doesn’t want me to go to clinics and all that, so he put me on his medical aid [medical insurance]. He didn’t want me to be going to the clinics. There’s nothing wrong with the clinic but for me he preferred the gynaecologist who is gonna do everything [Naledi, second pregnancy]

~~A few of the~~Some participants mentioned that their partner did accompany them to the clinic on first visit but did not do so on subsequent visits. While they had the desire to have their

partners come to the clinics with them, they mentioned that they did not see the purpose because nurses at the clinic did not involve men in any of the activities.

> He came with me to the clinic on my second appointment, when I was about four months. It means a lot to have someone with you. But they didn't ask him any questions or asked him to do any tests [Slindile, new mother].

Overall, it was established that most ~~young~~ unmarried pregnant students ~~women attend~~ seek ~~antenatal care (~~ANC~~)~~ in public clinics and hospitals ~~facilities~~alone without the support of their male partners. ~~only because it is a requirement for accessing delivery services at public hospitals. Women who do not attend ANC can only deliver from a private hospital, which none of the participants could afford except for those whose partners or parents were paying for the service.~~ The participants generally considered ANC to be unfriendly ~~for young unmarried women, especially students~~. In addition, partner attendance was not specifically encouraged or recommended by healthcare workers.

Discussion

Unmarried ~~P~~pregnant students need emotional, instrumental, informational and appraisal support. Research on social support has consistently found that the absence of social support is a significant risk factor for antenatal and postnatal depression and anxiety among pregnant women [39, 40]. This study sought to explore the support needs of young women during pregnancy and the puerperium. ~~Despite the fact that people who have unintended pregnancy confront various stressors, few studies have explored the sources of support pregnant students perceive as the most important during and post pregnancy.~~ Our findings suggest that the socio-cultural context of pregnancy among young women results in social stigma. Unmarried ~~P~~pregnant students are stigmatized in the academic environment as well as in the community. This study found that ~~pregnant~~ these students are sometimes ~~abandoned~~ ignored by their

peers because of the pregnancy. This leads to social isolation, a common effect of pregnancy among young people [41]. Social isolation has been associated with loneliness, emotional distress and depressive symptoms. Experiencing depressive symptoms in pregnancy has consequences for maternal and child health outcomes because it is associated with low birth weight [42].

Four different types of social support (emotional, instrumental, informational, and financial) were highlighted as an important resource to cope with stressors by all participants. Although financial support is usually categorised as instrumental support, in this study we categorised it as an independent form of support as it was a salient theme in the study. Emotional support was described as having people who are available to share their feelings with, encourage them and show acts of caring towards them. Instrumental support expressed by participants included receiving help with tasks such as going for medical check-ups. The participants in our study indicated that they require informational support, which is receiving information on pregnancy, childbirth and how to take care of a new-born baby. Lastly, financial support was needed to buy food, to cover transport costs and medical expenses and to prepare for the arrival of the unborn baby. All these types of support play a role in fostering positive maternal and child health outcomes.

An important finding of our study is that, among the different types of social support needed, emotional support was deemed more important by the participants. Pregnancy was associated with loneliness and fear of being judged for falling pregnant within an academic setting. The young women desired to be listened to and loved more especially because of their condition which was associated with various stressors. Although a previous study reported on the importance of emotional support during pregnancy, it was not ~~rated~~ regarded as the most important type of support needed [43]. The provision of re-assurance, feeling valued and sympathetic listening result in feelings of comfort and is associated with decreased levels of

depressed mood and anxiety during pregnancy and postnatal [44]. In line with Cohen and Will's [45] stress buffering hypothesis of social support, unmarried pregnant students perceive emotional support to buffer the effects of psychological distress during pregnancy. Previous studies have also shown that perceived social support may have a positive effect on pregnant women causing them to perceive their pregnancy as less stressful and providing a resource for effectively dealing with stressful situations such as labor [46, 47]

For unmarried pregnant students, who usually stay at university residences, instrumental support was perceived as important in assisting to balance the academic demands and pregnancy-related demands. The pregnancy came with additional responsibilities such as regular visits to the clinic and performing previously manageable tasks was perceived as daunting as a result of the pregnancy. A study in Ghana among women with new-borns and various community stakeholders found that women were dependent on network members for instrumental support to get them to a health facility for childbirth [48]. However, instrumental support seemed unavailable to pregnant women residing at university residences as they did not stay with their families. The narratives of the participants revealed that young women, particularly first-time mothers were usually inexperienced with pregnancy and childbirth and need advice and information. In a previous study, 70% of first-time pregnant women indicated that they needed information about pregnancy and childbirth [49]. The participants expressed a lack of knowledge regarding pregnancy-related sickness, delivery process, and dietary needs as well as taking care of the newly born child. A recent study among adolescent pregnant women has shown knowledge deficit on the benefits of ANC and nutrition during pregnancy, that may contribute to poor pregnancy outcomes [50].

Our study sought to understand the stage at which social support was deemed more important by the unmarried pregnant students. Social support was identified as important at different phases of pregnancy. In the first few months of pregnancy, the expectant mother develops

new and often uncomfortable physical and emotional symptoms such as nausea, feelings of uncertainty and increased emotional expressions [51, 52]. In the early stages of the pregnancy, young women desired support from significant others as they were still in the process of embracing the pregnancy and thus required acceptance and assurance that they would be supported throughout the whole pregnancy experience. Towards the end of the pregnancy, informational support was deemed more important to help balance between academic demands and regular visits to the clinic. More so, the narratives from most of the participants indicate that support was necessary throughout the whole period of pregnancy and after giving birth. The various stressors faced by unmarried pregnant students came at different stages of the pregnancy and were accentuated by the challenging academic environments characterised by high workload, meeting deadlines and socio-economic issues [53, 54]. Thus, an enduring and reliable support network was essential.

While support from different sources is important for unmarried pregnant students, participants identified male partner support as their most important source of support. Unlike community-dwelling women in Bangladesh who cited mothers and sisters-in-law as top sources of emotional support [43], participants desired their partners to be emotionally available and care for them since they were partly involved in conceiving the child. Male partners are expected to take responsibility and be available to offer emotional support to young women throughout pregnancy and after childbirth. The availability of support from male partners gives women a more positive attitude towards their pregnancy and assures them that they are not alone thereby reducing emotional distress [27]. Partner support is an important protective factor throughout ~~pregnancy and~~ conception and pregnancy and our study corroborate~~s~~ these findings. Importantly, participants did not want to be perceived by their partners as mothers only, but as lovers, as that entailed emotional connection. This type of support and relationship status was deemed more important than financial support. A study

among couples indicated that more prenatal shared leisure predicted higher marital love and less conflict when the baby was 1 year old [55]. Even though participants in the current study were unmarried it seems that they would benefit from spending more quality time with their partners. A previous study showed that male partners who did not approve an unplanned pregnancy were less likely to be emotionally available increasing the risk of psychological distress in women [3]. In instances where emotional support from male partners is not feasible, there is a need to design interventions that will assist unmarried pregnant students in identifying alternate sources of support such as counselling support, seeking social support from friends and their social circles as well as through the social media.

Partners' instrumental and financial supports were also considered to be ~~the second most~~ important ~~after emotional~~sources of support. The participants wanted to be accompanied to clinic visits and experience the pregnancy process with their partners. In previous studies, male involvement in maternal and new-born health has been associated with a variety of improved health outcomes including increased maternal access to antenatal and postnatal services, improved maternal mental health and reduced postpartum depression [56, 57]. ~~Withi~~In ~~the~~ some African cultures, payment of damages is perceived as a form of acceptance of paternity [58]. This process was perceived by the participants to give a form of dignity to her family and some assurance ~~ed them~~ that the father was prepared to take care of the child. However, similar to ~~as shown in~~ a previous study ~~ies~~, expectations of partner support (emotional, instrumental and financial) are not usually met resulting in disappointment, conflicts with their partners and sometimes difficult break ups [8]. The current study found that experiencing an unintended pregnancy can lead to the disruption of a relationship. These findings complement the results of research showing that unplanned pregnancies are accompanied by family disruption and a decline in the marital quality and the couple's

relationships [3, 59]. This results in emotional distress for unmarried pregnant students who may have to experience pregnancy without the support of their partner.

Unmarried ~~P~~pregnant students experienced social stigma at health facilities. This finding is consistent with the results from a previous study among healthcare providers in Ugu district in southern KwaZulu-Natal, South Africa which showed that health personnel exhibit a negative attitude towards pregnant young women [60]. The South Africa Department of Health guidelines recommend that pregnant women seek ANC as soon as they suspect a pregnancy and encourages all pregnant women to attend at least eight ANC visits []~~Attendance of ANC is mandatory to access delivery services in South African state health facilities~~. However, the experience of social stigma at public health facilities is likely to result in ~~young women~~ unmarried pregnant students shunning ANC, yet most of them cannot afford to use private healthcare services. Young women in our study also expressed the desire to have male partners attend ANC visits with them. However, it was reported that this does not usually happen due to the amount of time spent at ANC clinics, the female dominated environment and the negative attitudes from the nurses. These findings add to previous research which demonstrated that the main barrier to male participation in ANC was the healthcare facility environment [61]. Further, during ANC~~antenatal~~ visits, nurses only ask about the existence of male partners but do not necessarily encourage partner attendance of ANC. Therefore, there is minimal influence from the healthcare professionals on emphasizing partner attendance of ANC, their perceived roles and benefit to the mother and the baby. Health systems have a role to play in fostering supportive relationships between pregnant women and their partners. Partner support and involvement in maternal healthcare has been shown to enhance maternal health outcomes [62].

Among women of all ages, pregnancy is associated with a myriad of stressors. In the contexts of academic institutions, pregnancy-related stressors are likely to add to the academic

Commented [A3]: National Department of Health, South Africa. Guidelines for Maternity Care in South Africa. A Manual for Clinics, Community Health Centres and District Hospitals. 4th ed. Pretoria: NDoH, 2015:172. http://www.health.gov.za/index.php/2014-03-17-09-09-38/policies-and-guidelines/category/230-2015p#

challenges faced by female students (). Absence or lack of emotional social support from significant others, particularly partners may exacerbate the stressors of pregnancy among students. Tertiary institutions should partner with established social initiatives offering services for men in the country to engage and mentor young men one-on-one and in groups about the importance of the father's role in the child's development and how to best support their partners to improve pregnancy outcomes, irrespective of relationship status. Culturally sensitive health promotion programmes targeted at 'father inclusive' practices and behaviour change communication programmes to stimulate social support may have positive changes in terms of the supportive role of partners. Implementors of such programmes should consider adopting innovative outreach strategies that appeal to young men's interests and hang-out spots, such as bars and sports arenas.

Male involvement ~~These~~ programmes can be integrated in ANC whereby services are restructured to accommodate male partners to ensure a sense of involvement and responsibility. However, for these programmes to work, the ANC environment should be user friendly for young women and their partners. A rights-based approach must be consistently adopted when delivering maternal healthcare [63]. ~~The government should provide more~~ ~~t~~Training and ~~continuing~~ on-going education should be prioritised for healthcare workers ~~antenatal health personnel~~ regarding the provision of positive, respectful and supportive non-judgmental antenatal and postnatal care to their clients irrespective of their age or marital status. Pregnancy at universities is not uncommon and having clinics at university campuses equipped with comprehensive ANC may overcome some structural barriers such as the burden of transport costs and long waiting times at public clinics.

Commented [A4]: Pogoy, A. M., Verzosa, R., Coming, N. S., & Agustino, R. G. (2014). Lived experiences of early pregnancy among teenagers: a phenomenological study. European Scientific Journal, 10(2).

Conclusion

This study identified support needs of pregnant women in their transition to motherhood. Among the support needs of unmarried pregnant students, emotional support is perceived to be the most important type of support ~~among pregnant students~~. Male partners appear to be the most sought-after source of emotional support for unmarried pregnant students as they desire to experience the emotional connection that existed before the pregnancy and fear the possibility of abandonment. Given the several challenges that they are faced with, unmarried pregnant students need all the available support to enhance wellbeing as they try to cope with academic and pregnancy-related stressors.

Acknowledgements

The authors would like to express their sincere thanks to the participants for the value they added to the study

References

1. Erfina E, Widyawati W, McKenna L, Reisenhofer S, Ismail D. Adolescent mothers' experiences of the transition to motherhood: An integrative review. Int J Nurs Sci. 2019;6(2):221-8. https://doi.org/10.1016/j.ijnss.2019.03.013
2. Schetter CD, Tanner L. Anxiety, depression and stress in pregnancy: implications for mothers, children, research, and practice. Curr Opin Psychiatry. 2012;25(2):141-148.
3. Barton K, Redshaw M, Quigley MA, Carson C. Unplanned pregnancy and subsequent psychological distress in partnered women: a cross-sectional study of the role of relationship quality and wider social support. BMC Pregnancy Childbirth. 2017;17(1):1-9. https://doi.org/10.1186/s12884-017-1223-x
4. Atuyambe L, Mirembe F, Annika J, Kirumira EK, Faxelid E. Seeking safety and empathy: adolescent health seeking behavior during pregnancy and early motherhood in central Uganda. J Adolesc. 2009;32(4):781-96. https://doi.org/10.1016/j.adolescence.2008.10.012
5. Colciago E, Merazzi B, Panzeri M, Fumagalli S, Nespoli A. Women's vulnerability within the childbearing continuum: A scoping review. Eur J Midwifery. 2020;12(4):18. https://doi.org/10.18332/ejm/120003
6. Mbelle N, Mabaso M, Setswe G, Sifunda S. Predictors of unplanned pregnancies among female students at South African Technical and Vocational Education and Training colleges: Findings from the 2014 Higher Education and Training HIV and AIDS survey. S Afr Med J. 2018;108(6):511-6. https://dx.doi.org/10.7196/samj.2018.v108i6.12744

7. El-Adas A. The resolution of unintended pregnancy among female students at the University of Ghana, Legon. 2007, Regional Institute for Population Studies: University of Ghana, Legon
8. Govender D, Naidoo S, Taylor M. "I have to provide for another life emotionally, physically and financially": understanding pregnancy, motherhood and the future aspirations of adolescent mothers in KwaZulu-Natal South, Africa. BMC Pregnancy Childbirth. 2020;20(1):1-21. https://doi.org/10.1186/s12884-020-03319-7
9. Nkosi KB, Makhene A, Matlala S. Educational challenges as experienced by pregnant student nurses at a college in Mpumalanga. Curationis. 2019;42(1):1-7. https://doi.org/10.4102/curationis.v42i1.1880
10. Kebede A, Molla B, Gerensea H. Assessment of risky sexual behavior and practice among Aksum University students, Shire Campus, Shire Town, Tigray, Ethiopia, 2017. BMC Res Notes. 2018;11(1):1-6. https://doi.org/10.1186/s13104-018-3199-7
11. Pascoe MC, Hetrick SE, Parker AG. The impact of stress on students in secondary school and higher education. Int J Adolesc Youth. 2020;25(1):104-12. https://doi.org/10.1080/02673843.2019.1596823
12. Mudhovozi P. Social and academic adjustment of first-year university students. J Soc Sci. 2012;33(2):251-9. https://doi.org/10.1080/09718923.2012.11893103
13. Moseson H, Mahanaimy M, Dehlendorf C, Gerdts C. "… Society is, at the end of the day, still going to stigmatize you no matter which way": A qualitative study of the impact of stigma on social support during unintended pregnancy in early adulthood. PLoS One. 2019;14(5):e0217308. https://doi.org/10.1371/journal.pone.0217308
14. Wang H, Long L, Cai H, Wu Y, Xu J, Shu C, et al. Contraception and unintended pregnancy among unmarried female university students: a cross-sectional study from

China. PLoS One. 2015;10(6):e0130212. https://doi.org/10.1371/journal.pone.0130212

15. Wahn EH, Nissen E. Sociodemographic background, lifestyle and psychosocial conditions of Swedish teenage mothers and their perception of health and social support during pregnancy and childbirth. Scand J Public Health. 2008;36(4):415-23.
16. Birkeland R, Thompson JK, Phares V. Adolescent motherhood and postpartum depression. J Clin Child Psychol. 2005;34(2):292-300. https://doi.org/10.1207/s15374424jccp3402_8
17. Figueiredo B, Bifulco A, Pacheco A, Costa R, Magarinho R. Teenage pregnancy, attachment style, and depression: A comparison of teenage and adult pregnant women in a Portuguese series. Attach Hum Dev. 2006;8(2):123-38. https://doi.org/10.1080/14616730600785686
18. House J. Work, stress and social support. Menlo Park, CA: Addison-Wesley; 1981.
19. Raman S, Srinivasan K, Kurpad A, Dwarkanath P, Ritchie J, Worth H. 'My Mother… My Sisters… and My Friends': Sources of maternal support in the perinatal period in urban India. Midwifery. 2014;30(1):130-7. https://doi.org/10.1016/j.midw.2013.03.003
20. Cofie LE, Barrington C, Sodzi-Tettey S, Ennett S, Maman S, Singh K. A qualitative study of women's network social support and facility delivery in rural Ghana. PLoS One. 2018;13(11):e0206429. https://doi.org/10.1371/journal.pone.0206429
21. McDonald AD, McDonald JC, Armstrong B, Cherry NM, Nolin AD, Robert D. Prematurity and work in pregnancy. Occup Environ Med. 1988;45(1):56-62.
22. McLeish J, Redshaw M. Mothers' accounts of the impact on emotional wellbeing of organised peer support in pregnancy and early parenthood: a qualitative study. BMC Pregnancy Childbirth. 2017;17(1):1-4. https://doi.org/10.1186/s12884-017-1220-0

23. Iranzad I, Bani S, Hasanpour S, Mohammadalizadeh S, Mirghafourvand M. Perceived social support and stress among pregnant women at health centers of Iran-Tabriz. J Caring Sci. 2014;3(4):287-295. https://doi.org/10.5681/jcs.2014.031
24. Ginja S, Coad J, Bailey E, Kendall S, Goodenough T, Nightingale S, et al. Associations between social support, mental wellbeing, self-efficacy and technology use in first-time antenatal women: data from the BaBBLeS cohort study. BMC Pregnancy Childbirth. 2018;18(1):1-1. https://doi.org/10.1186/s12884-018-2049-x
25. Shah MK, Gee RE, Theall KP. Partner support and impact on birth outcomes among teen pregnancies in the United States. J Pediatr Adolesc Gynecol. 2014;27(1):14-9. https://doi.org/10.1016/j.jpag.2013.08.002
26. Mabetha K, Soepnel L, Klingberg S, Mabena G, Motlhatlhedi M, Norris SA, et al. Social Support during pregnancy: A phenomenological exploration of young women's experiences of support networks on pregnancy care and wellbeing in Soweto, South Africa. medRxiv [Preprint]. 2022. [cited 2023 January 17]. Available from: https://www.medrxiv.org/content/10.1101/2022.04.03.22273162v1.full
27. Stapleton LR, Schetter CD, Westling E, Rini C, Glynn LM, Hobel CJ, et al. Perceived partner support in pregnancy predicts lower maternal and infant distress. J Fam Psychol. 2012;26(3):453-463. https://doi.org/10.1037/a0028332
28. Kroelinger CD, Oths KS. Partner support and pregnancy wantedness. Birth. 2000;27(2):112-9.
29. Martinez G, Copen CE, Abma JC. Teenagers in the United States; sexual activity, contraceptive use, and childbearing, 2006-2010 national survey of family growth. Washington, DC: National Center for Health Statistics; 2011
30. Peltzer K, Jones D, Weiss SM, Shikwane E. Promoting male involvement to improve PMTCT uptake and reduce antenatal HIV infection: a cluster randomized controlled

trial protocol. BMC Public Health. 2011;11(1):1-0. https://doi.org/10.1186/1471-2458-11-778

31. Dudas RB, Csatordai S, Devosa I, Töreki A, Andó B, Barabás K, Pál A, Kozinszky Z. Obstetric and psychosocial risk factors for depressive symptoms during pregnancy. Psychiatry Res. 2012;200(2-3):323-8. https://doi.org/10.1016/j.psychres.2012.04.017

32. Ulin PR, Robinson ET, Tolley EE. Qualitative Methods in Public Health: A Field Guide for Applied Research. San Francisco, CA: Jossey-Bass; 2005.

33. Polkinghorne DE. Language and meaning: Data collection in qualitative research. J Couns Psychol. 2005;52(2):137. https://doi.org/10.1037/0022-0167.52.2.137

34. Padfield M, Procter I. The effect of interviewer's gender on the interviewing process: A comparative enquiry. Sociology. 1996;30(2):355-66.

35. Babbie E. Research design. The practice of social research. 12th ed. Belmont, CA: Cengage Learning; 2010.

36. Rice PL, Ezzy D. Qualitative research methods: A health focus. Melbourne: Oxford University Press; 1999.

37. Braun V, Clarke V. Using thematic analysis in psychology. Qual Res Psychol. 2006;3(2):77-101.

38. Kaur S, Ng CM, Badon SE, Jalil RA, Maykanathan D, Yim HS, Mohamed HJ. Risk factors for low birth weight among rural and urban Malaysian women. BMC Public Health. 2019;19(4):1-10. https://doi.org/10.1186/s12889-019-6864-4

39. Shafaie FS, Mirghafourvand M, Rahmati M, Nouri P, Bagherinia M. Association between psychological status with perceived social support in pregnant women referring to Tabriz health centers. J Matern Fetal Neonatal Med. 2018;31(12):1554-60. https://doi.org/10.1080/14767058.2017.1319934

40. Rashid A, Mohd R. Poor social support as a risk factor for antenatal depressive symptoms among women attending public antennal clinics in Penang, Malaysia. Reprod. Health. 2017;14(1):1-8. https://doi.org/10.1186/s12978-017-0404-4
41. Ellis-Sloan K, Tamplin A. Teenage mothers and social isolation: The role of friendship as protection against relational exclusion. Soc Policy Soc. 2019 Apr;18(2):203-18. https://doi:10.1017/S1474746418000106
42. Li X, Gao R, Dai X, Liu H, Zhang J, Liu X, Si D, Deng T, Xia W. The association between symptoms of depression during pregnancy and low birth weight: a prospective study. BMC pregnancy and childbirth. 2020;20(1):1-7. https://doi.org/10.1186/s12884-020-2842-1
43. Edmonds JK, Paul M, Sibley LM. Type, content, and source of social support perceived by women during pregnancy: Evidence from Matlab, Bangladesh. J Health Popul Nutr. 2011;29(2):163-173. https://doi.org/10.3329/jhpn.v29i2.7859
44. McLeish J, Redshaw M. Mothers' accounts of the impact on emotional wellbeing of organised peer support in pregnancy and early parenthood: a qualitative study. BMC Pregnancy Childbirth. 2017;17(1):1-4. https://doi.org/10.1186/s12884-017-1220-0
45. Cohen S, Wills TA. Stress, social support, and the buffering hypothesis. Psychol Bull. 1985;98(2):310.
46. Giesbrecht GF, Poole JC, Letourneau N, Campbell T, Kaplan BJ, APrON Study Team. The buffering effect of social support on hypothalamic-pituitary-adrenal axis function during pregnancy. Psychosom Med. 2013;75(9):856-62. https://doi:10.1097/PSY.0000000000000004
47. Basharpoor S, Heydarirad H, Daryadel SJ, Heydari F, Ghamari Givi H, Kishore J. The role of perceived stress and social support among predicting anxiety in pregnant

women. J Holist Nurs Midwifery. 2017;27(2):9-16. https://doi:10.18869/acadpub.hnmj.27.2.9

48. Moyer CA, Adongo PB, Aborigo RA, Hodgson A, Engmann CM. 'They treat you like you are not a human being': maltreatment during labour and delivery in rural northern Ghana. Midwifery. 2014;30(2):262-8. https://doi.org/10.1016/j.midw.2013.05.006

49. Singh D, Newburn M, Smith N, Wiggins M. The information needs of first-time pregnant mothers. Br. J. Midwifery. 2002;10(1):54-8. https://doi.org/10.12968/bjom.2002.10.1.10054

50. Kululanga LI, Kadango A, Lungu G, Jere D, Ngwale M, Kumbani LC. Knowledge deficit on health promotion activities during pregnancy: the case for adolescent pregnant women at Chiladzulu District, Malawi. BMC Pregnancy Childbirth. 2020;20(1):1-9. https://doi.org/10.1186/s12884-020-03386-w

51. Bjelica A, Cetkovic N, Trninic-Pjevic A, Mladenovic-Segedi L. The phenomenon of pregnancy-A psychological view. Ginekol Polska. 2018;89(2):102-6. https://doi.10.5603/GP.a2018.0017

52. Bonillas CA, Feehan R. Normalizing the changes experienced during each trimester of pregnancy. J Perinat Educ. 2008;17(1):39-43. https://doi.org/10.1624/105812408X266287

53. Reddy KJ, Menon KR, Thattil A. Academic stress and its sources among university students. Biomed Pharmacol J. 2018;11(1):531-7. https://dx.doi.org/10.13005/bpj/1404

54. Kumaraswamy N. Academic stress, anxiety and depression among college students: A brief review. International review of social sciences and humanities. 2013;5(1):135-43.

55. Claxton A, Perry- Jenkins M. No fun anymore: Leisure and marital quality across the transition to parenthood. J Marriage Fam. 2008;70(1):28-43.

56. Tokhi M, Comrie-Thomson L, Davis J, Portela A, Chersich M, Luchters S. Involving men to improve maternal and newborn health: a systematic review of the effectiveness of interventions. PLoS One. 2018;13(1):e0191620. https://doi.org/10.1371/journal.pone.0191620

57. Yargawa J, Leonardi-Bee J. Male involvement and maternal health outcomes: systematic review and meta-analysis. J Epidemiol Community Health. 2015;69(6):604-12. http://dx.doi.org/10.1136/jech2014-204784

58. Nathane-Taulela M, Nduna M. Young women's experiences following discovering a biological father in Mpumalanga province, South Africa. Open Fam Stud J. 2014;6(1):62-68.

59. Kazemi A, Ghaedrahmati M, Kheirabadi G. Partner's emotional reaction to pregnancy mediates the relationship between pregnancy planning and prenatal mental health. BMC Pregnancy Childbirth. 2021;21(1):1-8. https://doi.org/10.1186/s12884-021-03644-5

60. Govender D, Taylor M, Naidoo S. Adolescent pregnancy and parenting: Perceptions of healthcare providers. J Multidiscip Healthc. 2020;13:1607-1628. https://doi.org/10.2147/JMDH.S258576

61. Gibore NS, Bali TA. Community perspectives: An exploration of potential barriers to men's involvement in maternity care in a central Tanzanian community. PLoS One. 2020;15(5):e0232939. https://doi.org/10.1371/journal.pone.0232939

62. Forbes F, Wynter K, Wade C, Zeleke BM, Fisher J. Male partner attendance at antenatal care and adherence to antenatal care guidelines: secondary analysis of 2011

Ethiopian demographic and health survey data. BMC Pregnancy Childbirth. 2018;18(1):145. https://doi.org/10.1186/s12884-018-1775-4

63. World Health Organization. A human rights-based approach to health, 2015. https://www.who.int/hhr/news/hrba_to_health2.pdf. Accessed 25 Jul 2021.

Re: Experiences of social support among unmarried pregnant university students in South Africa. PONE-D-22-16990

Dear Editor,

A sincere thank you for your insightful comments on our manuscript. The comments will definitely help improve the manuscript. We have revised the paper in response to the reviewers' comments.

The next section below contains our responses to the reviewers' comments.

Reviewer #1: Research ethics: It is indeed important to observe research ethics but this does not justify your statement that data cannot be shared publicly because of privacy and confidentiality issues. You can anonymize the transcripts and make them publicly available. Already you have used pseudonyms to protect the identity of participants (Line 210 and Table 1).

We have provided the minimal underlying data as a supporting (supplementary) information file.

You have correctly indicated (Line 166) that 'issues relating to pregnancy among young people are sensitive' but you have revealed the identity of the institution where data was collected (Lines 160, 161 & 163). I suggest that the name of the university be concealed.

Thank you for this suggestion. We have not revealed the name of the University where the study was conducted in the manuscript.

Participants: The study is among unmarried pregnant university students as you clearly indicated in the title (Line 1) and some other places in the manuscript. I find it confusing when you refer to participants as 'pregnant women'; 'pregnant young women'; 'unmarried pregnant students'; young unmarried women' in Lines 46, 147, 321, 555 and at some other places. I suggest that a concept to refer to participants be identified and be used consistently. Looking at the age of the participants (Table 2), is it appropriate to refer to all of them as young women?

Thank you for this comment. We have chosen to refer to participants as "unmarried pregnant students" throughout the manuscript.

I suggest that Table 1 be moved to Results as it reflects what the study found.

We have moved Table 1 to the Findings section as suggested.

It is a good practice to describe the qualifications and experiences of researchers in a qualitative research. The description (Lines 174 - 179) of the first author (TMP) is correct, and I suggest that the other authors be described as well.

We have added a brief description of the qualifications and experiences of all the researchers who collaborated in the development of the manuscript. For example: "OA is a Public Health Promotion and Research Methods specialist" and "PN; a post-doctoral fellow and researcher".

Language: Replace Master's (Lines 175 - 177) with Masters.

We have made the correction and replaced Master's with Masters.

School (Lines 242, 482) should be replaced with university.

Thank you for this correction. We have used the term "university" in line 245 and "tuition" in line 485.

The manner of writing ANC (Lines 527, 536 and 551) should rectified. Write in full with abbreviation in brackets on first time use, then abbreviation thereafter.

We have rectified this error and also rectified in the different sections of the manuscript.

The concept 'clinics' (Lines 536 - 538) needs to be clarified as specialists run clinics in both private and government/public health services.

Thank you for this suggestion. We have clarified the concept "clinics" and explained that, "Some participants explained that they were not attending ANC in public clinics but instead were consulting specialists like gynaecologists and obstetricians in private hospitals to monitor their pregnancy. This was mostly because they felt that they needed to be checked by specialists who were not readily available in public clinics".

Lines 551 - 557 regarding ANC need to be rephrased as ANC is available through private and public health facilities.

We have revised this section and removed some material as it was not relevant. This section now reads: “Overall, it was established that most unmarried pregnant students seek ANC in public clinics and hospitals alone without the support of their male partners. The participants generally considered ANC to be unfriendly. In addition, partner attendance was not specifically encouraged or recommended by healthcare workers”.

Furthermore, pregnant women can deliver in a public hospital even when they did not attend ANC at a public/government facility or even when they did not attend ANC at all. Therefore, Lines 668 - 669 should be revised.

Thank you for this correction. We have amended the section and explained that, “The South Africa Department of Health guidelines recommend that pregnant women seek ANC as soon as they suspect a pregnancy and encourages all pregnant women to attend at least eight ANC visits”.

Line 553 - 554 contradicts itself regarding affordability of private healthcare (none & except), see also Lines 670 - 671 regarding ability to pay for private healthcare.

We have removed this contradicting sentence.

Pregnancy and conception (Line 639) should be conception and pregnancy.

Thank you for this correction, we have amended to highlight the suggested change.

Description of study design (Lines 150 - 158) should be rephrased to clarify if qualitative research is a design or a method. Based on what you wrote in Line 157, the appropriate design appears to be a narrative design or even a phenomenological design might be suitable.

We have rephrased in the manuscript and included “descriptive” qualitative research design. There are a number of researchers who believe and support the fact that ‘descriptive qualitative research design’ is a viable and acceptable research design (Doyle et al., 2020; Lambert & Lambert, 2012; Ulin et al., 2005). This design is an acceptable research design for robust scholarly research and has received varying degrees of acceptance within the academic community. In this study we use the descriptive qualitative research as a design in its own right. The most frequently proposed rationale for the use of a descriptive design is to provide

straightforward descriptions of experiences and perceptions (Sandelowski, 2000), particularly in areas where little is known about the topic under study.

Doyle, L., McCabe, C., Keogh, B., Brady, A., & McCann, M. (2020). An overview of the qualitative descriptive design within nursing research. Journal of Research in Nursing, 25(5), 443-455.

Lambert, V. A., & Lambert, C. E. (2012). Qualitative descriptive research: An acceptable design. Pacific Rim International Journal of Nursing Research, 16(4), 255-256.

Ulin, PR, Robinson, ET, & Tolley, EE (2005). Qualitative methods in public health: A field guide for applied research. Jossey-Bass.

Sandelowski M. Whatever happened to qualitative description?. Research in nursing & health. 2000;23(4):334-40.

Indicate under Data collection if field notes were collected or not as they are important to add to the context of the interviews.

We have explained that, "The interviewer (TMP) made field notes during the interview and then summarized these following the interview. The field notes were thereafter used to develop analytical memos.".

Data analysis: Indicate (Line 209) that data was transcribed verbatim. This is only indicated in the Abstract (Line 39), but it is important to indicate this in the main document.

We have highlighted in the manuscript that data was transcribed verbatim. The sentence now reads, "The audio-recorded data was transcribed verbatim by the first author (TMP)".

Results: Indicate the number of sub-themes in Line 229.

We have indicated the number of sub-themes. "The findings are organized into four main themes and ten sub-themes identified from the analysis of the data".

Line 239 should say sub-themes, not themes. Clarify also 'four main subthemes arose

We have amended to read, "The following three sub-themes emerged from the interviews.".

(Line 334). Is the word 'main' necessary?

We have removed the word.

The following concepts [many of the participants; several participants; most participants; a few of the participants] are not appropriate to report qualitative findings as they suggest numbers; appropriate concepts are some participates, other participants and others. Writing just 'participants' may also imply that it is all participants who shared that experience. I found both appropriate and inappropriate concepts in this manuscript and suggest that the inappropriate concepts be replaced.

Thank you for this comment. We have used the suggested qualifiers in some places where possible, but in some instances, we felt that some of the qualifiers we had used were the most appropriate way to convey the general experiences of the participants in relation to a particular phenomenon.

Line 311 should acknowledge that some participants had previous pregnancies and therefore knew what to expect.

We have amended this section to read, “One of the participants who had a previous pregnancy explained the difficulties of having a first pregnancy without adequate knowledge of the accompanying physical changes”.

Lines 319 - 321 and 330 - 331 appears to be misplaced; they would suit the Discussion section.

We have removed the sentence in line 319-321. In line 330-331 we have amended the sentence to show our interpretation of the participant’s experience. The sentence now reads, “The participant’s narrative highlights the challenges of poor nutritional intake during pregnancy”.

Context: It is important to mention if these pregnancies were planned or not, if participants disclosed their pregnancies to whoever they expected to give social support and whether they are still in a continuing and healthy relationships with the partner who impregnated them. Where (on-campus or off-campus) and with whom were participants staying? Lines 599, 606-607 are not clear regarding where participants were staying.

In the design and data collection of the study we did not specifically seek to explore information on the nature of the pregnancies (planned or unplanned); whether they were staying on-campus or off-campus; and with whom they were staying. However, some of this information emerged during the course of our interviews and hence it is reflected in some of the narratives. In instances where this information emerges we attempt to provide some context. Pregnancies were disclosed in all cases to significant others who were expected to offer social support and we have highlighted this in the manuscript.

Discussion: Lines 590 and 648-649 give an impression that types of social support were rated in this study. Rating is a quantitative concept.

The study sought to explore the social support needs of unmarried pregnant students in tertiary institutions during pregnancy and the puerperal period, and provide insight into the types of social support that were most important to them (primary source of support). However, we have revised the language such that it does not reflect quantitative concepts:

“Although a previous study reported on the importance of emotional support during pregnancy, it was not rated regarded as the most important type of support needed”.

“Partners’ instrumental and financial supports were also considered to be important sources of support”.

Lines 653-656 should say some African cultures because in some communities in South Africa, paying damages means admission that one has impregnated a woman by paying a fine and not a commitment to support the child financially (paying damages is a once-off payment).

Thank you for this correction. We have specified that: “In some African cultures, payment of damages is perceived as a form of acceptance of paternity. This process was perceived by the participants to give a form of dignity to her family and some assurance that the father was prepared to take care of the child”.

Recommendation: It is not clear on which findings are the recommendations on behaviour change programme (Line 688) and rights-based approach (Line 639) based.

In the manuscript we explain that the participants mentioned that some male partners were not usually willing to help them with tasks. Experiences of social stigma were also reported at public health facilities. Hence, to overcome some of these challenges’ interventions informed

by behaviour change and rights-based approach are essential. We explain in the manuscript that:

"Absence or lack of emotional social support from significant others, particularly partners may exacerbate the stressors of pregnancy among students. Tertiary institutions should partner with established social initiatives offering services for men in the country to engage and mentor young men one-on-one and in groups about the importance of the father's role in the child's development and how to best support their partners to improve pregnancy outcomes, irrespective of relationship status. Culturally sensitive health promotion programmes targeted at 'father inclusive' practices and behaviour change communication programmes to stimulate social support may have positive changes in terms of the supportive role of partners".

"Male involvement programmes can be integrated in ANC whereby services are restructured to accommodate male partners to ensure a sense of involvement and responsibility. However, for these programmes to work, the ANC environment should be user friendly for young women and their partners. A rights-based approach must be consistently adopted when delivering maternal healthcare. Training and on-going education should be prioritised for healthcare workers regarding the provision of positive, respectful and supportive non-judgmental antenatal and postnatal care to their clients irrespective of their age or marital status".

Who are the 'antenatal health personnel' in Line 695?

We have amended the sentence and explained that: "Training and on-going education should be prioritised for healthcare workers regarding the provision of positive, respectful and supportive non-judgmental antenatal and postnatal care to their clients irrespective of their age or marital status."

Reviewer #2: There is need to rephrase the topic to experiences of unmarried pregnant students on male partner social support to speak to the background of the study.

Thank you for this comment. We have revised the background to highlight that the main issue of concern is social support needs for unmarried pregnant students and not only male partner social support. While we acknowledge that male social support is one of the main themes that emerged in the findings, the focus of the study is social support in general. However, we have rephrased the topic to, "Perspectives about social support among unmarried pregnant university students in South Africa".

On abstract the section of methods line 35, the candidates say perceptions there must be consistency in usage of words, what is explored are experiences.

We have rephrased to clearly highlight that the study is exploring perspectives about social support among unmarried pregnant students in a university setting and not experiences.

40-41 the results should be reported in line with the study aim which is pregnant women support needs and when it was needed the most.

We have revised this section to highlight the aim of the study which is, "to explore the perspectives of social support among unmarried students in a university setting during pregnancy and the puerperium; and the period when most support is needed". The results are reported in line with this study aim.

Under line 150, it should be clear which research design under qualitative research approach was employed that informed the thematic content analysis.

We have rephrased in the manuscript and included "descriptive" qualitative research design. There are a number of researchers who believe and support the fact that 'descriptive qualitative research design' is a viable and acceptable research design (Doyle et al., 2020; Lambert & Lambert, 2012; Ulin et al., 2005). This design is an acceptable research design for robust scholarly research and has received varying degrees of acceptance within the academic community. In this study we use the descriptive qualitative research as a design in its own right. The most frequently proposed rationale for the use of a descriptive design is to provide straightforward descriptions of experiences and perceptions (Sandelowski, 2000), particularly in areas where little is known about the topic under study. In this type of design, data analysis "does not use a pre-existing set of rules that have been generated from the philosophical or epistemological stance of the discipline that created the specific qualitative research approach. Rather, qualitative descriptive research is purely data-derived in that codes are generated from the data in the course of the study" (Lambert & Lambert, 2012, p.256). Thus,

we have further explained in the manuscript that. “As qualitative descriptive research is purely data-derived, we specifically employed an inductive approach to thematic analysis”.

Under 171 how many students were snowballed were in puerperal period.

Among the first group of students who were approached, all of them were pregnant. None of them were in the puerperal period. However, through snowball sampling we were able to identify these students who had recently given birth. Essentially, all of the participants in the puerperal period were recruited through snowball. In the manuscript we explain that: “The first author (TMP) initially approached three pregnant young students who stayed in the same residence as her. She (TMP) discussed about the study with those young pregnant students and requested them to identify and recommend other students who were pregnant or had recently given birth”.

Line 145 should specify the experiences from unmarried pregnant students are about "male social support".

We have explained earlier on that the study was not specifically focusing on “male partner support” but rather support needs of unmarried pregnant students. We have explained in the manuscript that, “Therefore, the aim of this study is to explore the perspectives about social support among unmarried pregnant students in a university setting”.

119-144 provides an impression that the social support explored is only from male partners. Line 147 emphasis the said point which contradicts the study topic.

We have revised this section and deleted some material which suggested that the social support explored is only from male partners.

On line 197 the interview indicates that the schedule elicited the support needs of pregnant students and the period when most support is needed. The results should be aligned to what the interview guide elicited

We have revised this section to include all the topics covered in the interview schedule. This section now reads, “The questions contained in the interview schedule included the socio-demographic variables of participants and their partners, the support needs of pregnant

students, the period when most support is needed and types of male partner support needed".

on line 149, the section on study objectives should be included.

We have included the objectives of the study:

1) To explore the social support needs of unmarried students in tertiary institutions during pregnancy and the puerperium.

2) To explore the period when most support is needed by unmarried students in tertiary institutions during pregnancy and the puerperium.

line 171-173, how many peripheral students were identified, only pregnant student are mentioned.

Among the first group of students who were approached none of them were in puerperal period and we only managed to gather these participants in the puerperal period through referral. We have explained that: "The first author (TMP) initially approached three pregnant young students who stayed in the same residence as her. She (TMP) discussed about the study with those young pregnant students and requested them to identify and recommend other students who were pregnant or had recently given birth".

Under 189, what ethical codes were considered in this study and the study is sensitive in nature, how was the emotional risk addressed.

We explained that: "Data collection was carried out in accordance with relevant guidelines and the participants could withdraw from the study at any point. Although the study was of a sensitive nature, we did not anticipate any negative effects on the participants. However, we had arranged counselling services for the participants if the need arises but none of the participants requested for these services".

Line 205, What was used to record data and where was data stored.

We have explained that: "We used a digital audio voice recorder and the recordings were safely stored in a password protected computer".

Under 226, I suggest authors remove the social support needs from the general social network. That is, the first section of social support needs can be deleted, and the results presents the social support needs from participants male partners and the period they needed the support in order to align with the strong assertions made in the background.

Thank you for this suggestion. We have revised the background to highlight that the study will explore social support needs of unmarried pregnant students and not primarily focus on the social support needs from participants male partners. We believe that removing the social support needs from the general social network will not do justice to the study as these are some of the main issues that were expressed by the participants.

558 most participants are undergraduates and what implications does this have to the present study. The socio-economic background of participants is something that could have been explored from the participants

We did not specifically seek to explore the influence of the socio-economic background of participants nor did we aim to understand the implications of the level of study on the experiences of the participants. We acknowledge that these aspects could have provided a different perspective and insight to the study's findings. However, there are sections where we elaborate on the influence of the participants' socio-economic background. For example, "*The baby needs so many things so as to grow in a good state; nappies, warm clothes, milk, formula [baby food] since am not going to breastfeed so financially it will be much required from him. [She gives an example]-cause my cousin gave birth in April and the father of the baby hasn't given her a single cent since she gave birth, since she fell pregnant, she hasn't received any money from the baby's daddy and telling me things are difficult, so financially it is so important*"(pg. 20, line 438-443); "Lastly, financial support was needed to buy food, to cover transport costs and medical expenses and to prepare for the arrival of the unborn baby" (pg. 28, ;line 611-613).

What recommendations can be made to deal with students' emotional risk that the lack of male partner support impose on unmarried pregnant students. Additionally, what strategies can be recommended to cultivate a culture of male social support towards their female pregnant partners.

Thank you for highlighting these issues. As part of the recommendations we have added that, "In instances where emotional support from their male partners is not feasible, unmarried

pregnant students need to identify alternate sources of support such as counselling support, friends, church and social media”.

“Tertiary institutions should partner with established social initiatives offering services for men in the country to engage and mentor young men one-on-one and in groups about the importance of the father’s role in the child’s development and how to best support their partners to improve pregnancy outcomes, irrespective of relationship status. Culturally sensitive health promotion programmes targeted at ‘father inclusive’ practices and behaviour change communication programmes to stimulate social support may have positive changes in terms of the supportive role of partners. Implementors of such programmes should consider adopting innovative outreach strategies that appeal to young men’s interests and hang-out spots, such as bars and sports arenas**”.**
