# Supplementary material for: Perspectives about social support among unmarried pregnant university students in South Africa
Source: PLoS One. 2023 Apr 24;18(4):e0284906. doi: 10.1371/journal.pone.0284906 (PMC10124874; doi:10.1371/journal.pone.0284906)
Supplement: Attachment — Submitted filename: Response to reviewers.docx [file pone.0284906.s002.docx]

**Re: Experiences of social support among unmarried pregnant university students in South Africa.** **PONE-D-22-16990**

Dear Editor,

A sincere thank you for your insightful comments on our manuscript. The comments will definitely help improve the manuscript. We have revised the paper in response to the reviewers’ comments.

The next section below contains our responses to the reviewers’ comments.

**Reviewer #1: Research ethics: It is indeed important to observe research ethics but this does not justify your statement that data cannot be shared publicly because of privacy and confidentiality issues. You can anonymize the transcripts and make them publicly available. Already you have used pseudonyms to protect the identity of participants (Line 210 and Table 1).**

We have provided the minimal underlying data as a supporting (supplementary) information file.

**You have correctly indicated (Line 166) that 'issues relating to pregnancy among young people are sensitive' but you have revealed the identity of the institution where data was collected (Lines 160, 161 & 163). I suggest that the name of the university be concealed.**

Thank you for this suggestion. We have not revealed the name of the University where the study was conducted in the manuscript.  **Participants: The study is among unmarried pregnant university students as you clearly indicated in the title (Line 1) and some other places in the manuscript. I find it confusing when you refer to participants as 'pregnant women'; 'pregnant young women'; 'unmarried pregnant students'; young unmarried women' in Lines 46, 147, 321, 555 and at some other places. I suggest that a concept to refer to participants be identified and be used consistently. Looking at the age of the participants (Table 2), is it appropriate to refer to all of them as young women?**

Thank you for this comment. We have chosen to refer to participants as “unmarried pregnant students” throughout the manuscript.

**I suggest that Table 1 be moved to Results as it reflects what the study found.**

We have moved Table 1 to the Findings section as suggested.

**It is a good practice to describe the qualifications and experiences of researchers in a qualitative research. The description (Lines 174 - 179) of the first author (TMP) is correct, and I suggest that the other authors be described as well.**

We have added a brief description of the qualifications and experiences of all the researchers who collaborated in the development of the manuscript. For example: “OA is a Public Health Promotion and Research Methods specialist” and “PN; a post-doctoral fellow and researcher”.  **Language: Replace Master's (Lines 175 - 177) with Masters.**

We have made the correction and replaced Master's with Masters.

**School (Lines 242, 482) should be replaced with university.**

Thank you for this correction. We have used the term “university” in line 245 and “tuition” in line 485.

**The manner of writing ANC (Lines 527, 536 and 551) should rectified. Write in full with abbreviation in brackets on first time use, then abbreviation thereafter.**

We have rectified this error and also rectified in the different sections of the manuscript.

**The concept 'clinics' (Lines 536 - 538) needs to be clarified as specialists run clinics in both private and government/public health services.**

Thank you for this suggestion. We have clarified the concept “clinics” and explained that, “Some participants explained that they were not attending ANC in public clinics but instead were consulting specialists like gynaecologists and obstetricians in private hospitals to monitor their pregnancy. This was mostly because they felt that they needed to be checked by specialists who were not readily available in public clinics”.

**Lines 551 - 557 regarding ANC need to be rephrased as ANC is available through private and public health facilities.**

We have revised this section and removed some material as it was not relevant. This section now reads: “Overall, it was established that most unmarried pregnant students seek ANC in public clinics and hospitals alone without the support of their male partners. The participants generally considered ANC to be unfriendly. In addition, partner attendance was not specifically encouraged or recommended by healthcare workers”.

**Furthermore, pregnant women can deliver in a public hospital even when they did not attend ANC at a public/government facility or even when they did not attend ANC at all. Therefore, Lines 668 - 669 should be revised.**

Thank you for this correction. We have amended the section and explained that, “The South Africa Department of Health guidelines recommend that pregnant women seek ANC as soon as they suspect a pregnancy and encourages all pregnant women to attend at least eight ANC visits”.

**Line 553 - 554 contradicts itself regarding affordability of private healthcare (none & except), see also Lines 670 - 671 regarding ability to pay for private healthcare.**

We have removed this contradicting sentence.

**Pregnancy and conception (Line 639) should be conception and pregnancy.**

Thank you for this correction, we have amended to highlight the suggested change.

**Description of study design (Lines 150 - 158) should be rephrased to clarify if qualitative research is a design or a method. Based on what you wrote in Line 157, the appropriate design appears to be a narrative design or even a phenomenological design might be suitable.**

We have rephrased in the manuscript and included “descriptive” qualitative research design. There are a number of researchers who believe and support the fact that ‘descriptive qualitative research design’ is a viable and acceptable research design (Doyle et al., 2020; Lambert & Lambert, 2012; Ulin et al., 2005). This design is an acceptable research design for robust scholarly research and has received varying degrees of acceptance within the academic community. In this study we use the descriptive qualitative research as a design in its own right. The most frequently proposed rationale for the use of a descriptive design is to provide straightforward descriptions of experiences and perceptions (Sandelowski, 2000), particularly in areas where little is known about the topic under study.

Doyle, L., McCabe, C., Keogh, B., Brady, A., & McCann, M. (2020). An overview of the qualitative descriptive design within nursing research. Journal of Research in Nursing, 25(5), 443-455.

Lambert, V. A., & Lambert, C. E. (2012). Qualitative descriptive research: An acceptable design. Pacific Rim International Journal of Nursing Research, 16(4), 255-256.

Ulin, PR, Robinson, ET, & Tolley, EE (2005). Qualitative methods in public health: A field guide for applied research. Jossey-Bass.

Sandelowski M. Whatever happened to qualitative description?. Research in nursing & health. 2000;23(4):334-40.

**Indicate under Data collection if field notes were collected or not as they are important to add to the context of the interviews.**

We have explained that, “The interviewer (TMP) made field notes during the interview and then summarized these following the interview. The field notes were thereafter used to develop analytical memos.”.

**Data analysis: Indicate (Line 209) that data was transcribed verbatim. This is only indicated in the Abstract (Line 39), but it is important to indicate this in the main document.**

We have highlighted in the manuscript that data was transcribed verbatim. The sentence now reads, “The audio-recorded data was transcribed verbatim by the first author (TMP)”. **Results: Indicate the number of sub-themes in Line 229.**

We have indicated the number of sub-themes. “The findings are organized into four main themes and ten sub-themes identified from the analysis of the data”.

**Line 239 should say sub-themes, not themes. Clarify also 'four main subthemes arose**

We have amended to read, “The following three sub-themes emerged from the interviews.”.

**(Line 334). Is the word 'main' necessary?**

We have removed the word.

**The following concepts [many of the participants; several participants; most participants; a few of the participants] are not appropriate to report qualitative findings as they suggest numbers; appropriate concepts are some participates, other participants and others. Writing just 'participants' may also imply that it is all participants who shared that experience. I found both appropriate and inappropriate concepts in this manuscript and suggest that the inappropriate concepts be replaced.**

Thank you for this comment. We have used the suggested qualifiers in some places where possible, but in some instances, we felt that some of the qualifiers we had used were the most appropriate way to convey the general experiences of the participants in relation to a particular phenomenon.

**Line 311** **should acknowledge that some participants had previous pregnancies and therefore knew what to expect.**

We have amended this section to read, “One of the participants who had a previous pregnancy explained the difficulties of having a first pregnancy without adequate knowledge of the accompanying physical changes”.

**Lines 319 - 321 and 330 - 331 appears to be misplaced; they would suit the Discussion section.**

We have removed the sentence in line 319-321. In line 330-331 we have amended the sentence to show our interpretation of the participant’s experience. The sentence now reads, “The participant’s narrative highlights the challenges of poor nutritional intake during pregnancy”.

**Context: It is important to mention if these pregnancies were planned or not, if participants disclosed their pregnancies to whoever they expected to give social support and whether they are still in a continuing and healthy relationships with the partner who impregnated them. Where (on-campus or off-campus) and with whom were participants staying? Lines 599, 606-607 are not clear regarding where participants were staying.**

In the design and data collection of the study we did not specifically seek to explore information on the nature of the pregnancies (planned or unplanned); whether they were staying on-campus or off-campus; and with whom they were staying. However, some of this information emerged during the course of our interviews and hence it is reflected in some of the narratives. In instances where this information emerges we attempt to provide some context. Pregnancies were disclosed in all cases to significant others who were expected to offer social support and we have highlighted this in the manuscript. **Discussion: Lines 590 and 648-649 give an impression that types of social support were rated in this study. Rating is a quantitative concept.**

The study sought to explore the social support needs of unmarried pregnant students in tertiary institutions during pregnancy and the puerperal period, and provide insight into the types of social support that were most important to them (primary source of support). However, we have revised the language such that it does not reflect quantitative concepts:

“Although a previous study reported on the importance of emotional support during pregnancy, it was not rated regarded as the most important type of support needed”.

“Partners’ instrumental and financial supports were also considered to be important sources of support”.

**Lines 653-656 should say some African cultures because in some communities in South Africa, paying damages means admission that one has impregnated a woman by paying a fine and not a commitment to support the child financially (paying damages is a once-off payment).**

Thank you for this correction. We have specified that: “In some African cultures, payment of damages is perceived as a form of acceptance of paternity. This process was perceived by the participants to give a form of dignity to her family and some assurance that the father was prepared to take care of the child”.

**Recommendation: It is not clear on which findings are the recommendations on behaviour change programme (Line 688) and rights-based approach (Line 639) based.**

In the manuscript we explain that the participants mentioned that some male partners were not usually willing to help them with tasks. Experiences of social stigma were also reported at public health facilities. Hence, to overcome some of these challenges’ interventions informed by behaviour change and rights-based approach are essential. We explain in the manuscript that:

“Absence or lack of emotional social support from significant others, particularly partners may exacerbate the stressors of pregnancy among students. Tertiary institutions should partner with established social initiatives offering services for men in the country to engage and mentor young men one-on-one and in groups about the importance of the father’s role in the child’s development and how to best support their partners to improve pregnancy outcomes, irrespective of relationship status. Culturally sensitive health promotion programmes targeted at ‘father inclusive’ practices and behaviour change communication programmes to stimulate social support may have positive changes in terms of the supportive role of partners”.

“Male involvement programmes can be integrated in ANC whereby services are restructured to accommodate male partners to ensure a sense of involvement and responsibility. However, for these programmes to work, the ANC environment should be user friendly for young women and their partners. A rights-based approach must be consistently adopted when delivering maternal healthcare. Training and on-going education should be prioritised for healthcare workers regarding the provision of positive, respectful and supportive non-judgmental antenatal and postnatal care to their clients irrespective of their age or marital status”.

**Who are the 'antenatal health personnel' in Line 695?**

We have amended the sentence and explained that: “Training and on-going education should be prioritised for healthcare workers regarding the provision of positive, respectful and supportive non-judgmental antenatal and postnatal care to their clients irrespective of their age or marital status.”

**Reviewer #2: There is need to rephrase the topic to experiences of unmarried pregnant students on male partner social support to speak to the background of the study.**

Thank you for this comment. We have revised the background to highlight that the main issue of concern is social support needs for unmarried pregnant students and not only male partner social support. While we acknowledge that male social support is one of the main themes that emerged in the findings, the focus of the study is social support in general. However, we have rephrased the topic to, “Perspectives about social support among unmarried pregnant university students in South Africa”.  **On abstract the section of methods line 35, the candidates say perceptions there must be consistency in usage of words, what is explored are experiences.**

We have rephrased to clearly highlight that the study is exploring perspectives about social support among unmarried pregnant students in a university setting and not experiences.  **40-41 the results should be reported in line with the study aim which is pregnant women support needs and when it was needed the most.**

We have revised this section to highlight the aim of the study which is, “to explore the perspectives of social support among unmarried students in a university setting during pregnancy and the puerperium; and the period when most support is needed”. The results are reported in line with this study aim. **Under line 150, it should be clear which research design under qualitative research approach was employed that informed the thematic content analysis.**

We have rephrased in the manuscript and included “descriptive” qualitative research design. There are a number of researchers who believe and support the fact that ‘descriptive qualitative research design’ is a viable and acceptable research design (Doyle et al., 2020; Lambert & Lambert, 2012; Ulin et al., 2005). This design is an acceptable research design for robust scholarly research and has received varying degrees of acceptance within the academic community. In this study we use the descriptive qualitative research as a design in its own right. The most frequently proposed rationale for the use of a descriptive design is to provide straightforward descriptions of experiences and perceptions (Sandelowski, 2000), particularly in areas where little is known about the topic under study. In this type of design, data analysis “does not use a pre-existing set of rules that have been generated from the philosophical or epistemological stance of the discipline that created the specific qualitative research approach. Rather, qualitative descriptive research is purely data-derived in that codes are generated from the data in the course of the study” (Lambert & Lambert, 2012, p.256). Thus, we have further explained in the manuscript that. “As qualitative descriptive research is purely data-derived, we specifically employed an inductive approach to thematic analysis”.

**Under 171 how many students were snowballed were in puerperal period.**

Among the first group of students who were approached, all of them were pregnant. None of them were in the puerperal period. However, through snowball sampling we were able to identify these students who had recently given birth. Essentially, all of the participants in the puerperal period were recruited through snowball. In the manuscript we explain that: “The first author (TMP) initially approached three pregnant young students who stayed in the same residence as her. She (TMP) discussed about the study with those young pregnant students and requested them to identify and recommend other students who were pregnant or had recently given birth”. **Line 145 should specify the experiences from unmarried pregnant students are about "male social support".**

We have explained earlier on that the study was not specifically focusing on “male partner support” but rather support needs of unmarried pregnant students. We have explained in the manuscript that, “Therefore, the aim of this study is to explore the perspectives about social support among unmarried pregnant students in a university setting”.  **119-144 provides an impression that the social support explored is only from male partners. Line 147 emphasis the said point which contradicts the study topic.**

We have revised this section and deleted some material which suggested that the social support explored is only from male partners.  **On line 197 the interview indicates that the schedule elicited the support needs of pregnant students and** **the period when most support is needed. The results should be aligned to what the interview guide elicited**
We have revised this section to include all the topics covered in the interview schedule. This section now reads, “The questions contained in the interview schedule included the socio-demographic variables of participants and their partners, the support needs of pregnant students, the period when most support is needed and types of male partner support needed”.  **on line 149, the section on study objectives should be included.**

We have included the objectives of the study:

1) To explore the social support needs of unmarried students in tertiary institutions during pregnancy and the puerperium.

2) To explore the period when most support is needed by unmarried students in tertiary institutions during pregnancy and the puerperium.

**line 171-173, how many peripheral students were identified, only pregnant student are mentioned.**

Among the first group of students who were approached none of them were in puerperal period and we only managed to gather these participants in the puerperal period through referral. We have explained that: “The first author (TMP) initially approached three pregnant young students who stayed in the same residence as her. She (TMP) discussed about the study with those young pregnant students and requested them to identify and recommend other students who were pregnant or had recently given birth”.

**Under 189, what ethical codes were considered in this study and the study is sensitive in nature,** **how was the emotional risk addressed.**

We explained that: “Data collection was carried out in accordance with relevant guidelines and the participants could withdraw from the study at any point. Although the study was of a sensitive nature, we did not anticipate any negative effects on the participants. However, we had arranged counselling services for the participants if the need arises but none of the participants requested for these services”. **Line 205, What was used to record data and where was data stored.**

**We have explained that:** “We used a digital audio voice recorder and the recordings were safely stored in a password protected computer”.

**Under 226, I suggest authors remove the social support needs from the general social network. That is, the first section of social support needs can be deleted, and the results presents the social support needs from participants male partners and the period they needed the support in order to align with the strong assertions made in the background.**

Thank you for this suggestion. We have revised the background to highlight that the study will explore social support needs of unmarried pregnant students and not primarily focus on the social support needs from participants male partners. We believe that removing the social support needs from the general social network will not do justice to the study as these are some of the main issues that were expressed by the participants.

**558 most participants are undergraduates and what implications does this have to the present study. The socio-economic background of participants is something that could have been explored from the participants**

We did not specifically seek to explore the influence of the socio-economic background of participants nor did we aim to understand the implications of the level of study on the experiences of the participants. We acknowledge that these aspects could have provided a different perspective and insight to the study’s findings. However, there are sections where we elaborate on the influence of the participants’ socio-economic background. For example, “*The baby needs so many things so as to grow in a good state; nappies, warm clothes, milk, formula [baby food] since am not going to breastfeed so financially it will be much required from him. [She gives an example]-cause my cousin gave birth in April and the father of the baby hasn’t given her a single cent since she gave birth, since she fell pregnant, she hasn’t received any money from the baby’s daddy and telling me things are difficult, so financially it is so important*”(pg. 20, line 438-443); “Lastly, financial support was needed to buy food, to cover transport costs and medical expenses and to prepare for the arrival of the unborn baby” (pg. 28, ;line 611-613).

**What recommendations can be made to deal with students’ emotional risk that the lack of male partner support impose on unmarried pregnant students. Additionally, what strategies can be recommended to cultivate a culture of male social support towards their female pregnant partners.**

Thank you for highlighting these issues. As part of the recommendations we have added that, “In instances where emotional support from their male partners is not feasible, unmarried pregnant students need to identify alternate sources of support such as counselling support, friends, church and social media”.

**“**Tertiary institutions should partner with established social initiatives offering services for men in the country to engage and mentor young men one-on-one and in groups about the importance of the father’s role in the child’s development and how to best support their partners to improve pregnancy outcomes, irrespective of relationship status. Culturally sensitive health promotion programmes targeted at ‘father inclusive’ practices and behaviour change communication programmes to stimulate social support may have positive changes in terms of the supportive role of partners. Implementors of such programmes should consider adopting innovative outreach strategies that appeal to young men’s interests and hang-out spots, such as bars and sports arenas**”.**
