# Supplementary material for: Perspectives about social support among unmarried pregnant university students in South Africa
Source: PLoS One. 2023 Apr 24;18(4):e0284906. doi: 10.1371/journal.pone.0284906 (PMC10124874; doi:10.1371/journal.pone.0284906)
Supplement: Attachment — Submitted filename: Response to reviewers.docx [file pone.0284906.s004.docx]

**Re:** **Perspectives about social support among unmarried pregnant university students in South Africa. PONE-D-22-16990R1**

Dear Editor,

A sincere thank you for your insightful comments on our manuscript. The comments will definitely help improve the manuscript. We have revised the paper in response to the editor’s comments.

The next section below contains our responses to the editor’s comments.

**#1:  The introductory section seems to assume that all university students who get pregnant are not married, likely in an unstable relationship with their partner, and that the pregnancy is unintended. This is not always going to be the case, so you may want to caveat this as “unmarried women with unintended pregnancies”, for example. At the very least, there needs to be a bit more nuance here, acknowledging that women get pregnant under different circumstances, including for women in university.**

Thank you for this comment. We have revised part of the introduction to indicate this caveat that the study focuses on unmarried women with unintended pregnancies. We have included literature that points to the high probability of unmarried university students having unintended pregnancies. We have explained in the manuscript that: “Although social support is of great value for all mothers during pregnancy, certain groups of women are especially vulnerable during pregnancy, and these include adolescents, unmarried women, students and women of low socio-economic status [7]. Because of the risky sexual behavior among students, young women in institutions of higher learning have a high risk of unintended pregnancy [8], particularly among unmarried students [9-11]”.

**#2: It would also be important to briefly mention the legality of abortion—you note that many students choose to terminate their pregnancies, but this is of course only likely/safe where abortion is, so if you could add a sentence about abortion within the South African context, that would be helpful. Although it is legal, I imagine it still carries certain stigma (and potentially different access barriers, making it an unavailable service for all women), so there may be women who are likely to continue with their pregnancy, even though they may prefer to terminate. In which case implications for social support are likely different when the pregnancy, though unintended, is not wanted, versus where it is wanted. As you note in your results, social supports like partner support mediate this (and the decision to terminate, if that is an option).**

We do agree that abortion may be associated with some stigma. We briefly describe the legality of abortion in the South African context. We explain that, “While some students may choose to terminate their unplanned pregnancies as a legal right stipulated under the Choice in Termination of Pregnancy Act in South Africa [12], abortion is often publicly condemned and associated with negative and judgmental attitudes from health care providers and the community [13, 14]. Students that choose to keep their unplanned pregnancies remain in need of a supportive environment that favours their physical and psychological wellbeing”.

**#3: I would ask that you broadly streamline the introduction, as there is quite a bit of repetition (social stigma, relationship with partner, etc. are raised multiple times).**

We have revised the introduction and removed some material which was repetitive.

**#4: In your methods, please given an indication of the types of questions that were asked.**

We had broadly included the themes from where the interview questions were drawn. Additionally, we included a few examples of the questions that comprised the interview schedule. In the manuscript we have added that: Some of the interview questions posed to participants include: “Describe some of the challenges you face in your pregnancy? As a pregnant student?”; “At what stage of pregnancy do you feel you need the most support from significant others? Why and from who?”; and “From the support needs you have already mentioned, which ones do you feel are the most important to you? Why?”.

**#5: Also, it would be useful to add if you inevitably sampled until theoretical saturation had been reached, which would suggest an added level of rigour.**

In the analysis section of the manuscript we have explained that, “Although 16 interviews already indicated a point of saturation in which no new codes were identified, we decided to continue analysis of the remaining interviews to ensure the confidence and verification of data saturation”.

**#6: Would it be possible to add to Table 2 if students were undergraduate or graduate students? As it’s clear you did recruit graduate students from the initial three, the breakdown by years seems to suggest only undergraduate students, which is confusing. It’s quite different to be a second year undergraduate versus a second year PhD student, for example. I think this would be an important detail that may affect their experience (especially given the very different demands at the undergraduate and postgraduate levels).**

Thank you for this suggestion. We have made the level of study of participants clearer by adding to the table that all 1^st^, 2^nd^ and 3^rd^ year participants were undergraduates. Only those who were doing an honours degree were post-graduates (or graduate) students.

**#7: I am not clear on how many themes you have! You say three, but then you have a theme about timing, and then about male support (which has its own sub-themes, I’m assuming the health system one is a sub-theme for this?)—so I’d say five? Please clarify.**

We explain in the findings that, “The findings are organized into four main themes and ten sub-themes identified from the analysis of the data. The four main themes are: Support needs of unmarried pregnant students; period when the most support is needed; male partner support required; and health systems role on social support”.

For clarity, we have listed the themes and sub-themes below:

**Support needs of unmarried pregnant students:** emotional support; instrumental support; informational support

**Period when the most support is needed:** at the beginning of pregnancy; throughout pregnancy; towards the end of pregnancy; support post delivery

**Male partner support required:** partner’s emotional support; partner’s instrumental support; partner’s financial support

**Health systems role on social support**

**#8: Please ensure the information about the participant giving each quotation is consistent (e.g. sometimes it’s age, sometimes it’s pregnancy/postpartum status, sometimes it’s educational level).**

We have provided the pregnancy and postpartum status of all participants and for those who have a previous pregnancy we include this detail for context. We also include the educational level of participants broadly as undergraduate or post-graduate.

**#9: Also ensure that** **all quotations in your results have a period at the end—I’d recommend inside of the quotation and before the information about the participant, as that is more grammatically correct.**

We have gone through the results and ensured that all quotations have a period at the end.

**#10: Where something was not consistently expressed and may be a divergent viewpoint, please make that clear. For example, you quoted one of your participants who was going to have a small baby, which you write as though nutritional challenges were consistent across your participants, but her experience is quite uncommon (for a baby to be small for gestational age to that extent, and especially due to nutrition—she would have had to be quite malnourished for this to be the case as the fetus will take what it needs and generally do well, as long as the maternal nutrition status was okay prior to pregnancy—if it was really poor, she’d likely not be menstruating and ovulating at all).**

Thank you for this comment. We have made it clear that the issue of nutritional challenges was unique. In the manuscript we have revised to read, “In a unique case, one of the participants was at risk of adverse pregnancy outcomes as she lacked adequate information on proper nutrition practices as illustrated below”.

**#11: For the timing query: I’d call these sub-themes, but I’d be clear about indicating an overarching theme, which, based on your results, seems to be something like, “timing of concentrated support in pregnancy varies depending on the participants’ circumstances”.**

The editor’s earlier comment was about the need to make the themes and sub-themes clear, which we have done. We do believe that the present themes and sub-themes we use correctly capture the essence of the study findings. With regards to this specific theme we had named it “Period when the most support is needed”, with the following sub-themes: *at the beginning of pregnancy*; *throughout pregnancy*; *towards the end of pregnancy*; and *support post-delivery.*

**#12: In your discussion, it’d be very interesting to see a reflection on why you feel appraisal support didn’t emerge.**

Thank you for this suggestion. We have added in the discussion that, “However, appraisal support did not emerge as an important source of social support in this study and this could be because most of the participants were still pregnant and felt they did not need evaluative feedback. Yet, this form of support could have been of great value to them after delivery as they engage in motherhood duties such as feeding and bathing the infant”.

**#13: You have some nice recommendations—it would also be useful to see some suggested areas for further research.**

Thank you for this suggestion. We added suggested areas for further research, “Future studies should include different racial groups to capture the experiences of pregnant students of all races and also include married and unmarried students to compare and contrast their needs.”

**#14: It would also be important to see a brief reflection on some of the strengths and limitations of your study, specifically your methodological approach, within the discussion.**

Thank you for this suggestion. We added the strengths and limitations of the study:

“To our knowledge, this is the first study to present a rich description of the perspectives about social support needs and the period when most support is needed by unmarried pregnant university students in South Africa. We also sampled until data saturation to ensure that a sufficient quantity and quality data was collected. However, the snowball method of sampling that was used meant that the initial participants, who were indigenous African students, recommended other pregnant students in their social network, who were also indigenous African students. As a result, potential participants from other racial groups did not participate in this study. Therefore, the findings could be biased and be a representation of experiences and perceptions of pregnant indigenous African students. Also, the findings of this study apply only to pregnant students who are unmarried and does not apply to those pregnant students who are in a marriage or engaged to be married. Future studies should include different racial groups to capture the experiences of pregnant students of all races and also include married and unmarried students to compare and contrast their support needs”.
